# Open-source cold and hot scientific sheet press for investigating polymer-based material properties

**DOI:** 10.1016/j.ohx.2024.e00566

**Published:** 2024-08-08

**Authors:** Morgan C. Woods, Cameron K. Brooks, Joshua M. Pearce

**Affiliations:** a Department of Mechanical and Materials Engineering, Western University, 1151 Richmond St., London, Ontario N6A 5B9, Canada; b Department of Electrical & Computer Engineering, Western University, 1151 Richmond St., London, Ontario N6A 5B9, Canada; c Ivey Business School, Western University, 1151 Richmond St., London, Ontario N6A 5B9, Canada

**Keywords:** Hot press, Sheet press, Materials processing, Polymer, Plastic, Composite

## Abstract

To produce samples for both material testing and molded sheets/parts, this article details an open-source scientific cold and hot press design. It consists of two independent and modular upper and lower plate (929 cm^2^) assemblies each containing four 125 W insulated steel strip heaters. The steel housing for these heaters is entirely modular and designed for ease of manufacture, assembly, and customization. This system allows a researcher with access to a hydraulic press to repurpose existing equipment into a multipurpose hot and cold press, or if an independent machine is warranted, an additional welded support frame and commercially available bottle jack offer standalone operation. By utilizing this small-scale hot press either in conjunction with a hydraulic press or on its own, samples can be produced to determine the critical material properties of any polymer, composite, or polymer blend. A series of modular molds allow for the rapid production of flat sheet stock and solid testing samples adhering to the ASTM D695 standard for rigid plastics tested in compression and ASTM D638 standard for testing plastics in tension. The sheet mold offers the user the ability to produce stock sheets that can be cut and assembled into 2.5-D applications with post processing.

## Graphical abstract

**Figure:**
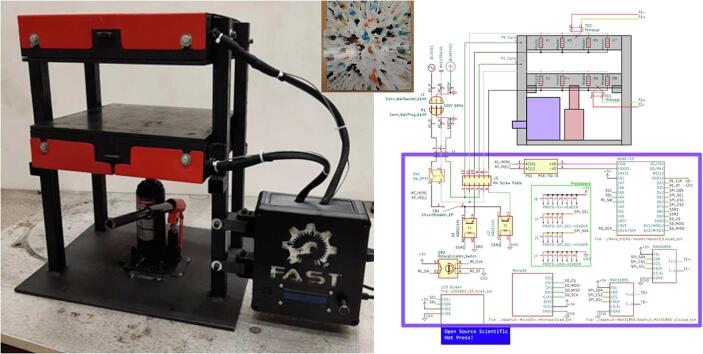

**Specifications table:** 

Hardware name	Open source high temperature scientific sheet press
Subject area	Engineering and materials science
Hardware type	Mechanical engineering and materials science
Closest commercial analog	5 Ton Portable Rosin Press Machine 2.3″ x 4.7′' Dual Heating Plate
Open source license	Designs GNU General Public License (GPL) 3.0 and hardware under CERN OHL v2
Cost of hardware	$652.36 CAD for Mechanical Hardware Assembly $363.81 CAD for Electrical Hardware Assembly and Enclosure $505.20 CAD for Resistive Heating Elements $77.21 CAD for Sheet Mold $104.33 CAD for Compression Testing Mold
Source file repository	https://osf.io/wqne2/; DOI https://doi.org/10.17605/OSF.IO/WQNE2
OSHWA certification UID *(OPTIONAL)*	Applying for certification – to be updated

## 1 Hardware in Context

Despite polymer recycling processes being well-known for decades, only about 9 % of all plastic produced by humanity has been recycled [1]. One of the primary factors contributing to this low plastic recycling rate is the economic disincentive. The current high costs associated with transporting, and recycling low-value, low-density plastics make it challenging for waste management companies to turn a profit on most plastics that enter their facilities and deter the corporations producing this waste from processing it for recycling instead of diverting it directly to a landfill [2]. For the average consumer, the primary motivations for recycling are environmental and ethical with minimal to no financial incentive. As a result, some locations have seen a complete halt to recycling all while global plastic production has continued to grow to 300 million tons per year [1]. To address this issue, a transition from industrial to distributed local recycling can be achieved using the two potentially scalable solutions discussed below.

First, as digital fabrication technologies like 3-D printing have matured, they have created a new opportunity for recycling that has shown environmental and economic promise. Post-consumer plastic waste can be upcycled into 3-D printing filament via recycled plastic filament extruders like the open-source recyclebots [3], [4], [5]. This approach, called distributed recycling and manufacturing (DRAM), provides consumers with a direct economic incentive to recycle [6], [7], [8], [9]. Prosumers (producing consumers) are incentivized to recycle their waste to produce usable filament in place of purchasing new material. These prosumers can then use recycled filament to directly support the fabrication of millions of consumer products from open-source designs for dramatically less than they can be purchased for from conventional manufacturing and distribution [10], [11], [12], [13]. Beyond economics, DRAM also cuts embodied energy of plastic additive manufacturing (AM) filament by 90 % thus substantially improving the environmental impact [14], [15], [16]. This approach has already shown viability for major AM feedstocks as either filament or direct particle extrusion [17], [18], [19] of polylactic acid (PLA) [20], [21], [22], [23] and acrylonitrile butadiene styrene (ABS) [1], [2], [3], [6], [16], [24], [25], [26], [27], but also common thermoplastics like high-density polyethylene (HDPE) [3], [28], [29], [30], low-density polyethylene (LDPE) [31], polypropylene (PP) [29], polystyrene (PS) [29], thermoplastic polyurethane (TPU) [4], [17], polyethylene terephthalate (PET) [32], [33] and polycarbonate (PC) [34]. In addition, PET, PP, and PS blends with compatibilizers can be achieved using DRAM [35], as well as PET/HDPE mixtures [36]. Finally, DRAM has been used for polymer composites including carbon-reinforced plastic [37], fiber-filled composites [38], [39], and various types of waste wood [40], [41]. Finally, more complex DRAM systems can use 3D-printed PC as molds for intrusion molding [34] for windshield wiper composites [42] or other materials that are difficult to 3-D print such as acrylonitrile styrene acrylate (ASA) and stamp sand waste composites [43]. DRAM can be profitable alone [44] but production capacity is limited by the relatively low production rate (e.g. a skateboard deck takes 11 h and 54 min to print).

A second distributed local recycling approach moves the recycling ownership from a single prosumer across a network. Developed and executed by Precious Plastics (an open hardware nonprofit organization originating in the Netherlands), this recycling model operates on community involvement to make plastic recycling accessible. Precious Plastics has designed and released build plans for a shredder, extruder, injection molder, and sheet press for converting large quantities of waste plastic into plastic regrind, tubing and bar stock, detailed batch products, and stock sheets (1 m^2^ x 4–35 mm thick [45]), respectively. Of this equipment, the sheet press provides the prosumer with the most design versatility and recycling volume as these large stock sheets can then be applied to any variety of project through simple resizing, cutting, profiling, and assembly. As a result of the potential impact, this hardware is emphasized as a viable method of contributing to distributed local recycling. Compression molding of plastics is a well-established method of processing plastics and relies simply on loading a charge of plastic into a mold and subjecting both to high pressure at an elevated temperature between the materials glass transition and melting point. This allows the plastic to flow freely and adopt the shape of the mold before cooling. To harness this methodology, the basic design of the Precious Plastics Sheet Press consists of 49 cartridge heaters [46] operating at 300 W spread across a fixed and variable position pressing plate (total 400 V, 32A, and 15 kW) to operate at a maximum temperature of 300° C [45]. This model offers a 1 m^2^ useable work area and is operated by first centering a silicon lubricated sheet metal blank on the heated platen followed by a 1 m^2^ welded square frame to hold the plastic regrind. This mold is then loaded with the appropriate volume of plastic by weight and covered using a second lubricated sheet metal cover. Once complete, an 8-ton long ram jack [46] is manually actuated to elevate the variable position lower plate until it fully compresses the mold between both plates. The system must remain heated for up to an hour [45] at which point the mold is cooled under pressure. The sheets produced are of visually high quality, however no formal material testing has been presented to confirm the material properties of the plastics used and the products made. As a result of using recycled plastic, the material properties of each batch are not precisely known, and the strength cannot be guaranteed for any load bearing applications. Further, the Precious Plastics sheet press relies on the availability of metric stock for material selection and ordering. This presents an additional obstacle when looking to fabricate these products in North America where the customary units in manufacturing remain imperial (Standard American English, SAE). For Canada specifically, while the country standard is formally metric, most metal manufacturers, suppliers, and tradesman continue to supply and operate with imperial stock and tools. Therefore, it is more efficient during the design and manufacturing process to use imperial stock and avoid costly materials, lead times, or post processing to accommodate designs relying on metric stock. This system is advertised to cost £ 2,550 (approximately $4260.53 CAD) to build [46] excluding all labor, machining and fabrication costs.

Outside and within the Precious Plastics community there exist a variety of design variations that have been shared by the members. These iterations include but are not limited to different heating elements, frame designs, material substitutions using local scrap, and a variety of machine sizes [47], [48], [49]. Similarly, users have shared their experiences and observations from using their equipment to identify process adjustments that improve the final product. A common trend across forums demonstrated people frequently seeking smaller, more manageable alternatives and settling upon using any combination of separate heating (ovens, T-shirt presses, irons, etc.) and pressure sources (vices, hydraulic jacks, clamps, etc.) to produce smaller sheets. This identifies a distinct lack of well-established alternatives available between extremely small or inappropriate machines being repurposed and the large footprint models such as the Precious Plastics Sheet Press. At smaller scales, expensive machines with uniquely small build plates such as the 5 ton Hydraulic Rosin Press Machine 2.3″ x 4.7″ inch Dual Heating Plates [50] restrict the users capabilities, and repurposed inappropriate equipment such as the Seeutek Heat press 12″ x 10″ Heat Transfer Digital Sublimation Machine [51] demand inconvenient work flows and additional processing steps.

Combining these models holds promise for achieving high value products from local waste plastic at scale by (a) using AM of high temperature polymers to make custom molds for a press and (b) building features on pressed sheet materials using AM. To support this promise, this article describes a scientific sheet press that can be used for (a) acquiring design information to build a larger sheet press for specific waste-plastic singular or composite materials, (b) material characterization studies of tensile and compression samples of polymer and polymer composites, and (c) producing small stock sheets for accessibly scaled prosumer prototyping and fabrication.

## 2 Hardware Description

**Fig. 1:**
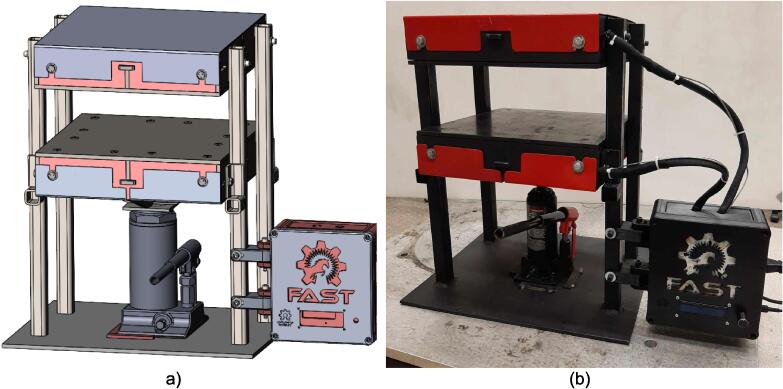
(a) Cold and hot scientific sheet press CAD assembly, (b) the realized cold and hot scientific sheet press.

A scaled open-source cold and hot scientific sheet press offers a simple alternative between slow, low volume recycling methods such as 3-D printing and large-scale commercial sheet production. A smaller plate size is ideal for manufacturing ASTM standard polymer-based testing samples for quantifying the material properties of waste plastic, and small to medium-scale molded products for commercialization. As the material properties of regrind waste plastic are often unknown, using it in structural and load bearing applications can provide substantial uncertainty. As such, it is beneficial to first quantify the material properties of the regrind being used on a smaller scale press. The commercial Rosin Press Machine provides one such market option for smaller scale hot pressing applications. The operating temperatures of these presses do not exceed 250° C and the work area is restricted to a 2.3″ x 4.7″ (69.74 cm^2^) footprint despite being outfitted with a comparably excessive 5 ton pressing capability [50]. As a result, mold size is limited and plastics with higher melting temperatures are either just out of reach or pushing the limits of the machine. The sized-up alternatives such as those offered by Precious Plastics are dramatically larger, more expensive, require much longer heating and cooling times, and introduce high power demands. To offer researchers and hobbyists alike the option to both produce samples for material testing as well as useable molded sheets/parts, a small scale hot and cold press was developed that provided tabletop accessibility without aggressively restricting the working area or loading capability. This press offers a 12in x 12in (929 cm^2^) pressing surface to fabricate any manner of plastic sheets for use in 2.5-D designs, pre-cast molds, and compression molded material testing samples. The technical specifications for this design are summarized in Table 1 below, and the final assembly shown in Fig. 1.

**Table 1:** Technical specifications of the open-source cold and hot scientific sheet press.

**Parameter**	**Description**
Power Supply	120 V 60 Hz Outlet
Power Consumption (maximum)	1000 W
Temperature Control and Range	PID control, Room Temperature – 350 °C
Size	12in x 12in (30.5 cm x 30.5 cm) for pressing plates only. 13 ¼in x 16 ¼in x 19 ½in (33.7 cm x 41.3 cm x 49.5 cm) for independent machine
Total Weight	85.3lbs (38.70 kg)
Loading Capacity	8 tons (7258 kg) in a hydraulic press4 tons (3628 kg), bottle jack limiting as an independent machine.

To achieve this, the cold and hot scientific sheet press design consists of two independent and modular upper and lower plate assemblies each containing four 125 W Mica insulated steel strip heaters for a nominal power draw of 1000 W. The steel housing for these heaters was designed for ease of manufacture, assembly, and customization. the majority of parts were designed to be universal between both plate assemblies and reversible to reduce the number of unique design files required and potential assembly error. Similarly, the mechanical design is motivated by an interest in minimizing the number of tools and operations required. Laser cut plate steel was predominantly used so that the assembly method could rely on interlocking flat patterns to create 2.5-D shapes that reduced the number of required fasteners and welds. These simple patterns and interlocking approach also promote assembly customization wherein the user can choose to add or remove components depending on their unique loading requirements and budget. Further reducing costs, this press allows a researcher with access to an existing hydraulic press to construct only the pressing plates and repurpose their equipment into a multipurpose hot and cold press. In this case, the manufacturer would not need to invest in additional hydraulics or framing to make a standalone machine. If a hydraulic press is not available, or the consumer is interested in an independent tabletop machine, the design includes a simple welded support frame and commercially available bottle jack to support the pressing plates. By utilizing this small-scale hot press either in conjunction with a hydraulic press or on its own, ASTM standard samples can be produced to determine the critical material properties of any polymer or polymer blend before committing to a large-scale fabrication method.

Separate from the assembly, a series of modular molds were developed in conjunction with the press to offer users the ability to produce material characterizing solid testing samples adhering to the ASTM D695 standard for rigid plastics tested in compression, ASTM D638 standard for testing plastics in tension, and the production of flat sheet stock. With 12 samples produced at once using the ASTM D695 mold, and 6 samples in the ASTM D638 mold, a variety of compositions can be achieved and tested simultaneously without increasing manufacturing time or effort. The sheet mold offers the user the ability to produce stock sheets that can be cut and assembled into 2.5-D applications with post processing.

The electrical system was designed to be minimalistic, adaptable, and scalable, while being centered around an open-source microcontroller architecture that includes only the core components necessary for monitoring and controlling the temperature of each plate. This foundational simplicity allows scientists to tailor and enhance the systems functionality with a broad array of standard off-the-shelf or specialized components. The open-source nature of the microcontroller also encourages seamless integration of a wide array of sensors and devices. These sensors can range in complexity from simple pressure and force measurement tools, additional temperature sensors for materials or molds, ambient condition monitors, and thermal imaging cameras, to advanced solutions including material flow and viscosity gauges, optical sensors for quality assessment, gas analysis detectors for safety and environmental compliance, and conductivity sensors for materials research. The introduction of these sensors can further enhance the machines potential as a versatile platform for detailed process analysis and materials research. This open, modular approach ensures that researchers and developers can easily customize the press to support a wide spectrum of scientific inquiries and industrial applications, from the examination and utilization of recycled plastics to the innovation of new composite materials.

The electrical enclosure was likewise designed with manufacturing and assembly in mind. The enclosure functions as both a housing for all the high power and low power electrical components, as well as a human-user interface (HMI) for offline operation and monitoring. The enclosure was designed to minimize the number and complexity of components needed. As a result, it relies on a 3-D printed body and lid to mount the electrical components and provide an HMI respectively, followed by a simple and easy to remove flat rear cover to isolate the connections. This design provides the user with the versatility to either 3-D print all components – including the rear cover – or form this cover with the press as a first trial sheet. Keeping this cover independent of the electronics, the user can easily access the electronics for diagnosis with only the removal of two screws. Further, the enclosure includes holes to accommodate custom Mounting Brackets for integrating the electronics with the sheet press assembly. These brackets can easily be modified to accommodate different design iterations and include a pin-locking swivel joint to either display the HMI or fold the assembly flush against the legs of the sheet press.

Benefits of the novel design:

• Using custom molds, the system offers users a method of fabricating solid polymer-based samples to promote characterization of unknown material properties.
• The scale of the design bridges the gap between inappropriate commercial designs and industrial scale presses to target small scale production applications for repurposing waste plastic.
• The system can be used to rapidly produce new or existing material blends at varied element ratios to test ideal compositions.
• The design was tailored to accommodate imperial North American standards to reduce stock costs for consumers as well as offering further cost reduction opportunities by providing alternative configurations that repurpose existing lab room equipment to eliminate optional components.
• The programmable and scalable design caters to the varied and evolving requirements of diverse user groups, while ensuring compatibility with new technologies to prevent obsolescence.
• The ‘tuning mode’ allows for more precise control over the sheet press's operations, which can translate to better quality and consistency in sample production.
• With integrated data collection, the system enables real-time monitoring of production parameters, facilitating immediate adjustments and improvements in the process as well as sharing of standardized data for collaborative improvements.
• Equipped with a removable storage device, the system logs temperature, PID coefficients, and status over time, aiding in precise material and process optimization for improved quality and efficiency.

## 3 Design Files

### 3.1 Design Files Summary

The design consists of predominantly laser cut steel components used to form welded subassemblies, square steel extrusion, steel angle, and 3-D printed electronics housings and brackets. All components that cannot be purchased directly and must be custom fabricated are summarized below in Table 2 along with the necessary links to locate these files and reproduce them. The components are further broken down to identify which subassemblies they belong to for future referencing throughout this paper.

**Table 2:** Design Files Summary.

**Item**	**Design file name**	**Subassembly**	**File type**	**Open-source license**	**Location of the file**
1	Center Support	Upper Plate Assembly	SLDPRT, STEP, DXF	GNU General Public License (GPL) 3.0	https://osf.io/7nq8u https://osf.io/g2uy8 https://osf.io/yz8ng
2	Interlocking Tab	Upper AND Lower Plate Assembly	SLDPRT, STEP, DXF	GNU General Public License (GPL) 3.0	https://osf.io/7v5es https://osf.io/3xzvf https://osf.io/zb5mj
3	Jack Support L1	Lower Plate Assembly	SLDPRT, STEP, DXF	GNU General Public License (GPL) 3.0	https://osf.io/vcb3a https://osf.io/nxps8 https://osf.io/w8p5f
4	Jack Support U1	Lower Plate Assembly	SLDPRT, STEP, DXF	GNU General Public License (GPL) 3.0	https://osf.io/3zrmh https://osf.io/ermbd https://osf.io/zgbj9
5	Outer Framing Wall	Welded Frame	SLDPRT, STEP, DXF	GNU General Public License (GPL) 3.0	https://osf.io/pmh5q https://osf.io/c6spr https://osf.io/fs9qk
6	Upper Steel Platen	Upper Plate Assembly	SLDPRT, STEP, DXF	GNU General Public License (GPL) 3.0	https://osf.io/5ht9d https://osf.io/tkx8a https://osf.io/4cdyx
7	Lower Steel Platen	Lower Plate Assembly	SLDPRT, STEP, DXF	GNU General Public License (GPL) 3.0	https://osf.io/nmhkj https://osf.io/9mqc7 https://osf.io/b2du9
8	Steel Platen	Upper AND Lower Plate Assembly	SLDPRT, STEP, DXF	GNU General Public License (GPL) 3.0	https://osf.io/5vzqd https://osf.io/avk2r https://osf.io/63gxb
9	Mounting Bracket	Upper And Lower Plate Assembly	SLDPRT, STEP	GNU General Public License (GPL) 3.0	https://osf.io/9zy3u https://osf.io/v749z
10	Upper Sheet Metal Enclosure	Upper Plate Assembly	SLDPRT, STEP	GNU General Public License (GPL) 3.0	https://osf.io/5pyu6 https://osf.io/qbmchhttps://osf.io/yuqf8
11	Upper Sheet Metal Enclosure Flat Pattern	Upper Plate Assembly	STEP, DXF	GNU General Public License (GPL) 3.0	https://osf.io/e6mfj https://osf.io/dybks
12	RH Lower Sheet Metal Enclosure	Lower Plate Assembly	SLDPRT, STEP	GNU General Public License (GPL) 3.0	https://osf.io/3av27 https://osf.io/eytfv
13	LH Lower Sheet Metal Enclosure	Lower Plate Assembly	SLDPRT, STEP	GNU General Public License (GPL) 3.0	https://osf.io/n2qbz https://osf.io/xsfhb
14	Lower Sheet Metal Enclosure Flat Pattern	Lower Plate Assembly	STEP, DXF	GNU General Public License (GPL) 3.0	https://osf.io/snpa2 https://osf.io/j6mfz
15	Assembly Base Plate	Supporting Frame	SLDPRT, STEP, DXF	GNU General Public License (GPL) 3.0	https://osf.io/uc8pb https://osf.io/2rudk https://osf.io/d96gm
16	Support Leg	Supporting Frame	SLDPRT, STEP	GNU General Public License (GPL) 3.0	https://osf.io/96njz https://osf.io/vaqk8
17	Electrical Enclosure Support Leg	Supporting Frame	SLDPRT, STEP	GNU General Public License (GPL) 3.0	https://osf.io/mgbjt https://osf.io/nkhe8
18	Lower Plate Support	Supporting Frame	SLDPRT, STEP	GNU General Public License (GPL) 3.0	https://osf.io/7aqek https://osf.io/4zupv
19	Linear Guide	Supporting Frame	SLDPRT, STEP	GNU General Public License (GPL) 3.0	https://osf.io/rhbgp https://osf.io/dw8yz
20	Jack Spacer	Supporting Frame	SLDPRT, STEP, DXF	GNU General Public License (GPL) 3.0	https://osf.io/m25kh https://osf.io/ch2v5 https://osf.io/rpx7u
21	Jack Locating Guide	Supporting Frame	SLDPRT, STEP, DXF	GNU General Public License (GPL) 3.0	https://osf.io/v8qtu https://osf.io/ztng6 https://osf.io/n9s8q
22	Welded Frame	Welded Frame	STEP	GNU General Public License (GPL) 3.0	https://osf.io/unk7x
23	Upper Plate Assembly	Upper Plate Assembly	STEP	GNU General Public License (GPL) 3.0	https://osf.io/96qzc
24	Lower Plate assembly	Lower Plate Assembly	STEP	GNU General Public License (GPL) 3.0	https://osf.io/uhsvt
25	Sheet Press Assembly	Sheet Press Assembly	SLDASM (zip),STEP	GNU General Public License (GPL) 3.0	https://osf.io/k6tfqhttps://osf.io/nbxzq
26	ASTM D695 Mold Lid	ASTM D695 Mold Assembly	SLDPRT, STEP, DXF	GNU General Public License (GPL) 3.0	https://osf.io/mvcb8 https://osf.io/wsydp https://osf.io/mzv5n
27	Plug	ASTM D695 Mold Assembly	SLDPRT, STEP, DXF	GNU General Public License (GPL) 3.0	https://osf.io/e6tc2 https://osf.io/7rbae https://osf.io/f2dwa
28	ASTM D695 Mold	ASTM D695 Mold Assembly	SLDPRT, STEP, DXF	GNU General Public License (GPL) 3.0	https://osf.io/becr7 https://osf.io/qr4m9 https://osf.io/hvy6r
29	ASTM D638 Mold	ASTM D638 Mold	SLDPRT, STEP, DXF	GNU General Public License (GPL) 3.0	https://osf.io/f57ug https://osf.io/ka875 https://osf.io/j68mf
30	Surface Sheet	Sheet Mold	SLDPRT, STEP, DXF	GNU General Public License (GPL) 3.0	https://osf.io/nkfwu https://osf.io/tw5va https://osf.io/9gn3s
31	Short Framing Wall	Sheet Mold	SLDPRT, STEP, DXF	GNU General Public License (GPL) 3.0	https://osf.io/zb7y4 https://osf.io/kpgj5 https://osf.io/z6d8b
32	Long Framing Wall	Sheet Mold	SLDPRT, STEP, DXF	GNU General Public License (GPL) 3.0	https://osf.io/8uwce https://osf.io/pwtfx https://osf.io/vtx5m
33	Sheet Mold Frame	Sheet Mold	SLDPRT, STEP	GNU General Public License (GPL) 3.0	https://osf.io/y254s https://osf.io/f5z3r
34	Electrical Enclosure Body	Electrical Enclosure	STEP, STL, 3mf, GCODE File	GNU General Public License (GPL) 3.0	https://osf.io/wtxyr https://osf.io/c5vpx https://osf.io/fcsnqhttps://osf.io/hyjg9
35	Electrical Enclosure Lid	Electrical Enclosure	STEP, STL, 3mf, GCODE File	GNU General Public License (GPL) 3.0	https://osf.io/vyza7 https://osf.io/etqya https://osf.io/ys2rc https://osf.io/tde5q
36	Solid Electrical Enclosure Lid	Electrical Enclosure	STEP, STL, 3mf, GCODE File	GNU General Public License (GPL) 3.0	https://osf.io/ugpwf https://osf.io/bz4c8 https://osf.io/2swyx https://osf.io/37acs
37	Electrical Enclosure Mounting Bracket	Electrical Enclosure	STEP, STL, 3mf, GCODE File	GNU General Public License (GPL) 3.0	https://osf.io/dxu7g https://osf.io/p4gyx https://osf.io/3p59bhttps://osf.io/pk8e2
38	Electrical Enclosure Mount	Electrical Enclosure	STEP, STL, 3mf, GCODE File	GNU General Public License (GPL) 3.0	https://osf.io/y8kz9 https://osf.io/gah52 https://osf.io/3p59bhttps://osf.io/pk8e2
39	Electrical Enclosure Rear Cover	Electrical Enclosure	STEP, STL, 3mf, GCODE File	GNU General Public License (GPL) 3.0	https://osf.io/te3gv https://osf.io/hb8zf https://osf.io/6vhpg https://osf.io/59dty
40	PSU Bracket	Electrical Enclosure	STEP, STL, 3mf, GCODE File	GNU General Public License (GPL) 3.0	https://osf.io/cq8n4 https://osf.io/fpd4c https://osf.io/3p59bhttps://osf.io/pk8e2
41	Nano Bracket	Electrical Enclosure	STEP, STL, 3mf, GCODE File	GNU General Public License (GPL) 3.0	https://osf.io/pjdbh https://osf.io/br2tu https://osf.io/3p59b https://osf.io/pk8e2
42	Electrical Enclosure Assembly	Electrical Enclosure	STEP	GNU General Public License (GPL) 3.0	https://osf.io/mnwbd

Note: All items available in SLDPRT and STEP for modification and viewing, DXF for necessary laser cutting and vendor quoting. All items available as an STL for printer adaptation and 3mf and G-CODE Files for exact settings.

*Item 1:* The Center Support reinforces the upper plate assembly and distributes force across the upper platen.

*Item 2:* The Interlocking Tabs secure the Center Supports/Jack Supports to the welded assembly for both the upper and lower plate assemblies.

*Item 3:* The Jack Supports reinforce the lower plate assembly and distribute the load of the jack across the lower platen. L1 indicates the lower notch for interlocking with Item 4.

*Item 4:* The Jack Supports reinforce the lower plate assembly and distribute the load of the jack across the lower platen. U1 indicates the upper notch for interlocking with Item 3.

*Item 5:* The repeating Outer Framing Walls are used in the Welded Assembly and make up the perimeter of the upper and lower frame assemblies.

*Item 6:* The Upper Steel Platen acts as the interface between the upper plate assembly and the molds. The Upper Steel Platen has a hole pattern unique to the heating elements and orientation used in this build.

*Item 7:* The Lower Steel Platen acts as the interface between the lower plate assembly and the molds. The Lower Steel Platen has a hole pattern unique to the heating elements and orientation used in this build.

*Item 8:* The Steel Platen acts as the interface between the upper and lower plate assemblies and the molds. The Steel Platen has no hole pattern and is not unique to the upper or lower plate assemblies. This can be tailored for use with either different elements or a different mounting method.

*Item 9:* The Mounting Brackets secure the Upper and Lower Steel Platens to the welded assembly for both the Upper and Lower Plate Assemblies.

*Item 10:* The Upper Sheet Metal Enclosure secures the insulation within the Upper Plate Assembly. For this build, it also secures the heating elements in place with the insulation.

*Item 11:* The Upper Sheet Metal Enclosure Flat Pattern is the cut pattern for the Upper Sheet Metal Enclosure prior to bending. It provides the appropriate hole pattern and identifies the bending lines necessary for bending.

*Item 12:* The RH Lower Sheet Metal Enclosure secures the insulation within two opposite quadrants of the Lower Plate Assembly. For this build, it also secures the heating elements in place with the insulation.

*Item 13:* The LH Lower Sheet Metal Enclosure is the mirror of Item 12 and is used to cover the remaining two quadrants of the Lower Plate Assembly.

*Item 14*: The Lower Sheet Metal Enclosure Flat Pattern is the cut pattern for the Lower Sheet Metal Enclosure prior to bending. It is the same for both the LH and RH versions (Item 12 and 13), and only differs once bent.

*Item 15:* The Assembly Base Plate provides the foundation of the machine to both transfer the load from the bottle jack and allow it to operate as a standalone machine.

*Item 16:* The three Support Legs provide both the clearance for the jack to be housed, as well as the guide rails to constrain the linear motion of the Lower Plate Assembly.

*Item 17*: The Electrical Enclosure Support Leg provides the same function as Item 16, but includes the addition of two through holes for mounting the Electrical Enclosure.

*Item 18:* The Lower Plate Support provides a hard stop for the Lower Plate Assembly to rest on when the machine is not in use and for ease of jack removal and operator safety.

*Item 19:* The Linear Guides constrain the motion of the lower plate assembly to act exclusively along the parallel Support Legs.

*Item 20:* The Jack Spacer is an optional component that ensures the jack has a stable, level surface to act on.

*Item 21:* The Jack Locating Guides are welded to the base plate and ensure the jack can be removed and repeatably positioned each time it is replaced.

*Item 22:* The Welded Frame is the perimeter of the Upper and Lower Plate Assemblies consisting of the Outer Framing Walls.

*Item 23:* The Upper Plate Assembly with Center Supports and Upper Sheet Metal Enclosure.

*Item 24:* The Lower Plate Assembly with Jack Supports and lower Sheet Metal Enclosure.

*Item 25:* The Sheet Press Assembly contains a complete model of the standalone open-source cold and hot scientific sheet press.

*Item 26:* The ASTM D695 Mold Lid acts as the top and bottom sandwiching plates of the testing mold used to create blanks of pressed waste plastic for compression testing.

*Item 27:* The mold Plugs are used to compress and form the heated waste plastic within the ASTM D695 Mold.

*Item 28:* The ASTM D695 Mold is the pocketed structure used to form twelve equal sized compression testing blanks of waste plastic.

*Item 29:* The Surface Sheets of the Sheet Mold provide the upper and lower constraints for solid plastic plates.

*Item 30:* The ASTM D638 Mold is the form for producing Type 1 dog bone tensile testing samples.

*Item 31:* The Short Framing Walls of the sheet mold make up the inside half of the Sheet Mold Frame.

*Item 32:* The Long Framing Walls of the sheet mold make up the outside half of the Sheet Mold Frame.

*Item 33:* The Sheet Mold Frame is the welded enclosure for constraining the size and shape of solid plastic sheets.

*Item 34:* The Electrical Enclosure Body provides a stable platform for housing all of the electrical components and a structural body for integrating the electrical system with the Support Frame.

*Item 35:* The Electrical Enclosure Lid conceals all high-power terminals and exposed bolts from mounting the electrical components. It features cutouts for a Liquid Crystal Display (LCD) and a rotary encoder knob to offer a human–machine interface (HMI). This version has a through-all window that allows users to mount customizable artistic covers from recycled plastic sheets fabricated in the sheet press while saving filament, print time, and money.

*Item 36:* The Solid Electrical Enclosure Lid offers the same features as item 35, but has a simplified design to prevent users from accessing the high-power terminals in a single 3-D printed part. It does not require the fabrication of an additional component.

*Item 37:* The Electrical Enclosure Mounting Brackets secure to the outside of the Electrical Enclosure to offer a connection point for the Electrical Enclosure Mounts. This intermediate connection between the enclosure and the Support Leg introduces the ability to swivel the Electrical Enclosure between two positions.

*Item 38:* The Electrical Enclosure Mount acts as the interface between the Electrical Enclosure Mounting Brackets and the Electrical Enclosure Support Leg.

*Item 39:* The Electrical Enclosure Rear Cover seals off the internal electrical components and prevents the user from accessing any wired connections. This component can be pressed using recycled plastic to offer an artistic option or 3-D printed*.*

*Item 40:* The PSU Bracket mounts the Power Supply Unit (PSU) module securely to the Electrical Enclosure Body.

*Item 41:* The Nano Bracket mounts the Arduino securely to the Electrical Enclosure Body*.*

*Item 42:* The Electrical Enclosure Assembly features the Electrical Enclosure with all 3-D printed components integrated.

## 4 Bill of Materials (BOM)

The following section outlines a complete list of all materials needed to build the standalone cold and hot scientific sheet press and their respective costs. The materials have been separated first into Table 3 to isolate the components that contribute to the mechanical assembly and require manufacturing techniques not limited to laser cutting, welding, drilling, and simple hand tool use. It is important to note that the cost for all laser cut components includes *both* the material cost *and* laser cutting as this is the only manufacturing operation that demands accuracy with comparatively complex parts. All other parts are defined only by their material cost and are not associated with any additional labor/tooling costs as these were absorbed by simple manufacturing methods and did not need to be outsourced. Following the manufacturing BOM, Table 4 and Table 5 outline the cost required to assemble the electrical schematic and 3-D print each component of the associated electrical enclosure, respectively.

**Table 3:** Cold and Hot Scientific Sheet Press Manufactured Bill of Materials.

**Component Designator**	**Description**	**Sub-assembly**	**Quantity**	**Cost per unit CAD**	**Total cost** **CAD**	**Source of materials**	**Material type**
Center Support	Custom Laser Cut Part, 1/4″ Thick	Upper Plate Assembly	2		271.05	Diversified Metals Inc. Material and Service	Mild Steel
Interlocking Tab	Custom Laser Cut Part, 1/4″ Thick	Upper AND Lower Plate Assembly	8			Diversified Metals Inc. Material and Service	Mild Steel
Jack Support L1	Custom Laser Cut Part, 1/4″ Thick	Lower Plate Assembly	1			Diversified Metals Inc. Material and Service	Mild Steel
Jack Support U1	Custom Laser Cut Part, 1/4″ Thick	Lower Plate Assembly	1			Diversified Metals Inc. Material and Service	Mild Steel
Outer Framing Wall	Custom Laser Cut Part, 1/4″ Thick	Welded Frame	8			Diversified Metals Inc. Material and Service	Mild Steel
Upper Steel Platen	Custom Laser Cut Part, 1/4″ Thick	Upper Plate Assembly	1			Diversified Metals Inc. Material and Service	Mild Steel
Lower Steel Platen	Custom Laser Cut Part, 1/4″ Thick	Lower Plate Assembly	1			Diversified Metals Inc. Material and Service	Mild Steel
Mounting Bracket	1 1/2″ x 1 1/2″ x 1/8″Angle, Material Only	Upper AND Lower Plate Assembly	8	1.57	12.57	Metal Supermarkets	Mild Steel
Upper Sheet Metal Enclosure	12″ x 16″ x0.036″ (20 gauge) custom sheet	Upper Plate Assembly	1	23.76	23.76	Metal Supermarkets	Cold Rolled Mild Steel
RH Lower Sheet Metal Enclosure	12″ x 16″ x0.036″ (20 gauge) custom sheet	Lower Plate Assembly	2	7.95	15.90	Metal Supermarkets	Cold Rolled Mild Steel
LH Lower Sheet Metal Enclosure	12″ x 16″ x0.036″ (20 gauge) custom sheet	Lower Plate Assembly	2	7.95	15.90	Metal Supermarkets	Cold Rolled Mild Steel
Assembly Base Plate	16 1/4″ x 13 1/4″ x1/4″ thick plate	Supporting Frame	1	76.74	76.74	Metal Supermarkets	Hot Rolled Mild Steel
Support Leg	1″ x 1″ x 1/8″ thick x 19″ long square tubing	Supporting Frame	3	10.10	40.40	Metal Supermarkets	Structural Mild steel
Electrical Enclosure Support Leg	1″ x 1″ x 1/8″ thick x 19″ long square tubing	Supporting Frame	1	10.10		Metal Supermarkets	Structural Mild Steel
Lower Plate Support	1″ x 1″ x 1/8″ thick x 1″ long square tubing	Supporting Frame	4	Cut 1″ length off of each 20″ length leg ordered		Metal Supermarkets	Structural Mold Steel
Linear Guide	3/16″ thick 1″ x 1″ x 2″ long Angle	Supporting Frame	4	2.09	8.36	Metal Supermarkets	Mild Steel Angle
Jack Spacer	4″ x 3″ x 3/16″ plate	Supporting Frame	1	Order as one 4″ x 10″ x 3/16″ sheet and cut to size	40.67	Metal Supermarkets	Hot Rolled Mild Steel
Jack Locating Guide	1/2″ x 3″ x 3/16″ thick bar	Supporting Frame	2			Metal Supermarkets	Hot Rolled Mild Steel
ASTM D695 Mold Lid	Custom laser cut 1/8″ Sheet	ASTM D695 Mold Assembly	2	5.38	10.76	ABUMA Manufacturing – Material and service	Aluminum
Plug	Custom laser cut 1/2″ Plate	ASTM D695 Mold Assembly	12	5.42	65.04	ABUMA Manufacturing – Material and service	Aluminum
ASTM D695 Mold	Custom laser cut 1″ Plate	ASTM D695 Mold	1	28.53	28.53	ABUMA Manufacturing – Material and service	Aluminum
Surface Sheet	13″ x 11 7/8″ x 0.063″ (H14) custom sheet	Sheet Mold	2	23.42	46.84	Metal Supermarkets	6061 Aluminum
Short Framing Wall	1/2″ x 11 1/4″ x 1/4″ Thick	Sheet Mold	2	Order as one 12 ¼” x 4″ x ¼” sheet and cut to size	30.37	Metal Supermarkets	Mild Steel
Long Framing Wall	1/2″ x 12 1/4″ x 1/4″ Thick	Sheet Mold	2			Metal Supermarkets	Mild Steel
1/4–20 x 1″ FHCS	1/4–20 x 1″ Flat Head Cap Screw	Upper AND Lower Plate Assembly	26	0.54	14.04	Facca Fasteners	Steel; Black Oxide Coating
1/4″ Nut	1/4–20 Standard Hex Nut G5		46	0.10	4.60	Facca Fasteners	Grade 5 Zinc
1/4–20 x 1″ HHCS	1/4–20 x 1″ Hex Head Cap Screw G5	Upper AND Lower Plate Assembly	14	0.18	2.48	Fastenal	Grade 5 Zinc
1/4–20 x 1 3/4″ SHCS	1/4–20 x 1 3/4″ Socket Head Cap Screw G5	Supporting Frame	4	0.69	2.76	Facca Fasteners	Grade 5 Zinc
3/16″ Washer	3/16″ x 0.562″ OD General Purpose Flat Washer		60	0.05	2.70	Fastenal	Low Carbon Zinc Finish Steel
Bottle Jack	MotoMaster 4-Ton Hydraulic Bottle Jack	Supporting Frame	1	29.99	29.99	Canadian Tire	Steel
Insulation	Rockwool ComfortBatt R22 Insulation	Upper AND Lower Plate Assembly	0.1	84.85	8.49	RONA	Stone wool insulation
Machine Lubricant	Rotella Special Duty Moly Grease	Supporting Frame	1	14.99	14.99	Canadian Tire	Molybdenum disulfide solid lubricant
Mold Lubricant	3-in-One Professional Water- Resistant Lubricant		1	11.99	11.99	Canadian Tire	Silicone based lubricant
Primary Paint	Rust-Oleum High Heat Spray Paint, Matte Black		1	17.99	17.99	Canadian Tire	Aerosol Spray Paint
Accent Paint	Armor Coat Interior/ Exterior Rust Paint Fire Red Gloss		1	24.99	24.99	Canadian Tire	Aerosol Spray Paint
Thermal Adhesive	JB Weld 37,906 Muffler Seal Paste		1	11.99	11.99	Canadian Tire	Cement Paste

*Cost may vary depending on manufacturer, and available resources. Costs for the Support Legs, top and Lower Sheet Metal Enclosures, and Sheet Mold are estimated based on market prices as scrap metal was used for this build. Taxes not included outside of Diversified Metals Inc. quoted orders.

**Table 4:** Cold and Hot Scientific Sheet Press Electrical Enclosure and Hardware Bill of Materials (BOM).

**Component Designator**	**Description**	**Quantity**	**Cost per Unit**	**Total cost CAD**	**Source of materials**	**Material type**
Flat Heating Element	125 W, 120 V, Steel Strip Heater	8	63.15	505.20	Omega	Rust-resistance Steel, Mica Insulation
Arduino Nano	Arduino Nano ESP32	1	29.20	29.20	Digikey	Electronics
Thermocouple Sensor (+Prototyping Amp)*	High Temperature Sensor (K-Type, 800 °C)	2	24.90	49.80	DFRobot	Electronics
Thermocouple Amplifier	MAX31855 Break out	2	14.95	29.90	Adafruit	Electronics
Solid State Relay	SSR RELAY SPST-NO 15A 75–250 V	2	29.56	59.12	Digi Key	Silicon, epoxy, aluminum
M4 Ring Terminal	M4 Insulated ring terminal Electrical wire crimp connectors	0.16	9.89	1.58	Amazon	Tin-plated Copper, PVC
Black Solid Wire	Solid Wire 300 V AC, 20 Wire Gauge, Black, 25ft	0.56	5.13	2.87	McMaster	Copper, PVC
White Solid Wire	Solid Wire 300 V AC, 20 Wire Gauge, White, 25ft	0.56	5.13	2.87	McMaster	Copper, PVC
Green Solid Wire	Solid Wire 300 V AC, 20 Wire Gauge, Green, 25ft	0.56	5.13	2.87	McMaster	Copper, PVC
Black Stranded Wire	16 Gauge Machine Tool Wire, Black, 25ft	0.48	7.50	3.60	McMaster	Copper, PVC
White Stranded Wire	16 Gauge Machine Tool Wire, White, 25ft	0.32	7.50	2.40	McMaster	Copper, PVC
Green Stranded Wire	16 Gauge Machine Tool Wire, Green, 25ft	0.32	7.50	2.40	McMaster	Coper, PVC
High-Temperature Hook-up Wire	High-Temperature Stranded Wire with Fiberglass Outer and Mica Inner Insulation, 18 Gauge; Length, ft. 50	0.2	85.50	17.10	McMaster	Tin-plated Copper, Fiberglass, Mica, PTFE
Circuit Breaker	W28-XQ1A-14, 14A, Thermal type Circuit Breaker	1	7.44	7.44	Digikey	Copper/Silver, Thermoset Polymer
AC Power Cord	14AWG NEMA5-15P − IEC320 3′ Power Cord	1	12.91	12.91	Digikey	Copper, PVC
Power Entry Receptacle	AC Power Entry Modules IEC-320C-14	1	16.59	16.59	Mouser	Copper/Silver, Thermoset Polymer
Jumper Wires	120pcs Multicolored Dupont Wire 40pin	0.25	13.99	3.50	Amazon	Copper, PVC, ABS
LCD Display	SunFounder IIC/I2C/TWI 1602 Serial LCD	1	13.59	13.59	Amazon	Electronics
Rotary Encoder Dial	Model KY-040, 5 V, 20 PPR Rotary Encoder Dial	0.2	17.40	3.48	Amazon	Electronics
AC/DC Converter PSU	PSK-5D-9 – AC/DC Module	1	9.80	9.80	Mouser	Electronics
SD Card	SAMSUNG EVO Select Micro SD-Memory-Card	1	20.05	20.05	Amazon	Electronics
SD Card Reader	Adafruit MicroSD breakout board PCB	1	10.68	10.68	Digi Key	Electronics
Cable Sheath	Techflex Insultherm Tru-Fit Braided Fiberglass Sleeving	0.04	64.66	2.59	cablestiesandmore	Fibreglass
PETG Filament	Polymaker PETG Filament 1.75 mm, 1 kg	0.4	28.99	11.60	Amazon	Thermoplastic Polymer
M2 x 15 BHCS	M2 x 15 Hex Button Head Socket Cap Screw	4		10.09	Amazon	304 Stainless Steel
M2 x 20 BHCS	M2 x 20 Hex Button Head Socket Cap Screw	4			Amazon	304 Stainless Steel
M2 Nut	M2 Standard Hex Nut	8			Amazon	304 Stainless Steel
M3 x 10 BHCS	M3 x 10 Hex Button Head Socket Cap Screw	8			Amazon	304 Stainless Steel
M3 x 15 BHCS	M3 x 15 Hex Button Head Socket Cap Screw	6			Amazon	304 Stainless Steel
M3 x 20 BHCS	M3 x 20 Hex Button Head Socket Cap Screw	6			Amazon	304 Stainless Steel
M3 Nut	M3 Standard Hex Nut	20			Amazon	304 Stainless Steel
M4 x 10 BHCS	M4 x 10 Hex Button Head Socket Cap Screw	4			Amazon	304 Stainless Steel
M4 x 15 FHCS	M4 x 15 FHCS Flat Head Cap Screw	6			Amazon	304 Stainless Steel
M4 Nut	M4 Standard Hex Nut	22			Amazon	304 Stainless Steel
1/4–20 x 1 3/4″ Socket Head Cap Screw SHCS	1/4–20 x 1 3/4″ Socket Head Cap Screw	2	0.49	0.98	Fastenal	Steel; Black Oxide Coating
3/16″ Washer	3/16″ x 0.562″ OD General Purpose Flat Washer	4	0.05	0.20	Fastenal	Low Carbon Zinc Finish Steel
1/4″ Nut	1/4–20 Standard Hex Nut G5	2	0.11	0.22	Facca Fasteners	Grade 5 Zinc
1/4″ Swivel Pin	1/4–20 x 5/8″ Socket Head Cap Screw	2	0.69	1.38	Fastenal	Steel; Black Oxide Coating
M3 Lock Pin	M3 x 35 Socket Head Cap Screw	2	0.38	0.76	Fastenal	Steel; Black Oxide Coating

Cost may vary depending on shipping fees and tax. Tax not included.

*The thermocouple sensor purchased here included a prototyping amp that was not used in this build. Any alternative ***ungrounded*** high temperature K-type thermocouple would be sufficient and could easily be adapted with a different mounting method. It is critical the thermocouple is ungrounded for this application.

**Table 5:** Cold and Hot Scientific Sheet Press 3-D Printing Bill of Materials.

**Component Designator**	**Quantity**	**Print Time per unit**	**Mass per unit (g)**	Cost per unit CAD*	**Total Print Time**	**Total Mass**	**Total cost** CAD*	**Material type**
Electrical Enclosure Body	1	22 h 5 *m*	247.3	7.17	22 h 5 *m*	247.30	7.17	PETG
Electrical Enclosure Lid	1	9 h 29 *m*	113.38	3.29	9 h 29 *m*	113.38	3.29	PETG
Solid Electrical Enclosure Lid	1	13 h 15 *m*	150.53	4.36	13 h 15 *m*	150.53	4.36	PETG
Electrical Enclosure Rear Cover	1	4 h 48 *m*	77.96	2.26	4 h 48 *m*	77.96	2.26	PETG or HDPE
Electrical Enclosure Mounting Bracket	2	1 h 21 *m*	12.56	0.36	6 h 41 m**	64.96	1.88	PETG
Electrical Enclosure Mount	2	1 h 44 *m*	17.55	0.51				PETG
PSU Bracket	1	18 *m*	3.03	0.09				PETG
Nano Bracket	1	20 *m*	3.16	0.09				PETG
Total with 3-D Printing Only***						**540.75**	**15.67**	
Total with optional HDPE Sheets***						**425.64**	**12.34**	

* Based on a 1 kg spool at 28.99CAD (0.02899CAD/gram) with all printed at 0.20 mm Quality settings.

Note: To ensure print repeatability and quality comparable to that produced in this build, see Table 9 in Section 5.4.1 for details.

** All Electrical Enclosure Mounting Brackets, Electrical Enclosure Mounts, Nano Bracket, and PSU Bracket were printed simultaneously requiring the print quantity to be (1) despite multiple prints being achieved.

*** The Electrical Enclosure can be either solely 3-D printed using the **Solid** Electrical Enclosure Lid and Electrical Enclosure Rear cover, or the system can be printed using the Electrical Enclosure Lid and supplemented with 16-gauge HDPE sheets forms in the sheet press during the preliminary trials using recycled plastic to form a rear cover and an artistic front cover that can be slotted into the lid.

## 5 Build Instructions

Due to the well-established nature of compression molding as a forming method of thermoplastics, this hardware can be simplified into three major system features: a system for applying a compressive load, a variable heat source, and a mold for directing the flow of plastic. The following build instructions separate the manufacturing of this hardware into a) the mechanical assembly which provides the overall structure of the hardware and is responsible for housing the heaters and providing compression, b) an electrical assembly which offers the ability to control the variable heat source, and c) example molds that were used for basic validation and testing. The build instructions navigate the user through the assembly, leading with the mechanical frame, followed by the electronics, and finally the integration of the subsystems.

This section assumes all laser cut parts have already been ordered and acquired using the DXF files accessible in the OSF. The build instructions will review how to weld the sheet press plate subassemblies, weld the supporting frame, cut and bend the Sheet Metal Enclosures, 3-D print electronics housings, and assemble all electronics in-house. For components such as the Mounting Brackets, material was ordered as cut-to-length stock and any features including holes and slots were added later with a drill press. Both procedures can alternatively be outsourced for an additional fee. Ensure the following tools are available before beginning. Note that due to the nature of this assembly adhering to SAE standards for stock metal, the mechanical system uses imperial units for all fasteners and stock, while the electrical system uses metric fasteners and design to correspond to metric hardware.

• Measuring tape and ruler
• Masking tape
• Scribe
• Carpenter square
• Angle grinder and coarse finish grinder flap disc
• File/rasp set
• Acetone
• Rags
• Steel Wire Brush
• 180–220 grit sandpaper
• Mallet
• 4X 8″ C-clamps (minimum)
• Mini Quick-grip 6″ bar clamps
• Portable welder (Metal Inert Gas (MIG) with argon shielding gas used here)
• Nozzle dip gel
• Coolant
• Drill press (and/or hand drill)
• Horizontal band saw
• Permanent marker
• Round punch (for setting and starting holes)
• 1/4″ drill bit
• 1/2″ counter sink
• 7/16″ wrench
• 3/16″ Allen key
• 1.5 mm Allen Key
• 2.5 mm Allen Key
• 3 mm Allen Key
• Press Brake (For cutting and bending sheet metal)
  o 90-deg ½” Straight Punch (forming)
• Sheet Metal Shears
• 2″ Paint Brushes
• Stir stick
• Precision Needle-Nose Pliers
• Precision screwdriver set
• Wire strippers/cutters
• Mini side cutters
• Soldering iron and solder

### 5.1 Sheet Press Upper and Lower Plate Assemblies

Prior to beginning the assembly, please note the handling of all steel components requires the use of steel toed shoes to avoid crushing injuries. Surroundings should be assessed prior to moving the subassembly to minimize the risk of interference and stumbling while carrying heavy equipment. Due to the raw edge of laser cut parts and risk for burrs on metal, work gloves are also recommended unless using feeding equipment such as band saws, grinding wheels, or belt sanders in which case the gloves would pose a greater risk for entanglement.

#### 5.1.1 Assembling and Welding the Frame

The welded pressing assembly was designed to minimize the number of unique components needed and eliminate the need for an additional welding jig by relying on a lattice of interlocking plates that provide enough support and datums to replace an external jig. Refer to the visual BOM in Table 6 above for part references.

**Table 6:** Visual BOM of the Upper and Lower Plate Assembly Components.

**Item #**	**Component Designator**	**Rendering**
1	Center Support	
2	Interlocking Tab	
3	Jack Support L1	
4	Jack Support U1	
5	Outer Framing Wall	
6	Upper Steel Platen	
7	Lower Steel Platen	
8	Mounting Bracket	
9	Upper Sheet Metal Enclosure	
10	RH Lower Sheet Metal Enclosure	
11	LH Lower Sheet Metal Enclosure	
12	1/4″-20 x 1″ Flat Head Cap Screw	
13	1/4″ Nut	
14	1/4″-20 x 1″ Hex Head Cap Screw	
15	3/16″ Washer	

To promote modularity, the Outer Framing Walls are each notched at the ends to allow the inverted neighboring member to mate flush against a flat XY corner datum. Each includes a T-slot along the center of the length for locating the Center Supports and Jack Supports (L1 and U1). The neck of this T-slot running perpendicular to the length of the plate locates the end of each center and Jack Support, while the short slot making up the top of the “T” and parallel to the length of the plate allows the Interlocking Tab to secure these components for welding and assembly without the need for an additional bracket or fastener. The combined use of Interlocking Tabs, notched abutments, and nested Center Supports promotes ease of assembly, rigid alignment for welding without an external jig, and a sufficiently reinforced frame capable of withstanding the anticipated compressive loading during testing.

The position and shape of all slots and notches on the laser cut components are designed to intersect with the outer edge of each part to reduce the number of laser-cut punctures to a singular outer pass and minimize cost. Any position-dependent features (such as the square cutouts along the length of the Center Supports used to organize wires) are mirrored along the part to eliminate the need for two unique components. The same component can therefore be used in multiple orientations to the benefit of simplifying the assembly for the user.

**Fig. 2:**
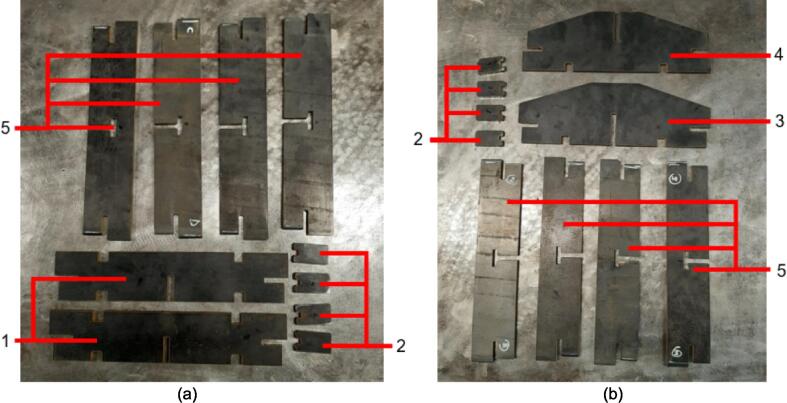
(a) Upper plate component inventory and (b) Lower plate component inventory. The necessary parts are identified as 1. Center Supports, 2. Interlocking Tabs, 3. Jack Support U1, 4. Jack Support L1, 5. Outer Framing Walls. See the visual BOM in Table 6 for confirmation.

Assembly must begin with the preparation and welding of the upper and lower plate assemblies. Prior to welding the frame, the Outer Framing Walls, Center Supports, Jack Support U1, Jack Support L1, and Interlocking Tabs must be filed down, dry fit, and prepared for welding. See Fig. 2 for the components used for both welding, and welding jig set up.

1. Lightly file all (X5) interior faces of the T-slot in each Outer Framing Wall.
2. Lightly file all (X3) interior faces of the slots in the Center Supports and Jack Supports (U1 and L1).
3. File all (X4) faces running parallel to the length of the Interlocking Tabs.
4. Place the Outer Framing Wall in a vice while ensuring both ends remain accessible. Use an angle grinder and coarse flap disc to grind a roughly 30° bevel along each welding seam as shown in Fig. 3. These mating bevels promote weld penetration at each butt joint.

**Fig. 3:**
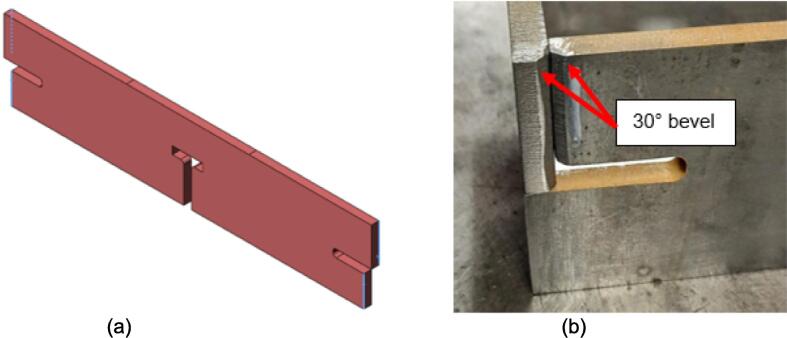
Location of bevels on each Outer Framing Wall. (a) Position of each bevel identified in blue on the end faces. (b) Example of sufficient angle and material removal. This also indicates how the beveled edges are intended to mate. (For interpretation of the references to color in this figure legend, the reader is referred to the web version of this article.)
5. Polish the beveled welding surfaces with a steel wire brush followed by a rag wet with acetone to improve the welding interface. It should be noted that this step can be omitted at the risk of a more porous weld, reduced penetration and/or additional surface splatter.

The assembly for the upper and lower plate frames are nearly identical and vary only by the presence of the flat Center Supports of the upper plate frame versus the trapezoidal Jack Supports (U1 and L1) of the lower plate frame. The following dry fit and welding instructions focus on the upper plate frame; however an identical procedure should be followed for the lower plate frame. The following assembly sequence also qualifies as the dry fit. Should any step have been unachievable due to tolerancing issues and interferences, separate the pieces and increase the amount of filing/grinding until they fit snugly together.

6. Interlock the two Center Supports using the center slot shown in Fig. 4a. With appropriate laser cutting tolerances and sufficient filing, all parts should slot together smoothly, however, if resistance occurs, use a mallet to encourage the two plates to lay flush with one another. Set this subassembly down and place the Outer Framing Walls around the Center Supports in their respective mating orientations as shown in Fig. 4b with an Interlocking Tab assigned to each T-slot position.

**Fig. 4:**
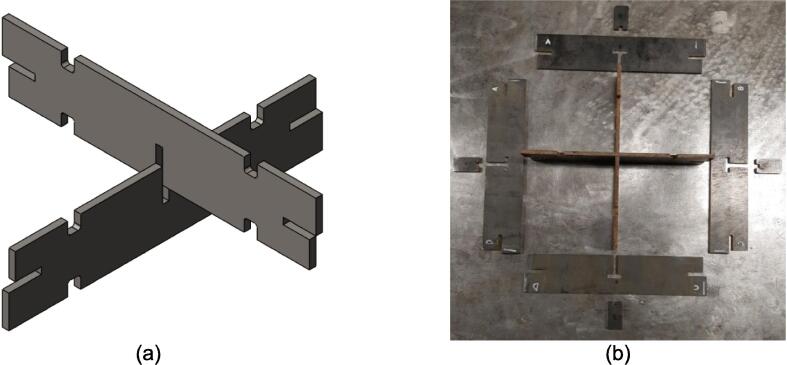
Component layout for ease of interlocking during assembly. (a) Visible representation of interlocking mechanism described. This same interlocking method is used for Jack Support U1 and L1 requiring L1 to slot into U1. (b) Flat pattern layout prior to assembly. All components can stand up from this position and interlock.
7. Tip the Outer Framing Walls up off the table until the notches of the Center Support are nested within the T-slots of the Outer Framing Walls. Slide the Interlocking Tabs into the bottom of each T-slot to lock the assembly in place as shown in Fig. 5.

**Fig. 5:**
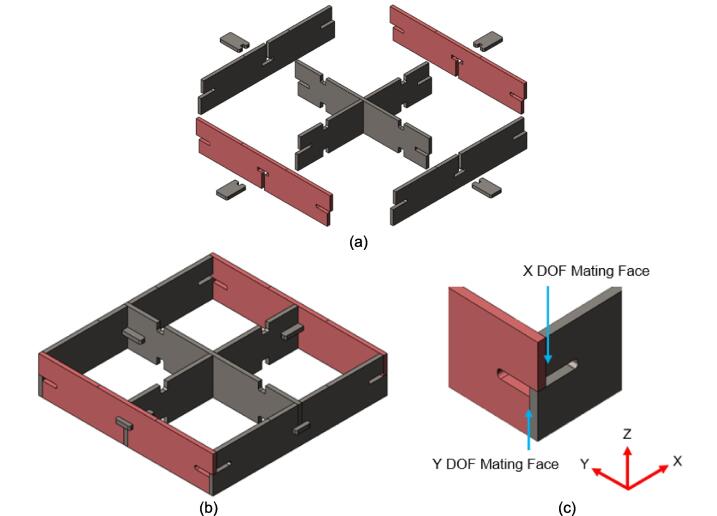
(a) Exploded view, and (b) assembled view of Outer Framing Walls dry fit position with emphasized XY locating method in (c).

Once the assembly has been dry fit, the Outer Framing Walls need to be rigidly secured in preparation of tack welding the assembly.

8. Loosely tension two pairs of 8″ C-clamps between the Center Supports and the Outer Framing Walls as shown in Fig. 6. It is critical that the clamps are biased toward the upper half of the Outer Framing Wall to maximize the amount of forced contact with the X degree of freedom (DOF) mating face.

**Fig. 6:**
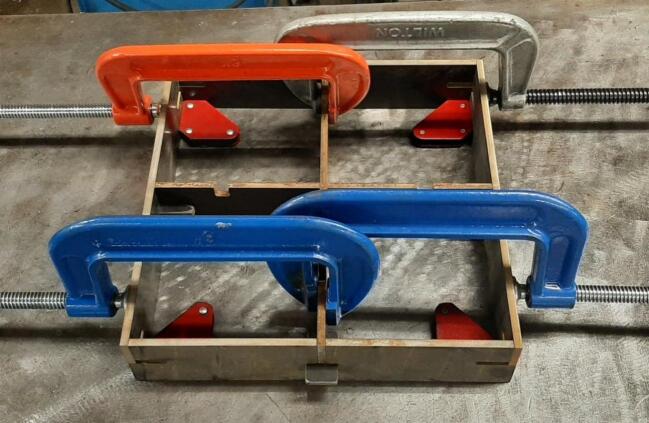
Clamping orientation for tack welding.
9. Close any gaps between the plates by lightly tapping the faces of the Outer Framing Walls with a mallet and incrementally tightening the clamps. Measure the diagonals of the box periodically and use a carpenters square to ensure the overall square is maintained and correct any misalignment. The subassembly is prepared for welding once all clamps are tightened, gaps closed, and dimensional accuracy maintained.

**Welding Safety:** Prior to welding, the welder must be grounded to a polished surface on one of the Outer Framing Walls to maximize the electrical contact. Heat resistant leather welding gloves, a leather welding jacket, leather steel toed shoes, safety glasses, and an auto darkening welding helmet must be used. Remove any flammable materials and debris from the work area and clear any unprotected parties from the area. Use welding partition walls to protect others if working in proximity is unavoidable. Note that weld settings will vary depending on the machine used. This build used the Millermatic 251 Wire MIG Welder set to 19.2 V and 314 ipm (inches per minute) wire speed.

10. Add a single tack weld to each of the beveled butt joints at the positions shown in Fig. 7. Once all corners have been secured, remove the clamps.

**Fig. 7:**
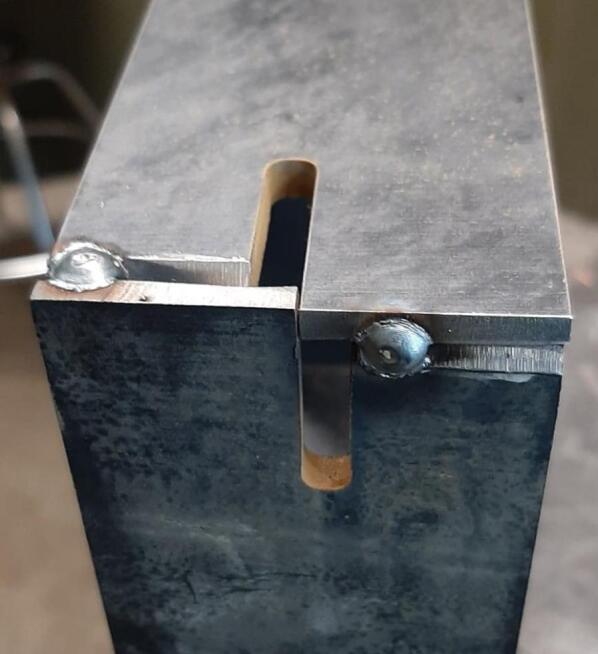
Corner butt joint tack welds.
11. For the average welder, a horizontal bead is easier to maintain. To promote operator ease after tacking, clamp the assembly on its side and add two 1″ welds along each seam.
12. Grind down any weld bead that extends beyond the edge of the Outer Framing Walls and could interfere with mounting of the Steel Platen. **This interface must be flat.**
13. Grind down the finished welds for a cleaner surface finish. This is necessary for later mounting the upper and lower Sheet Metal Enclosures.
14. Repeat steps 6 through 13 for the lower plate assembly and Jack Supports (U1 and L1)

**Fig. 8:**
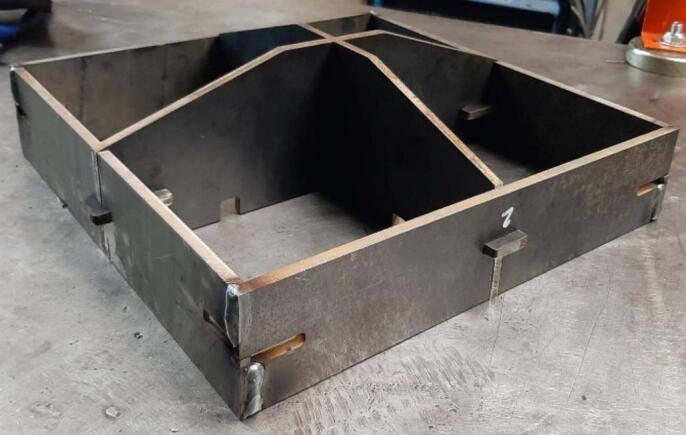
Jack Support lower Frame assembly welded and complete.

The Upper and Lower Plate Frames are complete and prepared for final assembly as seen in Fig. 8.

#### 5.1.2 Postprocessing the Mounting Brackets and Steel Platens

While the Mounting Brackets can be ordered to length (1″), they are unfinished, sharp with burrs, and still require the addition of a 1/4″ hole and slot. Similarly, the Upper and Lower Steel Platens have the appropriate mounting holes from the laser cut pattern but lack the necessary counter sink to mount flush. The following section outlines the necessary postprocessing needed to add these features. Note, these brackets could have also been ordered as a single length of angle stock and cut to length on a horizontal band saw to reduce cost.

1. File down all raw edges and burrs on the brackets.
2. Using a permanent marker and ruler to measure and mark the location of the 1/4″ hole. Repeat for the slot by marking the center point of the “holes” that make up the ends of the slot. The position for these marks can be seen in Fig. 9. Note that while the location of these marks is important, the design uses a series of holes and slots on the Mounting Brackets, Steel Platens, and Outer Framing Walls to allow for lower machining tolerances.

**Fig. 9:**
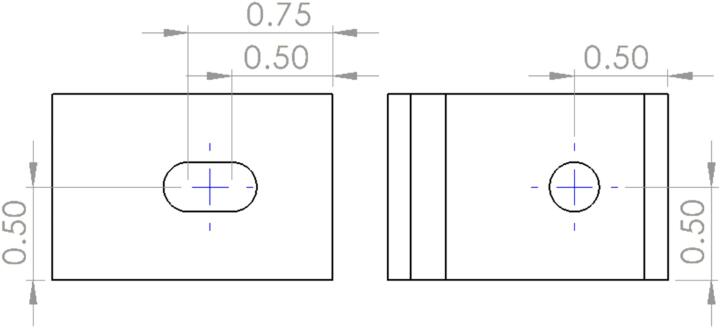
Slot and hole position defined by steel punch markers. LH view and Front View. All dimensions are in inches, and the hole and slot diameters are 1/4″.
3. Using the die punch and mallet, indent the hole center on each of the marked points to produce Fig. 10. This indentation will ensure the drill bit does not slip across the surface of the steel and improve the part accuracy.

**Fig. 10:**
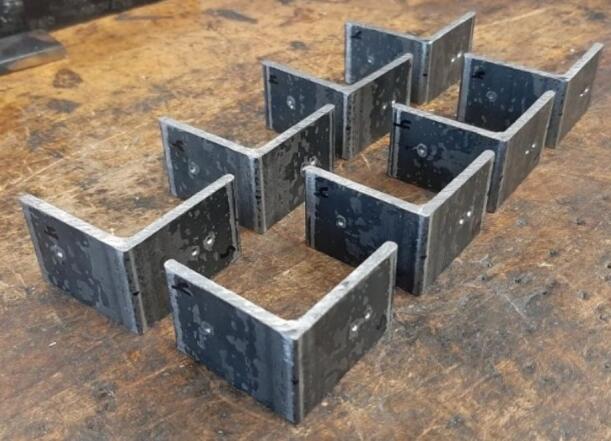
Located and punched holes and slots for Mounting Brackets.
4. Load the 1/4″ drill bit into the drill press. Secure a Mounting Bracket within a vice on the drill press table as shown in Fig. 11. Apply coolant liberally to the hole site, power on the drill, and slowly begin removing material. Be sure to periodically back off the drill and top-up the coolant for each hole to promote chip removal and prevent the bit from overheating.

**Fig. 11:**
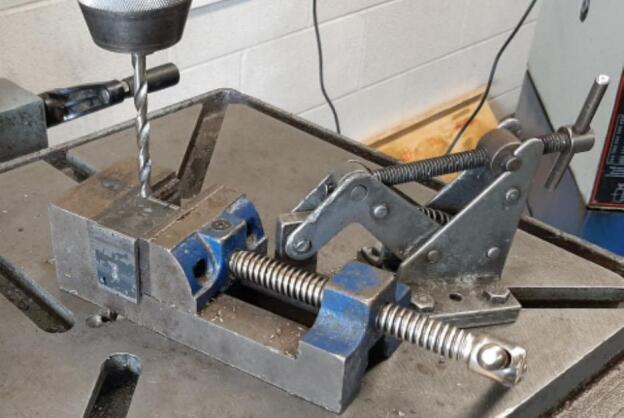
Drill press work holding method for Mounting Bracket hole and slot.
5. Repeat step 4 for all Mounting Brackets using the same work holding setup before moving onto the slot.
6. Realign the work holding setup for one extreme of the slot. Using coolant and the same process as before, drill out one side of the slot on each Mounting Bracket. Adjust the work holding setup for the other end of the slot and repeat. Once complete, use a rasp to remove the material at the center of the slot not captured by the holes and smooth out the profile.
  a. If available, use a CNC to program the slot path and complete each Mounting Bracket using the same work holding would improve this process.
7. Use a small file to remove any remaining burrs from the drilled holes and clean the coolant off with a rag.

Once the Mounting Brackets are complete, the postprocessing for the Upper and Lower Steel Platens can begin.

**Fig. 12:**
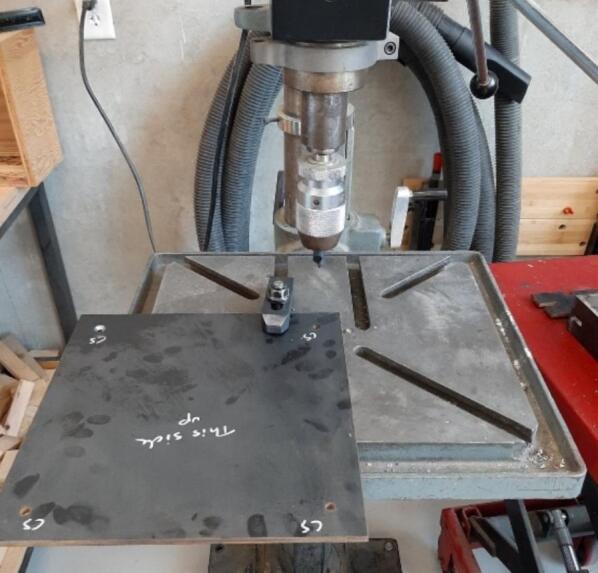
Steel Platen work holding for counter sunk mounting holes.

8. Exchange the 1/4″ drill bit for the 1/2″ counter sinking drill bit.
9. Identify the smoother, blemish-free side of the Steel Platens, and loosely clamp one to the drill press table. Align one of the 1/4″ laser cut mounting holes to the center of the drill bit. Once centered, securely clamp the Steel Platen as shown in Fig. 12.

Note that the Steel Platen included in this build does not have pre-cut mounting holes for the heating elements. For this prototype, different elements were tested, and each relied on a different mounting method. As a result, the platens were prepared without any element-specific mounting holes. The final product relied on the insulation packed in behind the elements and the compression applied by the Sheet Metal Enclosures to keep the elements used in place. While this was sufficient for the prototype, this method introduces the possibility of an inconsistent contact between the elements and the platens and therefore reduced thermal conduction and overall machine performance. The elements may warp with use to reduce contact or, in the case of the lower plate assembly, they may settle away from the Steel Platens. It is therefore advised that the hole pattern provided be used and each additional mounting hole also countersunk. Should the user opt for a different element, the hole pattern must be adjusted or, like this model, insulation must be used to hold the elements in place.

10. Apply coolant liberally and slowly begin removing material. Periodically verify the depth of the counter sunk hole by inserting a 1/4–20 x 1″ FHCS until the screw sits flush with the surface of the plate. Repeat this for all holes on each plate.

#### 5.1.3 Assembling the Sheet Press Upper and Lower Plate Subassemblies

The sheet press upper and lower plate assemblies were designed to provide forgiving tolerancing. The slots in the Mounting Bracket and Outer Framing Wall provide an additional DOF in the X and Y respectively to account for any positional errors while locating any of the previously drilled holes. The hole of the Mounting Bracket aligns with the slot of the Outer Framing Wall, while the hole of the Steel Platen aligns with the slot in the Mounting Bracket. Further, the Steel Platen must be guaranteed to sit flush against the edge of the framing assembly, so an intentional gap between the Steel Platen and the Mounting Bracket face was added.

1. Feed a 1/4–20 x 1″ FHCS through each of the corner holes on the Steel Platens before laying the platens on a flat surface with the counter sunk “working face” down.
2. Place the Upper and Lower Plate Assemblies on top of their respective Steel Platens and roughly align the edges. Any minor imperfections or warping added during welding will be accounted for by the Mounting Brackets
3. Place a Mounting Bracket at each of the 4 corners of the Steel Platens. Fit the slotted face of the Mounting Bracket over the FHCS and align the hole on the Mounting Bracket with the **lower** of the two slots at the intersection of each Outer Framing Wall. See Fig. 13 to identify the lower slot.

**Fig. 13:**
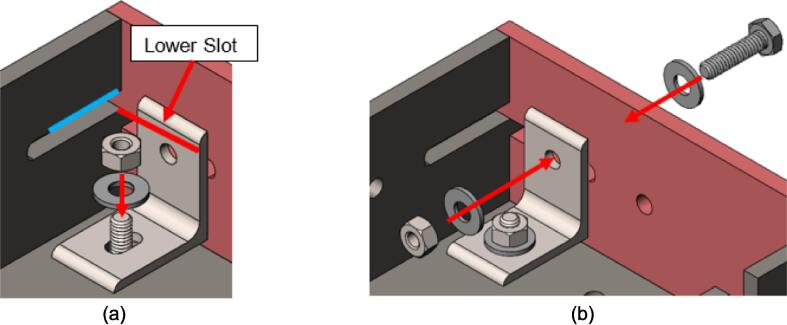
Mounting Bracket assembly and bolt positioning. (a) Indicates the lower slot in red to be used for mounting the brackets and the position of the slot on the bracket vs. the hole. (b) Once the FHCS has been placed in the bottom of the bracket the HHCS can follow as shown. (For interpretation of the references to color in this figure legend, the reader is referred to the web version of this article.)
4. Add a 3/16″ washer to each of the 1/4–20 x 1″ HHCS before sliding them through the Outer Framing Wall and the Mounting Brackets from the outside of the frame to the inside.
5. Add a 3/16″ washer to the back of each bolt before loosely threading a 1/4″ nut onto each of the four FHCS and SHCS.
6. Tighten the nuts on the FHCS in a diagonal-pair sequence, while monitoring and adjusting the alignment of the framing assembly relative to the edge of the Steel Platen as necessary. For the final turns, use a 3/16″ Allen key to hold the FHCS in place from the bottom, and a 7/16″ wrench to tension the nut.
7. Repeat the same diagonal-pair tensioning sequence on the HHCS to secure the Steel Platen to the framing assembly.
8. Once the Mounting Brackets are secured, refer to Fig. 14 below to locate the position for placing a 1/4″-20 x 1″ FHCS on the surface of each Steel Platen. Coat the threads of each bolt in nozzle dip gel, or an otherwise anti-spatter surface coating, and weld each bolt in place. These bolts will provide a mounting location later for the securing the protective earth (PE) ring terminal, so the quadrant and position relative to the Mounting Brackets is critical for ensuring the wires neatly exit from the appropriate side.

**Fig. 14:**
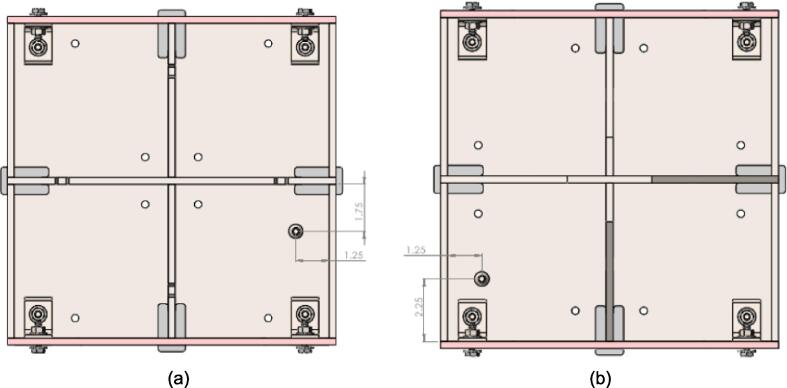
Final (a) Upper plate assembly and (b) lower plate assembly with PE bolt locations identified. Note the hole patterns between the upper and lower plate assemblies are unique and must be correctly assigned.

While the above assembly detail describes the base model for the upper plate assembly, two additional configurations are easy to switch between and can be used from the start depending on the application of interest. The reinforced base configuration is designed to comfortably support 45.5 PSI (pounds per square inch) of force and was validated through Finite Element Analysis (FEA, see section 7.1). With 45.5 PSI acting as the maximum load case the sheet press will experience, the actual required load will be less depending on the specific application, thickness, and plastic of choice. Therefore, these additional configurations provide a potential reduction in material and cost for reduced loading cases. To complement these configurations, a large benefit of using Interlocking Tabs to mate the Center Supports and the Outer Framing Walls is the quick release effect they provide. These tabs can easily be removed to release either one or both Center Supports for a quick switch between configurations.

**Fig. 15:**
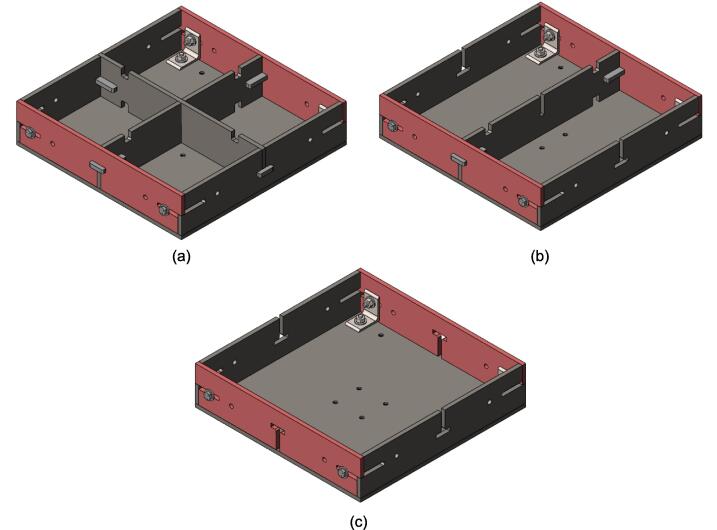
(a) Fully reinforced upper plate assembly configuration 1 with two Center Supports; (b) Configuration 2 with singular Center Support; (c) Configuration 3 with no Center Supports.

Therefore, the most reinforced configuration described above can be used for either high loading cases or to forgo the fabrication of the standalone support frame and use the sheet press frames with an existing hydraulic press. If the product tests demonstrate that the desired effect can be achieved using a reduced load, and the worktable is large enough to support the frame edge or the standalone machine is built, then the Center Supports can be removed. Between configuration 1 and 3 shown in Fig. 15, the design can be configured to eliminate 6 components.

#### 5.1.4 Manufacturing the Sheet Metal Enclosures

To pack the insulation around the heating elements a Sheet Metal Enclosure was added to both the upper and lower frames. This housing utilizes the existing fasteners on the frames and requires only simple fabrication methods. Note that both the upper and lower enclosures can be laser cut to simplify the process but were done by hand using punches, sheet metal hand shears, a pressing brake, and a sheet metal shear table to reduce cost. For the purposes of these build instructions, it is assumed a laser cut part was ordered.

The Sheet Metal Enclosure will be mounted after the complete press has been assembled and the heaters have been installed. See section 5.3.3. and 5.3.4. for details.

**Fig. 16:**
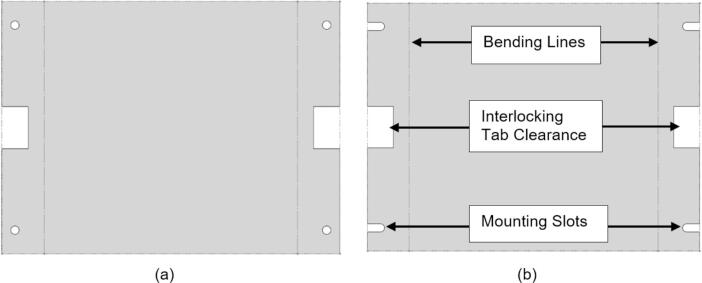
Upper Sheet Metal Enclosure preparation of mounting holes, and clearance. Progression from clearance holes in a) to Slotted tabs in b).

A) Upper Sheet Metal Enclosure
1. Using the provided DXF file, order 1 laser cut Upper Sheet Metal Enclosure flat pattern. Use a scribe and a square to mark the two bending lines as shown in Fig. 16 to be 2″ from the edge of the enclosure along its length.

To reduce cost and avoid use of laser cutting, the enclosure can be fabricated by using a pressing brake to cut the 16″ x 12″ rectangle from sheet metal stock. Once cut, the holes for the mounting slots and Interlocking Tab clearances can be measured and marked. The slots can be started using a 3/8″ die punch, before being finished using sheet metal shears to cut tangent to the hole and perpendicular to the edge of the enclosure.

**Fig. 17:**
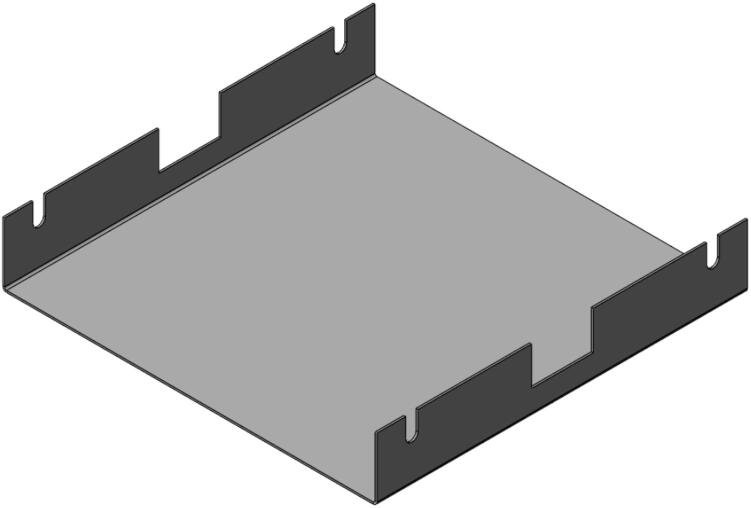
Complete Upper Sheet Metal Enclosure.
B) Lower Sheet Metal Enclosure

2. With a pressing brake and 1/2″ Straight punch, align one bending line with the punch at a time and bend the flanges up to 90 ${\;}^{{}^{\circ}}$. The final enclosure is shown in Fig. 17.

The lower Sheet Metal Enclosure consists of four separate parts, each used to shield one quadrant of the lower plate assembly. While the flat pattern for these parts is the same, two-part configurations are required to cover the mirrored halves of the assembly.

1. Use the provided DXF file for the Lower Sheet Metal Enclosure Flat Pattern to order 4 parts.
2. Using a scribe and a square, mark one bending line 1 5/16″ form the angled edge and the second bending line 2″ from the opposite straight edge of the enclosure as shown in Fig. 18.

**Fig. 18:**
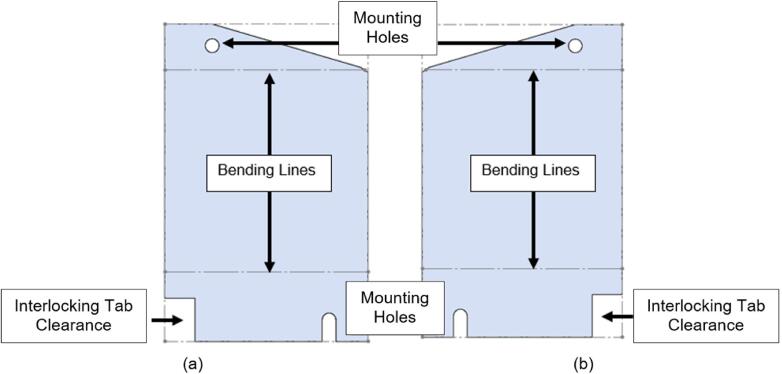
Lower Sheet Metal Enclosure preparation of mounting holes, and clearance. (a) RH flat pattern, (b) LH flat pattern.
3. For two of the flat patterns, bend the angled edge upwards along the bending line, followed by the opposite end downward. For the remaining two flat patterns, bend the ends in the opposite directions relative to the first parts. The result will be two LH and RH lower Sheet Metal Enclosures as shown in Fig. 19.
  a. Note that like the upper Sheet Metal Enclosure, the flat pattern can be first scribed and cut using a pressing brake or sheet metal hand shears to reduce costs and eliminate the need for laser cutting. This approach was taken for this build, however laser cutting will yield a cleaner product.

**Fig. 19:**
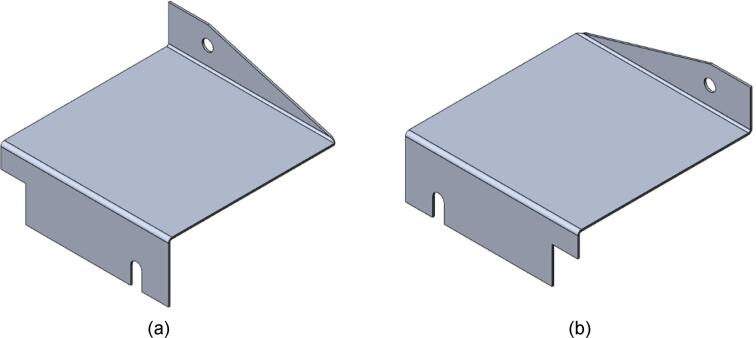
Lower Sheet Metal Enclosure. (a) RH enclosure X2, (b) LH enclosure X2.

If a hydraulic press is accessible and only the pressing plates are of interest separate from the supporting frame, the build instructions can skip to section 5.3 to begin installation of the heaters and electronics. If a standalone machine is desired, the build instructions for the supporting frame in the following section are necessary.

### 5.2 Supporting Frame

The hot press was designed with the option to be used either as two free upper and lower plate assemblies in conjunction with an existing hydraulic press should one be available to the user, or as a free-standing machine converted by adding a supporting frame and 4-ton bottle jack. The following section details how to prepare and build this supporting frame using the components detailed in Table 7.

**Table 7:** Visual BOM Sheet press Supporting Frame.

Item #	Component Designator	Rendering
1	Assembly Base Plate	
2	Support Leg	
3	Electrical Enclosure Support Leg	
4	Lower Plate Support	
5	Linear Guide	
6	Jack Spacer	
7	Jack Locating Guide	
8	1/4″ Nut	
9	1/4″-20 x 1″ Hex Head Cap Screw	
10	1/4″-20 x 1 3/4″ Socket Head Cap Screw	
11	3/16″ Washer	

#### 5.2.1 Material Preparation

All parts can be either ordered to size or cut from larger stock. The following steps must be taken to achieve the dimensioned stock required for assembly.

1. Order a 16 1/4″ x 13 1/4″ x 1/4″ thick mild steel plate for the assembly base plate.
2. The Support Legs were cut from 8ft scrap tubing into four 19″ and 1″ lengths. If ordering stock either standard lengths can be ordered and cut to size or, for simplicity, 20″ length square tube can be ordered and 1″ lengths cut off the end of each of the 4 tubes using a horizontal band saw. This excess 1″ of material can then be used as the lower plate supports while the Support Legs would use the remaining 19″ length.
3. The Linear Guides were ordered to length, though similarly can be cut from a longer piece of stock into four 2″ lengths.
4. A 4″ x 3″ x 3/16″ thick mild steel plate was ordered and two 1/2″ wide sections cut off the length using a horizontal band saw. These act as the Jack Locating Guides and leave a 3″ x 3″ remainder for the Jack Spacer.
5. File and deburr all sharp edges on the cut faces.
6. Measure and mark the center point of the mounting holes on the four linear guides and support legs as shown in Fig. 20a and Fig. 20b. Fix each Support Leg and Linear Guide in a drill press and drill one 1/4″ hole into each as previously described in section 5.1.2.

**Fig. 20:**
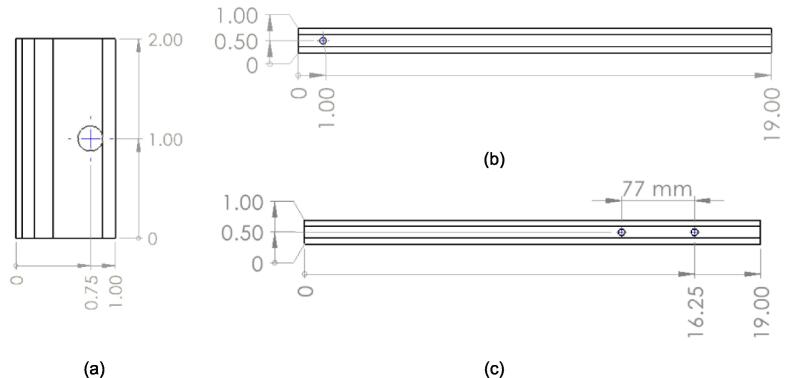
Drawings outlining mounting hole placement for supporting frame (a) Linear Guides, (b) Support Legs, and (c) electrical enclosure Support Leg. All dimensions in inches unless otherwise specified and all hole diameters are 1/4″ clearance.
7. On **one** of the Support Legs, additionally mark and drill the two holes identified in Fig. 20c for mounting the Electrical Enclosure. These holes are located at the opposite end of the Support Leg relative to the holes drilled in the previous step and on the adjacent face. Note that the separation distance between these holes is metric as the 3-D printed Electrical Enclosure utilizes all metric components.

At this point all parts for assembling the supporting frame are prepared for welding and assembly.

#### 5.2.2 Preassembly of the Supporting Frame for Welding

**Fig. 21:**
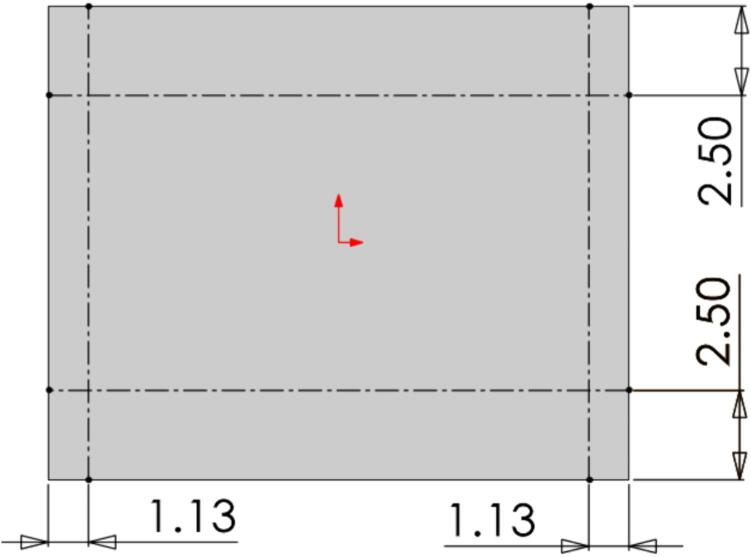
Drawing of Assembly Base Plate with scribed Support Leg locations marked. The intersection in each corner of the dimensioned lines must be lined up with the outside edge of each supporting leg. All dimensions are in inches.

1. Using a tape measure, scribe, and carpenter square, measure and mark the location of each of the Support Legs on the Assembly Base Plate. This includes an “L” marking the outside edges of each Support Leg as shown in Fig. 21.

Next the upper plate assembly must be partially assembled with the supporting legs to act as a welding jig for squaring up the legs of the support frame.

2. Align the mounting hole on each of the Support Legs with a hole on the upper plate assembly. The Support Leg length must be biased toward the pressing plate as shown in Fig. 22a. Note that the electrical enclosure Support Leg must be mounted to the quadrant of the box housing the PE bolt previously welded to the Upper Steel Platen as shown in Fig. 22b. This will act as the front-right side of the press when operating and must be welded in this orientation.

**Fig. 22:**
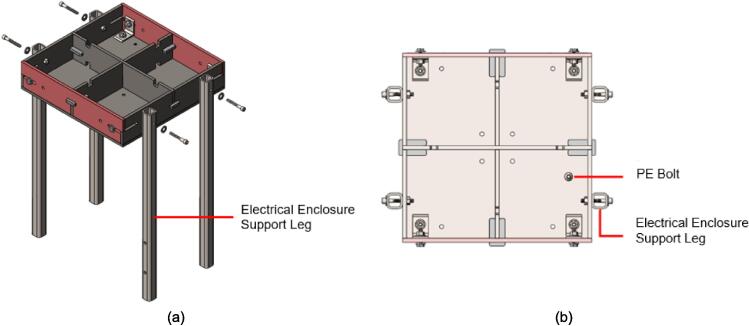
(a) Alignment and assembly of the Support Legs onto the upper plate frame, (b) Completed mounting and correct layout of electrical enclosure Support Leg relative to upper plate assembly PE bolt quadrant.
3. Feed a 3/16″ washer onto four 1/4–20 x 1 3/4″ SHCS and feed one through each of the holes on the Support Legs and upper plate frame. Feed a 1/4″ nut onto the end of each bolt inside the frame and finger tighten in place. Repeat this for each leg. It is easiest to complete this step when the upper plate assembly is flipped “top down” on the table with the Steel Platen and support legs extending up.
4. Flip the partially assembled frame upright and align each of the legs with the previously marked corners on the Assembly Base Plate. Once roughly aligned, double check the interior distance between the legs as 5 3/4″ and 12″ as shown in Fig. 23. Use a carpenter square to ensure each of the legs is perpendicular to the Assembly Base Plate. Once each of the legs have been double checked, secure them using a corner magnet to the Assembly Base Plate.
  a. Note that the 12″ separation distance must be **at least** 12″ or slightly greater to ensure sufficient clearance for the lower plate assembly to slide freely. To assist in this, an additional washer can be placed between the upper plate assembly and each supporting leg to guarantee this clearance.

**Fig. 23:**
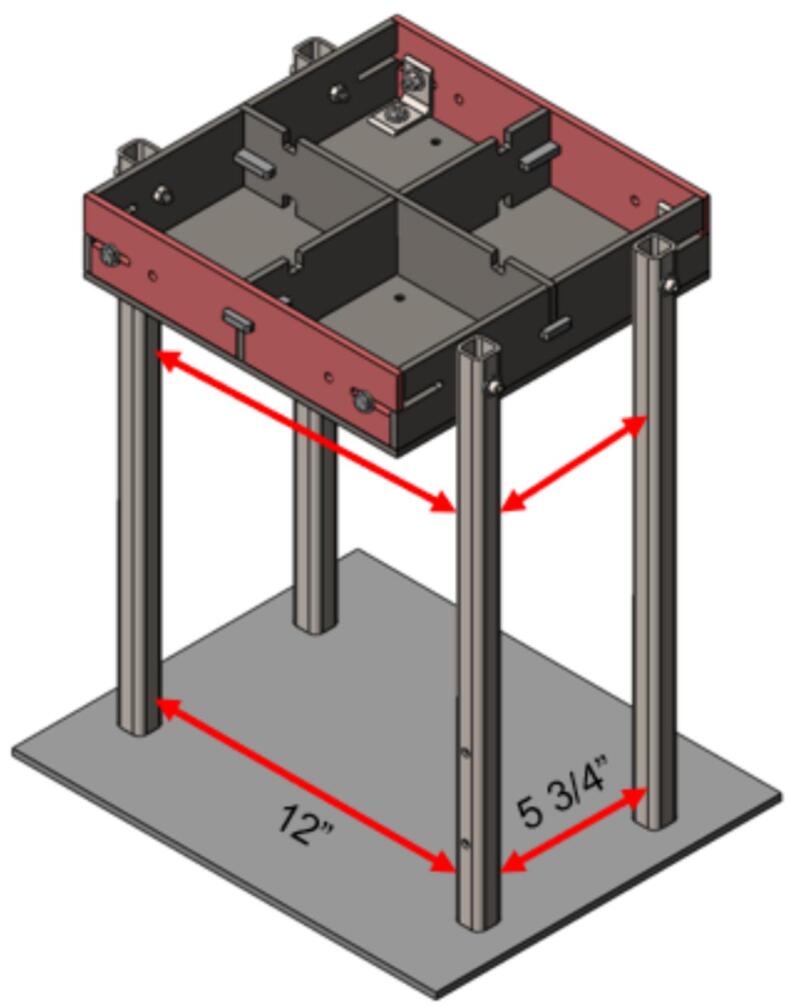
Welding jig assembly and critical dimensions.
5. Tack weld each of the Support Legs in place using a MIG welder. This build used the Millermatic 251 Wire MIG Welder set to 18.5 V and 312 ipm wire speed.
6. Once each leg has been tacked, complete a second finishing pass weld around the entire perimeter of each of the Support Legs.
7. Additionally tack each of the nuts securing the Support Legs inside the upper plate frame with two to three tack welds. The exposed bolts can be coated in nozzle dip gel to prevent spatter from damaging the threads. This allows the upper plate assembly to be installed without removing the upper Sheet Metal Enclosure in later steps.
8. Once welded, the temporary jig is no longer needed. Unscrew the 1/4–20 x 1 3/4″ SHCS and remove the upper plate assembly.
9. Measure and mark a line along each of the Support Legs 10″ down from the top of the leg. Using a corner magnet, secure a Lower Plate Support to the **outside edge** of each Support Leg as shown in Fig. 24. The top edge of each lower plate support should be in line with the previously scribed line.

**Fig. 24:**
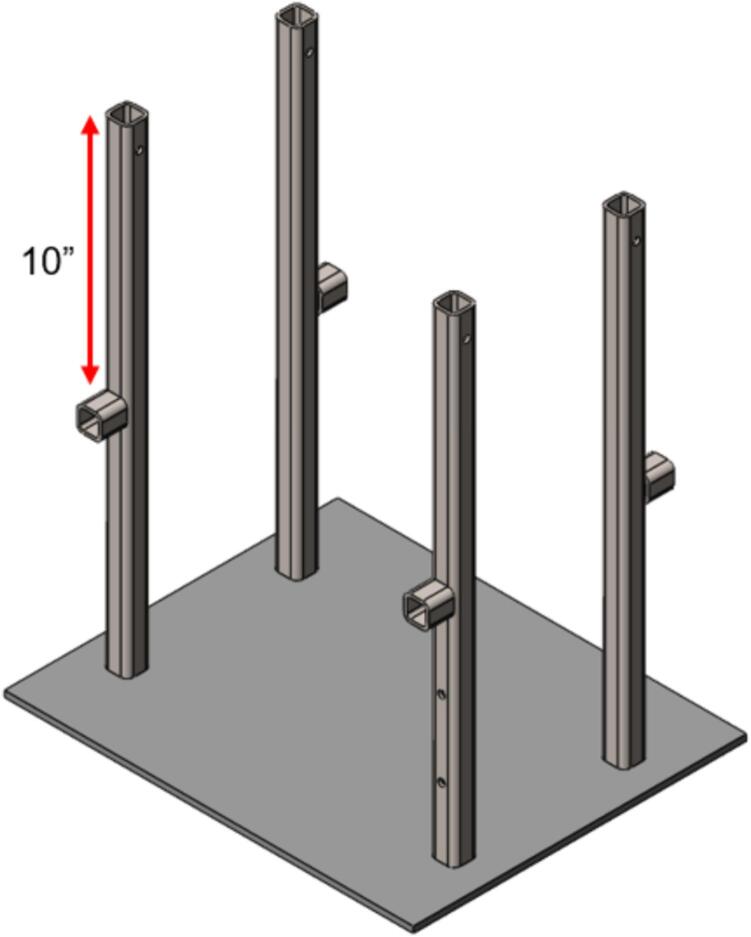
Welded Support Assembly.
10. Add a weld bead along each edge of the lower plate supports excluding the top edge.

To align the bottle jack, the remainder of the assembly must still be assembled and tuned to actuate smoothly with the lower plate assembly. Once the location of the bottle jack is dialed in, the jack locating guides can be welded in place.

11. For the lower plate assembly, align a Linear Guide with each of the slots on the faces of the frame adjacent to the Mounting Brackets (i.e., the framing walls that do NOT mount to the Sheet Metal Enclosure). This alignment can be seen in Fig. 25.

**Fig. 25:**
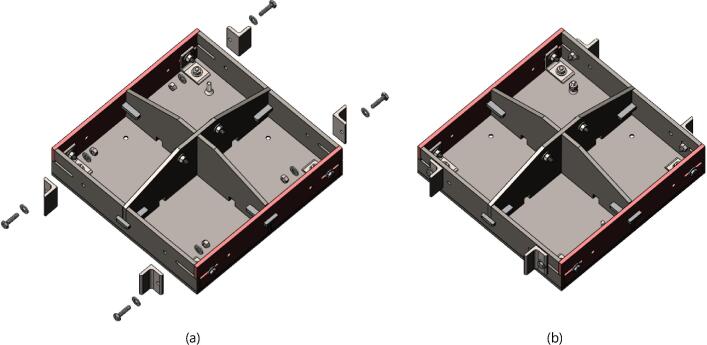
(a) Alignment and (b) assembly of the lower plate and Linear Guides.
12. Feed a 1/4–20 x 1″ HHCS through a 3/16″ Washer and each Linear Guide and into the frame. Hand tighten a 3/16″ washer and 1/4″ nut onto the inside of the lower plate frame for each Linear Guide.
13. Lift and slide the lower plate subassembly onto the welded support assembly until the Linear Guides are resting on the lower plate supports as shown in Fig. 26. The Linear Guides will slide on the outside edge of the 5 3/4″ separated Support Legs.

**Fig. 26:**
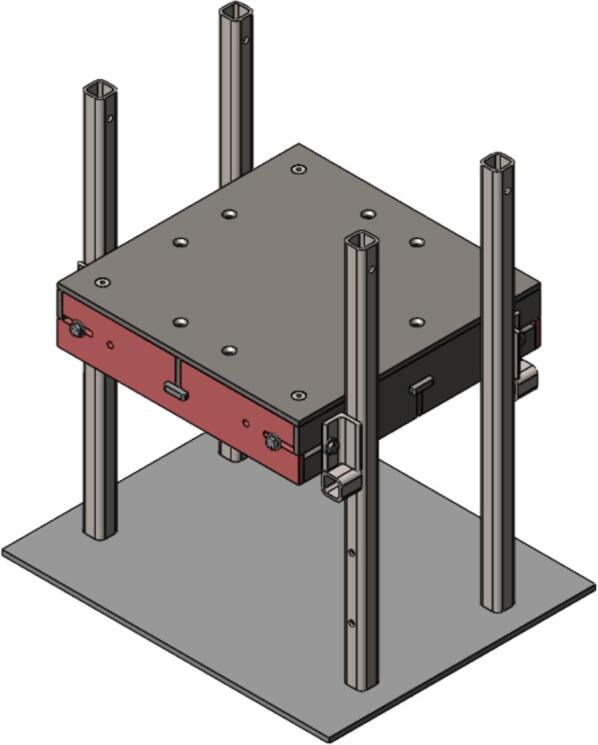
Lower plate and support frame assembly.
14. Place the bottle jack below the lower plate and align the jack’s saddle at the center of the lower plate so the lever remains accessible from the length biased side of the Assembly Base Plate closest to the Electrical Enclosure Support Leg. Square up the edges of the jack to the edges of the Assembly Base Plate and position the jack locating guides along two adjacent edges of the jack as shown in Fig. 27. Tack weld these in place to make locating the jack consistent and efficient in the future.

**Fig. 27:**
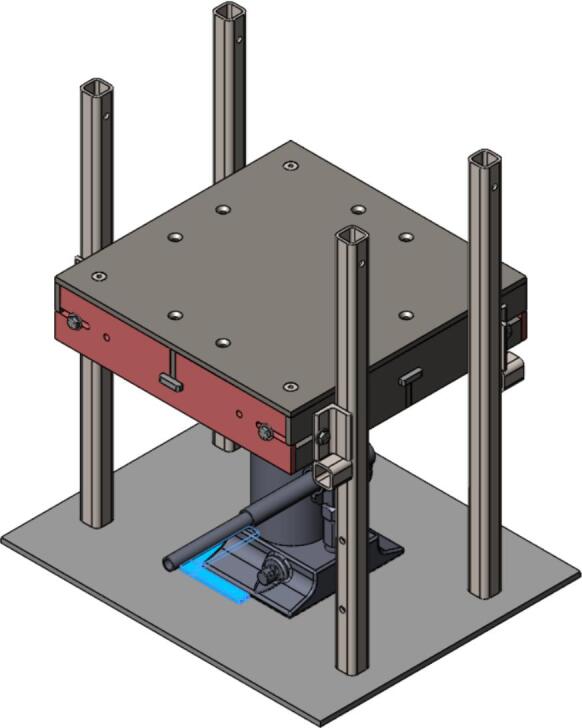
Jack locating guides alignment and welding position. Parts of interest highlighted in blue. (For interpretation of the references to color in this figure legend, the reader is referred to the web version of this article.)

**Fig. 28:**
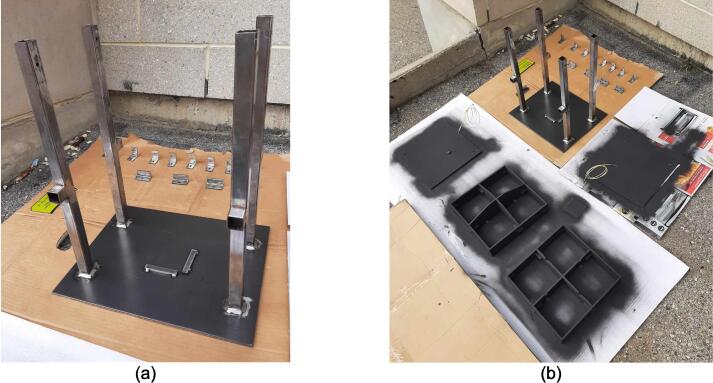
(a) completed welded supporting frame before painting, (b) painting of the components in preparation for reassembly. The upper and lower Sheet Metal Enclosures using fire red gloss are not shown here. (For interpretation of the references to color in this figure legend, the reader is referred to the web version of this article.)

At this point in the build, all structural features have been machined and welding completed. Before completing the assembly, the system should be fully disassembled, and each mild steel component spray painted with an ultra-high heat Rust-oleum to avoid future rusting of the system as shown in Fig. 28. Before painting, use masking tape to cover any exposed threads on the PE bolts and welded nuts. For the upper and lower Sheet Metal Enclosures, the inside faces of each were sprayed with Rust-oleum, while the outside faces were brushed with Armor Coat Fire Red Gloss Rust Paint. Once dry, reassemble the upper and lower plate assemblies, but do not remount them to the supporting frame yet.

### 5.3 Reassembly and Mechatronic Integration

#### 5.3.1 Installation of the Heating Elements

Once the Upper and Lower Plate Assemblies have been rebuilt, the Flat Heating Elements, wiring, ungrounded K-type thermocouples, and insulation can be added.

1. Feed a 1/4–20 x 1″ FHCS up through each of the countersunk mounting holes on the upper and lower Steel Platens.
  a. Recall that in this build, fasteners were omitted, and the elements were secured using the force of packed insulation secured with the Sheet Metal Enclosures.
2. Place a flat heating element into each of the quadrants of the Upper Plate Assembly such that their mounting slots fit over the FHCS’s. Feed a 3/16″ washer over each screw before tightening each element down with a 1/4″ nut. The element configuration for the upper plate assembly is shown in Fig. 29a. Note the location of the PE bolt relative to the Mounting Brackets.

**Fig. 29:**
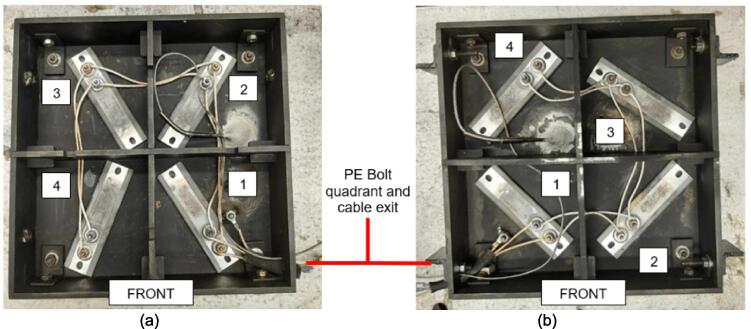
Internal parallel wiring layout for each assembly. (a) Indicates upper plate assembly, and (b) shows position comparison of lower plate assembly heaters to promote more uniform heating. The quadrants as they are referenced in the build instructions are numerically identified.
3. Repeat step 2 with the heaters and mounting holes of the Lower Plate Assembly.
  a. To reduce the time to temperature for the sheet press and to more evenly heat the shared surface, the heaters of the lower plate were rotationally offset from the upper plate assembly. This yielded an “X” configuration for the heaters on the upper plate assembly and a square on the lower plate assembly after each heater was rotated approximately 90° relative to its partner heater on the opposite plate. This configuration is shown in Fig. 29b.

Once the heaters have been positioned and secured appropriately, they must be wired in parallel using thermally insulated glass fiber wire.

**Fig. 30:**
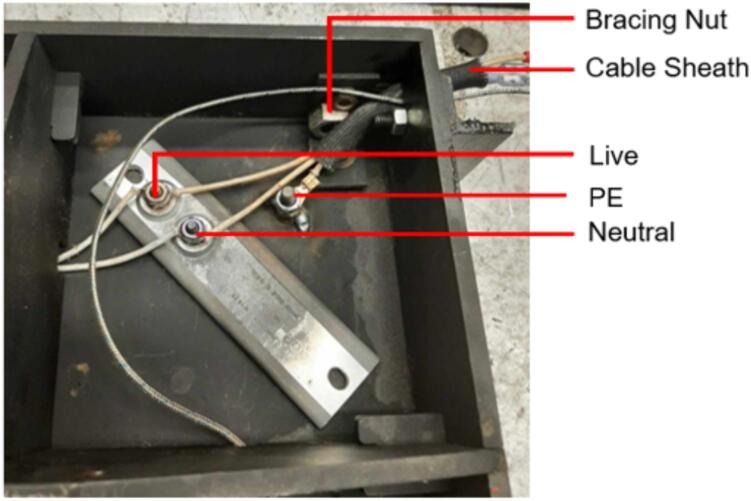
Appropriate position and labeling of wires exiting the lower plate assembly. Organizational cable sheath identified, and optional nut braced beneath the Mounting Bracket nut to prevent rotation when removing or mounting the lower Sheet Metal Enclosure mounting and removal.

1. Loosen the nut on each screw terminal.
2. Cut a roughly 150 mm length of 18-gauge high temperature hook-up wire and strip 10 mm of insulation from both ends. Crimp a female blade terminal to one end and feed the other **through the slot adjacent to the Mounting Bracket of the front right quadrant (1) housing the PE bolt in the upper plate assembly.** These female blade terminals will provide the connection to the electrical enclosure in later steps.
  a. Refer to section 5.4.2 F) for details on appropriate wire preparation techniques.
3. Wind the stripped wire around a screw terminal on the first heater. See the bottom right of Fig. 29a for the wire entrance of the upper plate assembly.
4. Measure and cut a segment of wire that spans between the screw terminal just used, and a second screw terminal in a neighboring quadrant (2). Strip both ends and lay the wire between the quadrants of the plate assembly by feeding it through the laser cut hole at the bottom of the Center Support. Secure one end of the wire to the first screw terminal alongside the first wire, and the other end to the heater in the next quadrant. Once two wires are wound around the same screw terminal, fully tighten the nut with a wrench.
5. Repeat step 3 until all four heaters have been connected to one screw terminal, then wire the reverse process back through the same holes in the Center Supports using the second screw terminal on each heater. For the last section of wire that exits the press, crimp another female blade terminal to the exiting end as in step 2.
6. Crimp a ring terminal to the end of a roughly 6″ length of wire and feed it through the same slot as the previous wires. Thread a 1/4″ nut onto the PE bolt in quadrant 1 before attaching the ring terminal. Secure the ring terminal with a second 1/4″ nut. Crimp a female blade terminal onto the opposite end.
7. Repeat this process for the Lower Plate Assembly until all elements are connected in parallel, three wires exit from the front left of the assembly as shown in Fig. 29, quadrant 1, and each wire is crimped to a female blade terminal. Note that the wires of the upper and lower plate assemblies exit on opposite sides when viewed from above because the lower plate assembly will be flipped over when mounted allowing the wires to exit closest to the electrical enclosure Support Leg on both assemblies.
8. Label each wire exiting the upper and lower plate assemblies from the elements as live or neutral, and PE form the PE bolt.
9. Cut a 3–4″ length of cable sheathing and feed the wires exiting the upper and lower plate assemblies through it as shown in Fig. 30.

#### 5.3.2 Installation of the Ungrounded Type-K Thermocouples

The hot press utilizes two ungrounded armored K-type thermocouples rated up to 800 °C with a wire length of 1.5 m and probe length of 50 mm (Φ4mm diameter) to independently monitor the temperature of the upper and lower plates. One thermocouple is dedicated to the upper plate assembly and is mounted in quadrant 2 (Fig. 29a) toward the outside of the plate. The remaining thermocouple is mounted on the lower plate assembly and is mounted in quadrant 4 (Fig. 29b) toward the inside of the plate. This positioning is necessary for gathering an understanding of the temperature across the plate from the better insulated center to the outside edge with greater heat loss.

1. For the upper plate assembly, feed the prongs of the 1.5 m thermocouple lead wire from quadrant 2 to 1 through the laser cut hole at the bottom of the Center Support against the Steel Platen and out the slot of the Outer Framing Wall in quadrant 1 alongside the cable sheath from the heating element installation. The probe should now occupy quadrant 2.
2. For the lower plate assembly, feed the thermocouple lead wire from quadrant 4 to 1 and out of the Outer Framing Wall in the same manner as step 1. The probe should now occupy quadrant 4.
3. To secure the probe, clamp the body of the probe against one of the Center Supports using a scrap block approximately 1″ thick to separate the probe from the wall as shown in Fig. 31a.
  a. Refer to Fig. 29a for probe orientation and position within their respective quadrants.

**Fig. 31:**
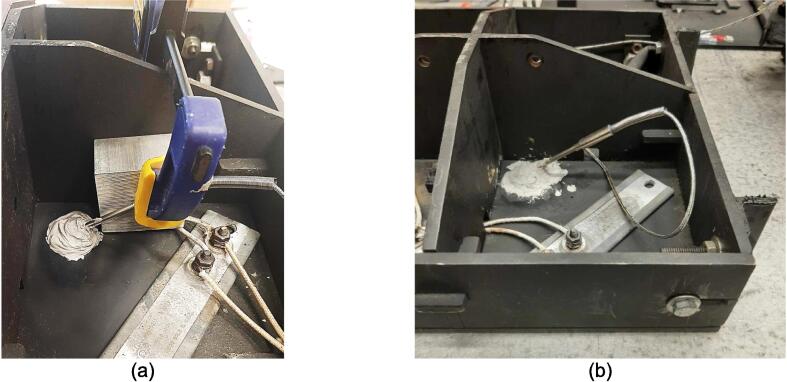
(a) Clamping method for adhering the thermocouple probe to the Steel Platen surface, (b) fully hardened and installed thermocouple.
4. Ensure the tip of the probe is stable and pressed against the surface of the Steel Platen before applying a liberal amount of J-B Weld Muffler Cement around the probe to secure it in place. Allow 24 h for the cement to dry before checking if the surface is fully hardened. Once hard remove the clamp and block as shown in Fig. 31b.

Once the adhesive has hardened, each quadrant can be packed with insulation. Note that if the elements were not bolted down as in this build, overpacking the insulation is critical for supporting the elements in place. Be sure to overfill the press with insulation before fitting the enclosure on.

5. Cut and pack approximately 6″ x 6″ squares of Rockwool Insulation into each quadrant 3 and 4 of the upper plate assembly, and quadrant 2 and 3 of the lower plate assembly until the top edge sits slightly above the Outer Framing Walls. These quadrants do not interact with the thermocouples and do not require extra attention.
6. For any quadrant occupied by thermocouple lead wire, a layer of insulation must first be installed between the lead wire and the heating elements to shield the wire from the live terminals as shown in Fig. 32a. This is critical in ensuring no shorts occur within the plate assemblies.

**Fig. 32:**
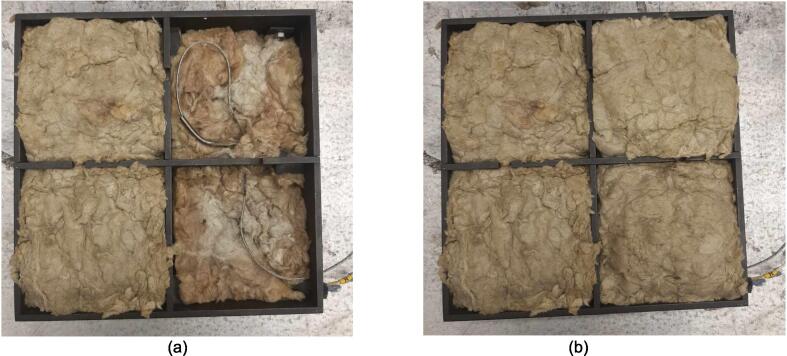
Insulation installment and thermocouple braided wire segregation for the upper plate assembly. (a) Intermediate packing step to isolate the thermocouple wire, (b) fully prepared assembly.
7. Once isolated, additional layers of insulation can be added on top to complete the packing as shown in Fig. 32b.

#### 5.3.3 Mounting the Sheet Metal Enclosures

Once the Flat Heating Elements and thermocouples have been wired and installed, the Sheet Metal Enclosures can be installed. Both plate frame assemblies should be filled with insulation and only have the cable sheath containing the live, neutral, and PE wires, and the thermocouple lead exiting from what will become the front right-hand side of the sheet press.

A) Upper Sheet Metal Enclosure
  1. Loosen the bolts mounting the pressing plate to the upper plate assembly. There must be sufficient clearance for the 20-gauge sheet metal to slide between the Outer Framing Walls and the washer/bolt head.
  2. Slip the sheet metal flanges over the edge of the Outer Framing Walls and roughly align the mounting slots with the bolts on the press assembly. Once aligned, evenly apply pressure at each of the 4 corners to slowly compress the insulation and push the enclosure down onto the plate assembly. This alignment can be seen in Fig. 33a.

**Fig. 33:**
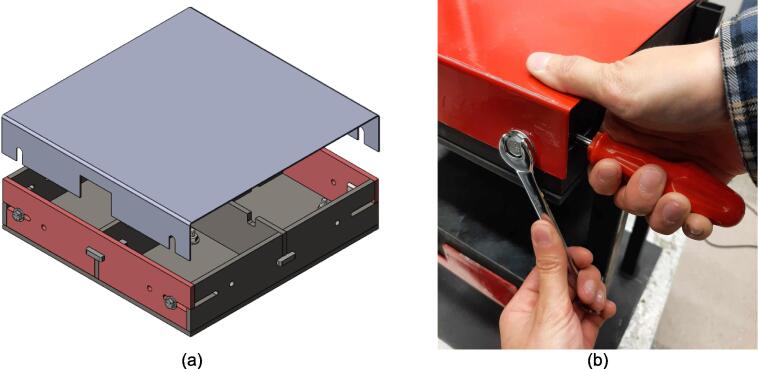
(a) Alignment and mounting of the upper Sheet Metal Enclosure, (b) Method for adjusting the mounting bolts for the upper Sheet Metal Enclosure from the outside while preventing the interior nuts from freely spinning.
  3. Once the face of the Sheet Metal Enclosure is flush with the body of the press and all four mounting slots are located on a bolt, the nuts can be tensioned in place.
  a. Tip: Insert a screwdriver into the adjacent slot in the plate assembly to the bolt of interest. Lever the screwdriver up to apply a downward force on the interior nut to lock it in place, while tightening the bolt from the outside as shown in Fig. 33b.
C) Lower Sheet Metal Enclosure
1. Loosen the nuts mounting the lower plate to the lower plate assembly. There must be sufficient clearance for the 20-gauge sheet metal to slide between the Outer Framing Walls and the washer/bolt head.
2. Slide the LH lower Sheet Metal Enclosure over the mounting bolt on the left-hand side of the assembly. Ensure the hole in the enclosure is in line with the hole in Jack Support L1. The orientation is shown in Fig. 34a.

**Fig. 34:**
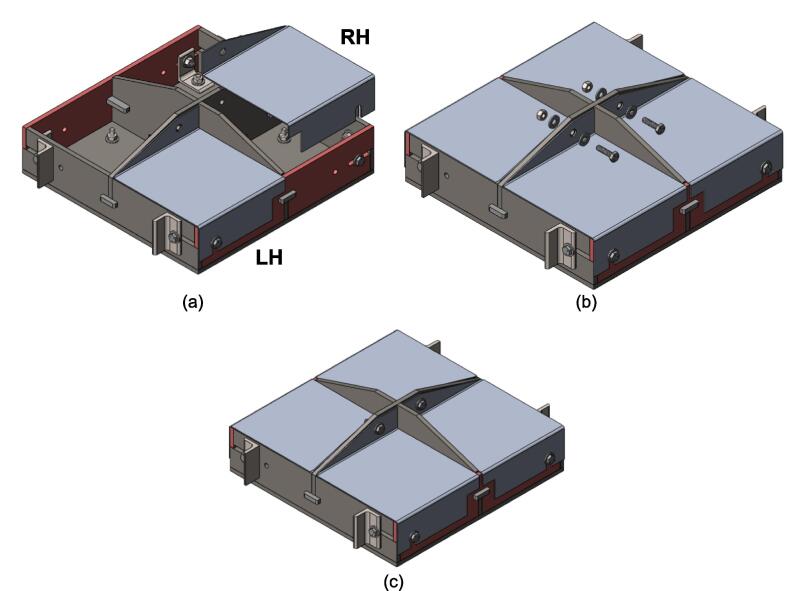
(a) RH and LH Lower Sheet Metal Enclosure alignment with lower plate assembly. (b) Securing lower Sheet Metal Enclosures. (c) Final Lower Plate Assembly.
3. Slide the second LH lower Sheet Metal Enclosure over the quadrant diagonal to the previously mounted enclosure.
4. Slide a RH lower Sheet Metal Enclosure over the mounting bolts in the two remaining quadrants.
5. Once all 4 enclosures are in position, add a washer to a 1/4–20 x 1″ HHCS and feed one through each side of the Jack Support L1 before tightening a 3/16″ washer and 1/4″ nut in place as seen in Fig. 34b.

#### 5.3.4 Mechanical Reassembly

Once the Upper and Lower Sheet Metal Enclosures have been installed, the Plate Assemblies are complete and ready to be returned to the Support Frame.

1. Return the bottle jack to the Support Frame assembly with the handle positioned closest to the electrical enclosure Support Leg.
  a. Place the 3″ x 3″ Jack Support plate on the saddle of the jack to assist in distributing the load across the Jack Support frame.
2. Lift and slide the lower plate assembly over the Support Legs of the support frame and rest the Linear Guides on the Lower Plate Supports as shown in Fig. 35.

**Fig. 35:**
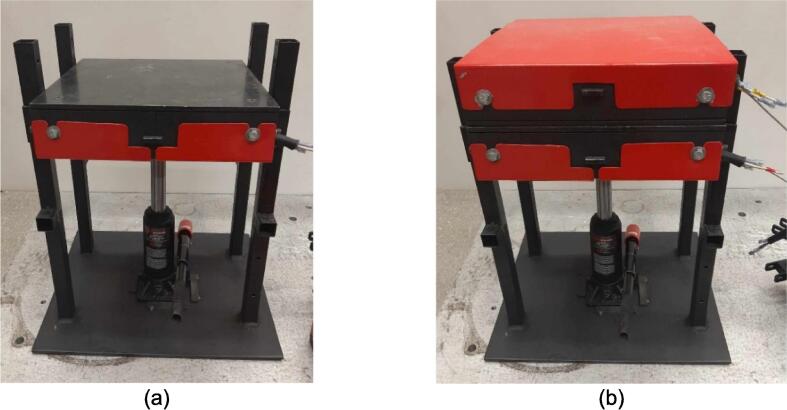
Integration of the upper and lower plate assemblies into the support frame. (a) Aligning and mounting the lower plate assembly, (b) placing the upper plate assembly on top and tensioning it in place with the SHCS.
3. Place the upper plate assembly on top of the lower plate assembly.
4. Actuate the bottle jack to lift the plate assemblies together until the mounting holes on the Support Legs are aligned with the mounting holes on the upper plate assembly as shown in Fig. 35b.
5. Add a 3/16″ washer to four 1/4–20 x1 3/4″ SHCS and feed one through each of the Support Legs before threading them through the previously welded 1/4″ nut on the inside of the upper frame assembly.

Once the Upper Plate Assembly has been installed the mechanical assembly is complete. For general maintenance and performance, the Support Legs can be coated in a thin layer of Moly grease to encourage smooth motion. Before using the machine, the lower plate assembly alignment should be double checked, and the Linear Guides appropriately adjusted. Actuate the jack up and down slowly while observing the Linear Guides. If the system binds at any point, the Linear Guides can be loosened off and gently slid along the slot they are mounted in to adjust their linearity relative to the Support Legs. The bolt can then be retightened in place and the jack cycled once more to evaluate the success of the adjustment. These Linear Guides are intended to accommodate any inaccuracies acquired during welding and assembly by utilizing both a slot for separation adjustments, and a single bolt for rotation. The jack must be cycled until the motion of the system is smooth at which point the bolts can be fully tightened in place. Similarly, if the system is binding at the top of jack’s stroke, the bolts on the Support Legs holding the upper plate subassembly in place can be loosened slightly. If the system begins binding at any point over its lifetime this procedure can be repeated to realign the system.

**Fig. 36:**
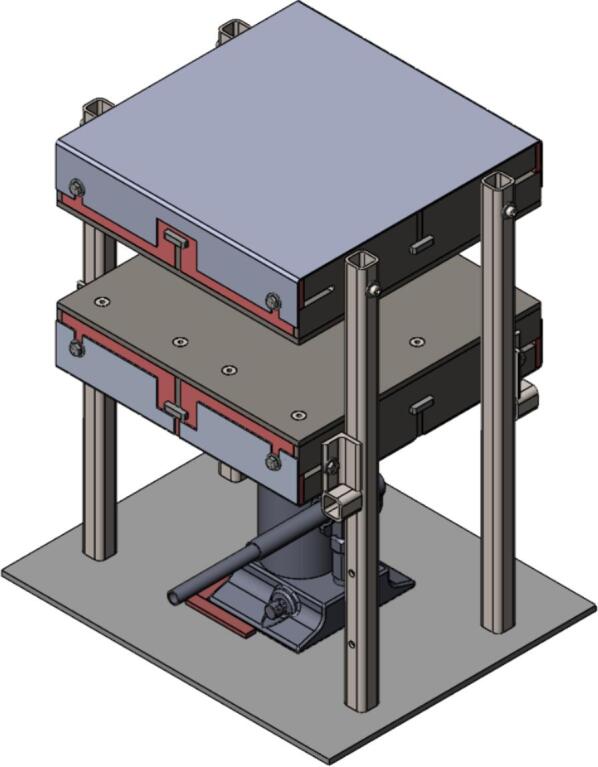
Final mechanical assembly rendered.

With the mechanical assembly completed as shown above in Fig. 36 above, and the leads for the thermocouples and elements accessible from outside of the frame assemblies, the build can transition to electrical enclosure and integration. Should any adjustments to the heating elements be required, the upper plate assembly can conveniently be serviced by removing the upper Sheet Metal Enclosure in its standard position. For the lower plate subassembly, the upper and lower plate assemblies can be slid off the support frame all together and flipped to rest on their Steel Platens.

### 5.4 Electrical Assembly Instructions

The following section outlines the components necessary for 3-D printing the electrical enclosure, how to prepare the electrical components for installation, assembling the electrical enclosure within these components, wiring the system, and finally integration with the mechanical assembly. Note that 3-D printed components are not constrained by the same stock limitations as the steel used for the mechanical assembly. As a result, the electrical enclosure and all hardware from electronics to fasteners are more appropriately metric.

The electrical subsystem features a graphic user interface (GUI), built using an I2C-enabled liquid crystal display (LCD) and a rotary encoder. A step-down alternating current/direct current (AC/DC) converter serves as the power supply unit (PSU) low-power system in this configuration, enabling user-friendly interaction and independent functionality. However, should the user prefer to reduce the system cost, they can connect via USB to a PC and operate the machine using just the serial interface on their computer using text-based commands to eliminate the need for the PSU module, rotary encoder, LCD display, and the SD logger. The Arduino Nano ESP32 was selected for use based on its compact construction, energy-efficiency, and cost-effectiveness [52] demonstrated across a wide range of applications including basic control systems for biological applications [53], [54], [55] and manufacturing equipment [56], [57], [58] to intricate Internet of Things (IoT) devices [59], [60]. Their utilization enables developers to segment applications into numerous execution domains, facilitating precise control over performance, features, and power requirements [61].

#### 5.4.1 Manufacturing (3-D printing) the Electrical Enclosure

**Fig. 37:**
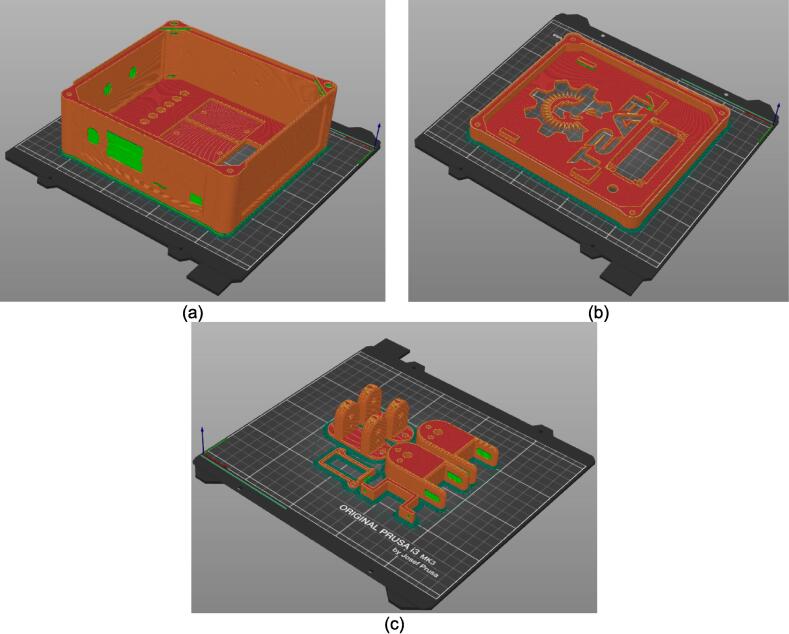
Slicer view of manufacturing build plate batches and support locations. (a) Electrical Enclosure Body, (b) Electrical Enclosure Lid, (c) Electrical Enclosure Mounting Hardware (Item 3, 4, 7, 8).

The electrical enclosure was designed for manufacturing using 3-D printing within the build volume of a Prusa i3 MK3S+. Polyethylene terephthalate glycol (PETG) was selected for the print material as a durable, electrically insulative, and a widely accessible 3-D printing filament. Further, the alternative use of PETG scrap recycled in a DRAM context with a recyclebot [4] or direct extrusion system [18] offers an additional method of promoting sustainability within the system. Coupling recycled PETG use with solar-powered 3-D printers [25], [62], [63], [64] introduces the potential to essentially eliminate material costs [6]. Similarly, other readily available thermoplastics with comparative durability, elevated melting temperatures, and 3-D printing compatibility such as acrylonitrile butadiene styrene (ABS) or PC could also be used. When selecting alternative materials, the melting and glass transition temperature must be closely considered as the enclosure must sustain the elevated ambient temperatures of the assembly, conducted by the supporting frame, and the internal relays used. As a result, using materials such as PLA with lower melting temperatures is not advised and could result in a deformed or damaged electrical enclosure at these interfaces over time. The 3-D printed components necessary for this build are shown in Table 8 below, and the plater orientation, supports, and batches that correspond to the print times previously described in Table 5 shown in Fig. 37.

**Table 8:** Visual BOM of the 3-D Printed Enclosure Components.

**Item #**	**Component Designator**	**Rendering**
1	Electrical Enclosure Body	
2	Electrical Enclosure Lid ORSolid Electrical Enclosure Lid	
3	Electrical Enclosure Mounting Bracket	
4	Electrical Enclosure Mount	
5	Electrical Enclosure Rear Cover	
6	PSU Bracket	
7	Nano Bracket

The electrical enclosure design is simple but provides the user with the opportunity to customize the components and minimize the amount of 3-D filament and print time necessary. To achieve this, the assembly can be assembled through one of two approaches: (a) all components are 3-D printed, meaning the user must select the solid electrical enclosure lid and the rear cover, (b) the user can form a rear cover and internal front cover to fill the through-all window in the lid on the sheet press using a recycled soft plastic such as HDPE. This second option is recommended and will be solely referenced in this build as it not only reduces time and cost, it also offers an artistic feature to the system and an ideal use case for the sheets produced in the initial trials of the machine. The electrical enclosure can be left unsealed during preliminary testing, and later closed with the results of these first tests.

The following manufacturing and assembly instructions are specific to choosing to produce a recycled HDPE front and rear cover and does not include detail on the 3-D printed approach; however, the necessary STL, 3mf, and G-code files can be found on the OSF for each component and the difference in cost and time is outlined in the Table 5 BOM.

To prepare the components of the Electrical Enclosure in Table 8 for 3-D printing, the parts can be reproduced in one of the following ways.

a. If a Prusa i3 MKS+is accessible, the G-code files can be directly uploaded to the printer.
b. If the preconfigured manufacturing settings are desired, but a different 3-D printer available, the 3mf files can be downloaded and the models resliced for the new 3-D printer.
  i. The settings used for this build are specified in Table 9 below for reference but may need to be modified to suit a different 3-D printer. All settings not mentioned in the table utilized the “0.2 Quality” automatic preset for generic PETG on the PrusaSlicer-2.4.2.

**Table 9:** Printing parameters used to print the enclosure on the Prusa i3 MK3S+.

**Setting**	**Specification**	**Value**
Plater	Print Settings	0.2 mm QUALITY (modified)
	Filament	Generic PETG (Polymaker PETG)
	Printer	Original Prusa i3 MK3S & MK3S+
	Supports	Everywhere
Print	Layer Height	0.2 mm
	Infill	20 %
	Skirt and Brim	5 mm Brim
	Support Material	Overhang Threshold: 0° Generate Support MaterialStyle: snug
Filament	Filament	Nozzle Temperature: 250°CBed Temperature: 85 °C
Printer	Extruder 1	0.40 mm Nozzle
c. To print the designs for a different 3-D printer, with customized settings, or prepare the G-code using a different slicer all together, download the STL files and slice them as needed.
  i. Set the 3-D printer parameters in the slicer software according to the printer-specfiic recommended settings for the specific printer and material and in the ideal orientation shown in Fig. 37.
  ii. These parameters can be determined by the instructions provided by the machine and the material manufacturer.
d. To make changes to the design, such as tolerances, component variations, or new features, download the SLDPRT or STEP files and modify them accordingly in a CAD program of choice.

Once the 3mf has been prepared, the print can be started. Note that the following section outlines how to prepare the parts for assembly once they have been 3-D printed, however, as this process is time consuming, the electrical components can be prepared during the wait time according to Section 5.4.2.

1. Export the finalized G-code for all 3-D printed components and quantities outlined previously in Table 5 and load it onto the 3-D printer.
2. Initiate the 3-D printing process and monitor the initial layers to ensure successful bed adhesion and error-free printing. If available, monitor the entire print with computer vision/AI [65].
3. Once the print is complete, remove the component(s) from the printer bed. Use caution to avoid unnecessary damaging of the printer and printed parts.

A) Assuming the prints are free of defects, remove all support structures attached to the prints using a combination of mini side cutters, needle-nose pliers, or a hobby knife.

B) Safety glasses are recommended while removing supports for eye protection in the event of brittle failure.

4. Once the print is free of supports, double check the tolerances on each of the holes by feeding an M2, M3, or M4 bolt through their respective holes. If they are too tight to pass through, use the appropriate drill bit to resize these holes.
  a. Confirm the LCD screen fits in the Electrical Enclosure Lid.
  b. Confirm that the Electrical Enclosure Lid edge creates a lap joint over the Electrical enclosure body without interference.
5. Once the fits are satisfactory, press fit (X8) M3 nuts into the slots at each of the 4 corners of the Electrical Enclosure Body as identified in Fig. 38 below. Ensure the nut is oriented such that two flat sides run parallel to the internal walls of the slot (perpendicular to the entrance direction). Once the fit has been started, use a small Allen key to push the nut the remainder of the way.

**Fig. 38:**
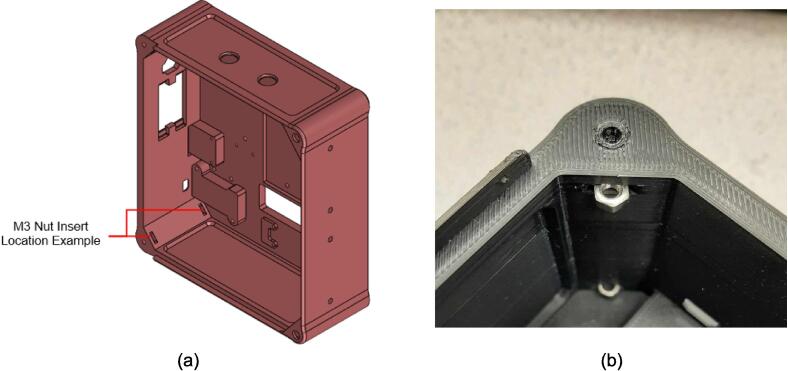
(a) Enclosure body with M3 nut press fit locations identified in one of the corners. Each corner of the enclosure has an additional two M3 nut locations along the same plane. (b) Orientation and location of press fit nuts prior to insertion.
6. Press fit (X6) M4 nuts into the recesses on the inside face of the Electrical Enclosure Body near the top as shown in Fig. 39.
  a. To ease the process, thread an M4 x 15 FHCS into each hole from the outside of the enclosure to the inside. Ensure the nut is in line with the profile of the recess and tighten each with an M2.5 Allen key. This will draw the nut naturally into the recess.

**Fig. 39:**
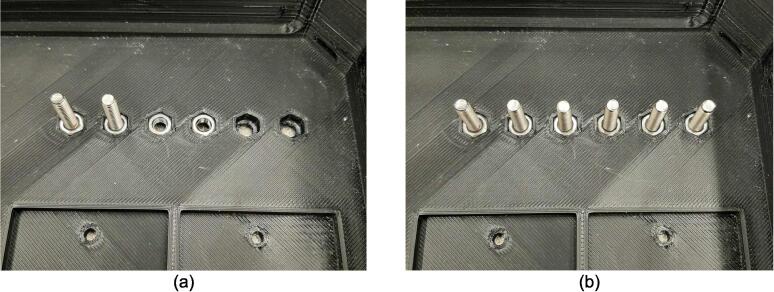
(a) Press fit procedure for M4 nuts into Electrical Enclosure Body, (b) Finished M4 screw posts.
7. The exposed threads of the FHCS will later act as the live, neutral, and PE screw posts for the heaters.

At this point, if the print quality and tolerances are satisfactory and all components have been printed in the appropriate quantity, the electrical enclosure is ready to be assembled with the electrical components.

#### 5.4.2 Preparation of the Electrical Components

Prior to installation of the electrical components into the Electrical Enclosure, certain components must first be prepared with pin headers, ring and blade terminals, wires, etc. for interfacing. The following components must be soldered or otherwise prepared prior to installation:

a. LCD Screen and I2C Converter Backpack
b. Arduino Nano ESP32
c. MAX31855 Board (X2)
d. SD Card Logger
e. Double Sided PCB Protoboard 2x8cm
f. High-Power Wires
g. Relay Low-Power Wires
h. AC Power Entry Receptacle

Set the soldering iron temperature to 370°F (188 °C) for lead-based and 420°F (215 °C) for lead-free solder, which are suitable temperatures for standard-sized joints and efficient tips. For irons with small, less efficient tips or larger joints, higher temperatures may be used. However, note that higher temperatures can lead to overheating, resulting in damage or accelerated oxidation of the tip.

A) LCD Screen and I2C Converter Backpack.

**Fig. 40:**
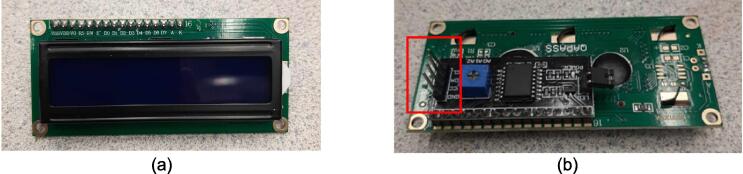
(a) Front of purchased LCD screen, (b) Rear side of the LCD screen and soldered I2C converter backpack module. Header pins on the left side of the I2C converter backpack module are bent perpendicular to the screen.

The LCD Screen was purchased with the I2C converter backpack soldered in place. The four header pins on the face of the I2C converter backpack module must be carefully bent from their stock horizontal position to be perpendicular to the LCD screen as shown in Fig. 40b. This is necessary for ensuring the electrical enclosure lid has sufficient clearance to mount to the body.

B) Arduino Nano ESP32.

1. Solder the header pin strips provided with the Arduino Nano ESP32 with the connection (long) side facing upwards (side with the USB connector) as seen in Fig. 41a.

**Fig. 41:**
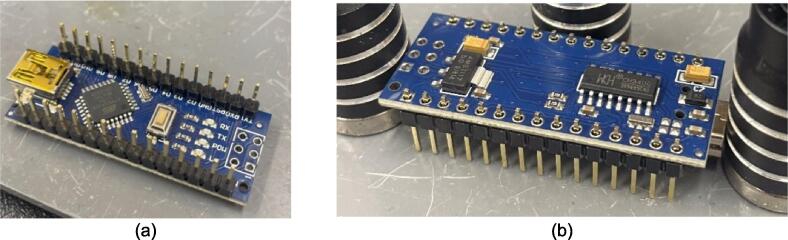
(a) The proper orientation of the header pins on the Arduino Nano ESP32, (b) Positioning and fixing of the Arduino Nano for soldering using magnetic soldering holders.
2. Position and secure the Arduino Nano ESP32 to anchor the header pin strips in place for soldering. Due to their length, manually holding each flush introduces unnecessary challenge and a higher risk of displacement while applying solder.
a. To anchor the board for soldering, physically block three of the sides as shown in Fig. 41b with soldering holders. Solder each pin in place.

C) Adafruit MAX31855 Breakout Boards.

1. Solder the 6 pin header strips provided with the two Adafruit MAX31855 breakout boards so that the connection (long) side is facing upwards (toward the MAX31855 integrated circuit).
2. On the same side, solder the 2-pole block terminal such that it faces toward the edge of the PCB and away from the 6 pins.
  a. Position and secure the Adafruit MAX31855 breakout boards on 3 sides as shown in Fig. 42a.

**Fig. 42:**
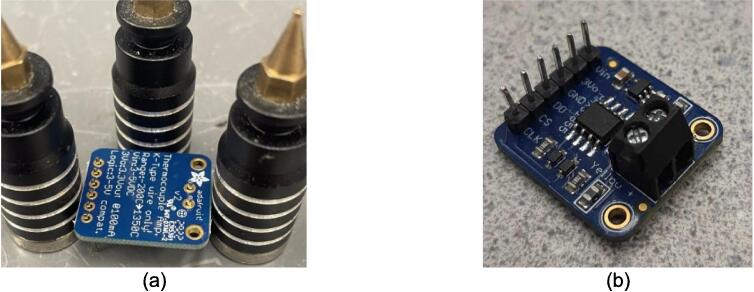
(a) Positioning and fixing of the MAX31855 Thermocouple Amplifier breakout board for soldering using magnetic soldering holders, (b) Completed MAX31855 Thermocouple Amplifier breakout board.
3. Repeat for the second board.

D) Adafruit MicroSD Card Breakout Board.

**Fig. 43:**
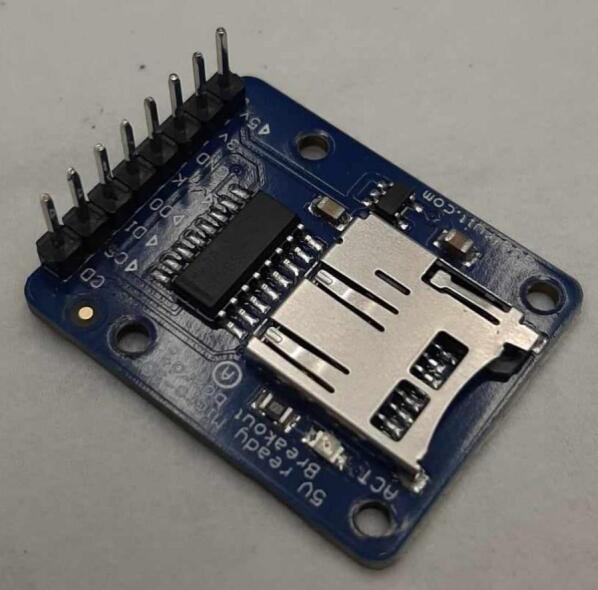
MicroSD Card Module.

1. Solder the 8 pin header strips provided with the Adafruit MicroSD card breakout board + with the connection (long) side facing upwards (side with the SD card socket) as shown in Fig. 43.

E) Double Sided PCB Protoboard.

1. Cut a piece of protoboard to a 6x10 grid of holes using sheet metal shears.
2. Solder two 8 pin header strips length wise on the first and sixth rows, and two 5 pin headers on the third and fourth rows as shown in Fig. 44.

**Fig. 44:**
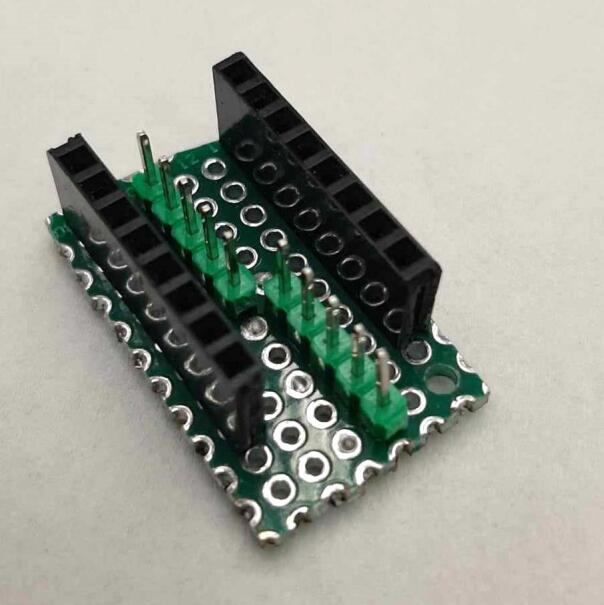
Soldered double-sided PCB protoboard.
3. Create solder bridges between all pins within each header strip to short them together, forming a bus on each header.

F) High-Power Wires.

In preparation for wiring the high-power components, prepare the wire lengths found in Table 10 using the appropriately 20AWG (American Wire Gauge) solid wire. Ensure that the correct color is used for each wire as it corresponds to the Canadian Electrical Code convention for mains wiring. According to section 4-032 of the Canadian Electrical Code, in a 2-wire system with a protective earth, insulated conductors must be color-coded: black for live/hot, white for neutral, and green for earth/ground conductors (Subrules 1–3) [66]. The high-power electrical wiring connects the AC power entry receptacle, circuit breaker, Solid State Relays (SSRs), and heating elements to safely provide mains AC power to heat the press. The AC input is connected through the circuit breaker (protection device) to the SSRs (switching device), which control power delivery to the heaters. *It is strongly recommended that good practices are observed with respect to labelling all wires, components, and connections especially if the same color of wire is used throughout, as is here.*

**Fig. 45:**
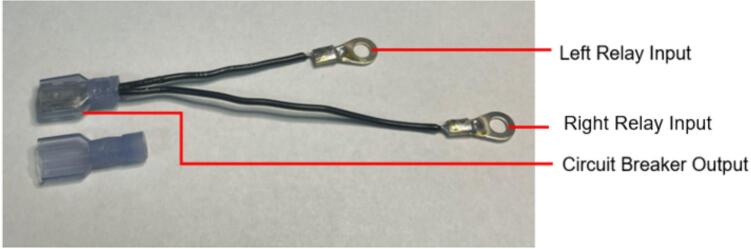
Prepared Breaker Output Wires.

1. Use wire strippers to remove 5 mm of insulation from the ends of each wire.
2. Use a soldering iron to tin the stripped ends of the wires prepared in the previous steps using solder.
  a. To 'tin' a wire, heat the exposed end with a soldering iron and apply solder to form a thin, even layer.
  b. This process helps to improve the electrical connection and eases the process when soldering the wire to terminals or other components.
3. For wire terminations specified as ring terminals, use an appropriate crimping method and tool to secure the ring terminal to the wire.
  a. If using uninsulated ring terminals as used here, solder can be applied to further secure the connection unless specified otherwise by the component manufacturer.
4. For the two female blade terminals used to service the breaker, use an appropriate crimping method and tool to secure the ring terminal to the wire.
  a. Remove the bottom part of the insulated sleeve to allow two wires to be crimped to the breaker output terminal as seen in Fig. 45, the empty terminal casing is pictured for comparison to indicate the removed portion.

**Table 10:** High-Power Wiring Specifications.

**Name**	**Length (mm)**	**Color**	**Terminal 1**	**Terminal 2**
**Inside the Enclosure – Solid Wire 20 AWG**				
Receptacle Live (Entry) to Switch Live Input	30	Black	Bare	Bare
Switch Live Output to Circuit Breaker Input	65	Black	Bare	Blade (F)
Circuit Breaker Output to Left Relay Input	50	Black	Blade (F)	Ring
Circuit Breaker Output to Right Relay Input	80	Black	Blade (F)	Ring
Left Relay Output to Left Live Screw Post	30	Black	Ring	Ring
Right Relay Output to Right Live Screw Post	30	Black	Ring	Ring
Receptacle Neutral (Entry) to Switch Neutral Input	20	White	Bare	Bare
Switch Neutral Output to Left Neutral Screw Post	100	White	Ring	Ring
Left Neutral Screw Post to Right Neutral Screw Post	50	White	Ring	Ring
Receptacle PE (Entry) to Left PE Screw Post	105	Green	Bare	Ring
Left PE Screw Post to Right PE Screw Post	50	Green	Ring	Ring
**Exiting the Enclosure – Stranded Wire 16 AWG**				
Left PE Screw Post to Upper Plate PE Wire	350	Green	Ring	Blade (M)
Left Neutral Screw Post to Upper Plate Neutral Wire	350	White	Ring	Blade (M)
Left Live Screw Post to Upper Plate Live Wire	350	Black	Ring	Blade (M)
Right PE Screw Post to Lower Plate PE Wire	350	Green	Ring	Blade (M)
Right Neutral Screw Post to Lower Plate Neutral Wire	350	White	Ring	Blade (M)
Right Live Screw Post to Lower Plate Live Wire	350	Black	Ring	Blade (M)

G) Relay Low-Power Wires.

The relay control inputs use screw connections, requiring four 20 cm jumper wires modified on one end with ring terminations.

1. Cut off the connector from one end of each jumper wire, cut close to the terminal being removed to preserve wire length.
2. Strip 5 mm of insulation from the wire, twist the bare end, and tin with solder.
3. Crimp the ring terminals using an appropriate crimping method and tool to secure the ring terminal to the wire as above.

H) Power Entry Receptacle.

**Fig. 46:**
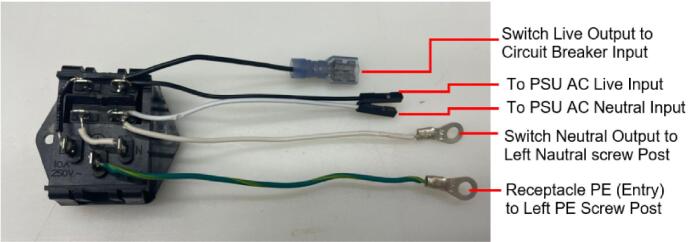
AC Power Entry Receptacle prepared with soldered wire connections in advance of installation.

1. The following wires prepared in advance will be used to wire the AC Power Entry receptacle:
  a. Receptacle Neutral (Entry) to Switch Neutral Input
  b. Receptacle Live (Entry) to Switch Live Input
  c. Switch Live Output to Circuit Breaker Input
  d. Switch Neutral Output to Left Neutral Screw Post
  e. Receptacle PE (Entry) to Left PE Screw Post
2. Take two female jumper wires (one black and one white) and cut off one connector from each, leaving the female connectors on the other end intact.
3. Strip 10 mm of insulation from the cut ends and twist the exposed strands together tightly.
4. Use a soldering iron to tin the stripped ends of the wires using solder.
5. Complete the following connections shown in Fig. 46.
  a. Begin with the PSU input wires connected to the live and neutral output tabs of the switch by threading them through their respective hole and wrapping the remaining wire around the edge of the tab.
  b. Use pliers to insert and guide the solid wire between the holes on the receptable tabs then solder in place using adequate solder to create a complete joint between the solid wire and both sides of the receptacle tab.

#### 5.4.3 Installation of the Electrical Components

Once all electrical components have been prepared for installation, they can be systematically integrated into the electrical enclosure. It is most convenient to begin with the Electrical Enclosure Body followed by the Electrical Enclosure Lid.

C) Electrical Enclosure Body.

**Fig. 47:**
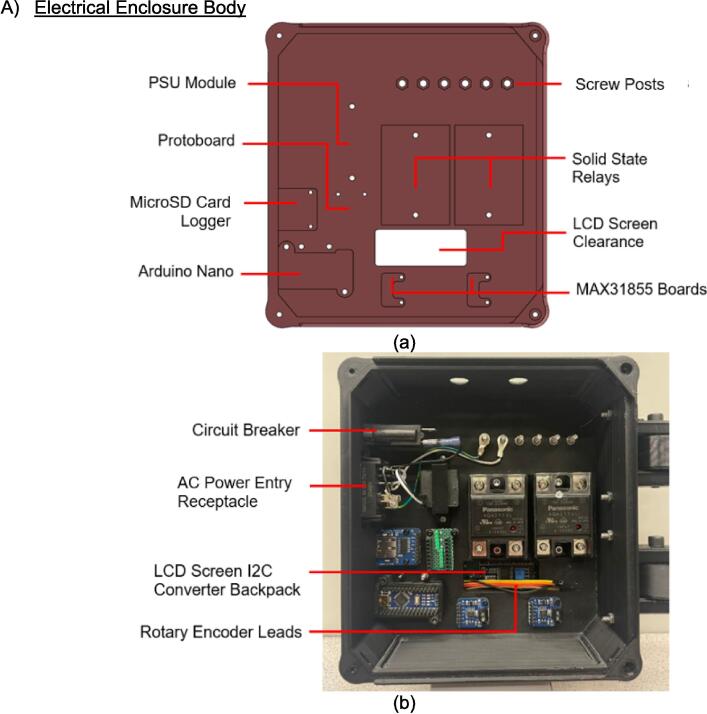
Positioning of electrical components inside electrical enclosure body. (a) CAD Inside back panel, (b) Installed electrical components prepared for connections.

For each installation procedure below, refer to Fig. 47a to confirm the location of the component and Fig. 47c for the completed mounting assembly.

1. Place an SSR in each of the large rectangular recesses in the middle-right of the Electrical Enclosure Body. For proper orientation of the terminals, ensure the two low voltage leads are biased toward the bottom of the enclosure, and the two high voltage leads toward the top.
2. Feed two M3 x 10 BHCS through each SSR and through the enclosure. Secure each SSR with an M3 nut on the back side of the Electrical Enclosure Body.
3. Feed the circuit breaker and the prepared wires of the AC Power Entry Receptacle through their respective cutouts in the sidewall of the Electrical Enclosure Body from the outside to the inside. These positions are identified in Fig. 48a. Snap both into place with the built in locking tabs.
  a. This setup facilitates power supply distribution and ensures safety through overcurrent protection.

**Fig. 48:**
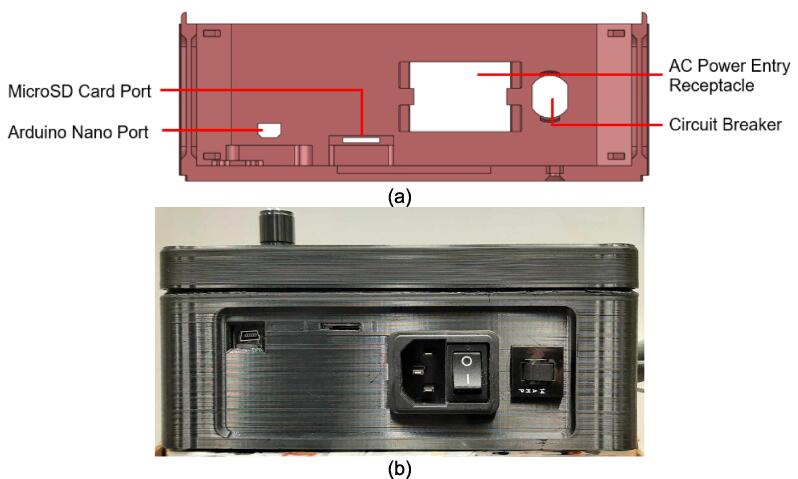
(a) Section view of the inside wall of the electrical enclosure body housing the circuit breaker and AC Power Entry Receptacle, (b) Outside perspective with all electrical components mounted.
4. Place the microSD card Logger on the square elevated platform and slide the board into the slot in the sidewall of the Electrical Enclosure Body until the card port is flush with the port hole on the outside of the enclosure and the mounting holes on the board align with the printed holes in the enclosure.
5. Feed two M2 x 20 BHCS into the rear holes of the microSD card breakout board and secure them with an M2 nut on the back side of the electrical enclosure body.
6. Place the Arduino Nano ESP32 board on the rectangular elevated platform.
7. Place the Nano Bracket over the Arduino Nano ESP32 and feed two M3 x 20 BHCS through the holes located at the corners of the bracket and platform. Secure each with an M3 nut on the backside of the enclosure.
  a. To ensure the port lines up with the hole in the sidewall, it is useful to plug in a mini-USB cable into the USB port from the outside of the enclosure prior to tightening the screws in place.
  b. The bridged end of the nano bracket must enclose the mini-USB port on the Ardino nano ESP32 to align it with the port hole.
8. Place the PSU module into the enclosure between the designated mounting holes and place the PSU bracket over top of it. Ensure the PSU is positioned such that the AC Input pins are closer to the AC power entry receptacle.
9. Feed two M3 x 15 BHCS through each mounting hole in the bracket and secure each in place with an M3 nut on the backside.
10. Align each MAX31855 breakout board with the tailored platforms in the bottom right of the enclosure such that the soldered pins from the two pole-block terminals are straddled by the platform and facing to the right.
11. Feed two M2 x 15 BHCS through each board and secure them on the backside of the enclosure with an M2 nut.
12. Align the protoboard with the designated holes in the enclosure beside the microSD card platform. Feed two M2 x 10 BHCS through the protoboard and two 2.5 x 8 mm nylon standoffs. Secure each with an M2 nut on the backside of the enclosure.
13. Flip the electrical enclosure body over to view the backside with the exposed bolts and nut connections. Place the rotary encoder in the designated recess in Fig. 49a.

**Fig. 49:**
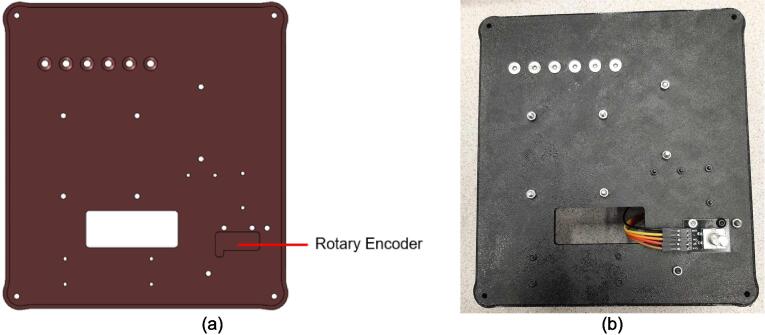
(a) Mounting location of the rotary encoder, (b) mounted rotary encoder and cable management in preparation of electrical connections.
14. Feed two M3 x 15 BHCS through to the inside of the enclosure and secure them in place with two M3 nuts.
15. Connect four 20 cm female jumper wires to the pins on the rotary encoder board and feed them up through the rectangular hole in the Electrical Enclosure Body in preparation for wiring alongside the other components as shown in Fig. 49b. Once the Electrical enclosure Lid is added, the rotary encoder will no longer be accessible for wiring. Make note of which wire is connected to which pin on the rotary encoder for future reference once the lid is closed.

D) Electrical Enclosure Lid.

1. Place the LCD screen through the cutout in the Electrical Enclosure Lid from the backside until it is flush with the lid. The location is identified in Fig. 50a.

**Fig. 50:**
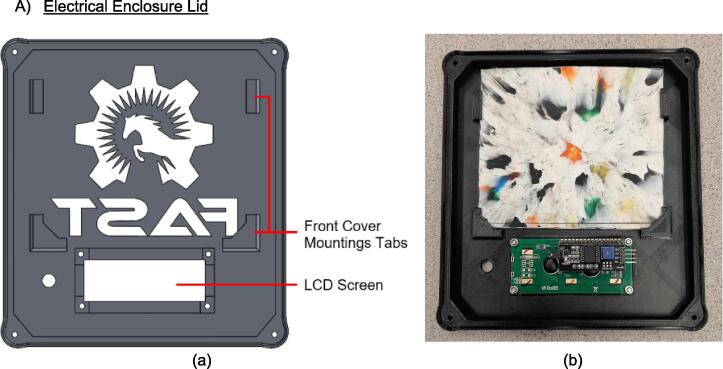
Electrical Enclosure Lid and mounting locations (a) CAD, (b) Realized.
2. Feed four M3 x 10 BHCS through the holes at each corner of the LCD screen and secure each on the backside of the Lid with four M3 nuts.
3. If using the solid electrical enclosure lid, there are no additional handling steps with the lid.
  a. If choosing to use the Electrical Enclosure Lid model used here, a suitable pressed sheet must first be manufactured with the sheet press. As a result, this component can be left out for the testing of the machine and installed later.
  b. Once a sheet has been produced, cut a roughly 124 mm x 108 mm rectangle of 16-gauge thick HDPE using sheet metal shears
  c. Gently bend the HDPE sheet until it can comfortably slide under the tabs on the backside of the Electrical enclosure Lid, effectively covering the live terminals and bolt connections, as well as acting as an artistic backdrop as shown in Fig. 50b.

#### 5.4.4 Assembly of the Electrical Enclosure

Once all the low-power and high-power electrical components have been installed, the electrical enclosure lid, Mounting Brackets, and mounts, can be installed.

1. To mount the electrical enclosure lid, align the hole in the Electrical Enclosure Lid beside the LCD screen with the shaft of the rotary encoder on the face of the Electrical enclosure body.
2. Flush the edges of the two components and feed an M3 x 20 BHCS through each corner of the Electrical enclosure lid and into the body. Tighten each screw in a diagonal torquing sequence into the press fit nuts at each corner until tight.
3. Press the knob of the rotary encoder onto the exposed shaft to complete the electrical enclosure lid. The completed front face of the electrical enclosure is shown in Fig. 52b.

At this point, all the electronics are accessible from the rear side of the electrical enclosure body, including the header pins and wiring of the LCD screen and rotary dial respectively through the clearance hole in the enclosure body. To avoid system maintenance and troubleshooting that requiring the frequent removal of a lid tethered to the inside of an electrical box with wires, a “sandwich” approach was taken for the design. The enclosure is divided into three distinct layers: a lid that allows the enclosure to still have a user-friendly HMI, a main body housing the components and wires, and finally the rear cover that is only attached by two screws and free of any electrical connections. The inverted nature of the lid and body allow for an air gap between the two components to neatly house all the exposed nuts, and live terminals, as well as a way to isolate all of the wires from any components that would need to be frequently moved.

Once the electrical enclosure lid has been secured to the body, the Mounting Brackets and mounts can be installed in preparation for mounting the enclosure to the Support Leg of the sheet press.

4. Feed a 1/4–20 x 1 5/8″ SHCS through each of the Mounting Brackets and Mounts in the orientation shown in Fig. 51. It is important to complete this step before attaching the Mounting Brackets to the Electrical Enclosure Body as there will not be enough space to feed the bolts through afterwards to act as swivel pins facilitating the enclosure’s rotation.
  a. Similarly, it is important to ensure the bolt is fed through in the correct orientation to guarantee the pivoting direction is correct. When viewed from the perspective of Fig. 51a, the position 2 hole should be biased down.
  b. Slide an M3 x 35 SHCS through the Position 1 hole of the mount and bracket.

**Fig. 51:**
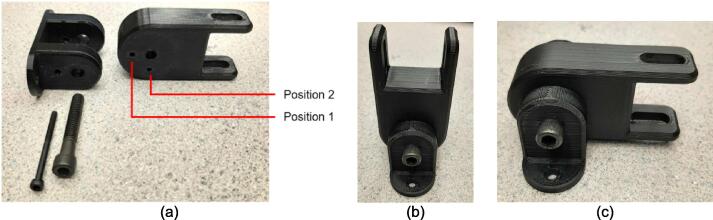
Orientation of the two lockable positions 90° relative to each other. (a) Position 1, perpendicular to the support frame, (b) position 2, tucked against the support frame.
5. Align each Mounting Bracket to the hole pairs on the lefthand side of the electrical enclosure body such that the bolt heads are facing up. Feed two M4 x 10 BHCS through each Mounting Bracket to the inside of the enclosure and secure each with an M4 nut as shown in Fig. 52a.

**Fig. 52:**
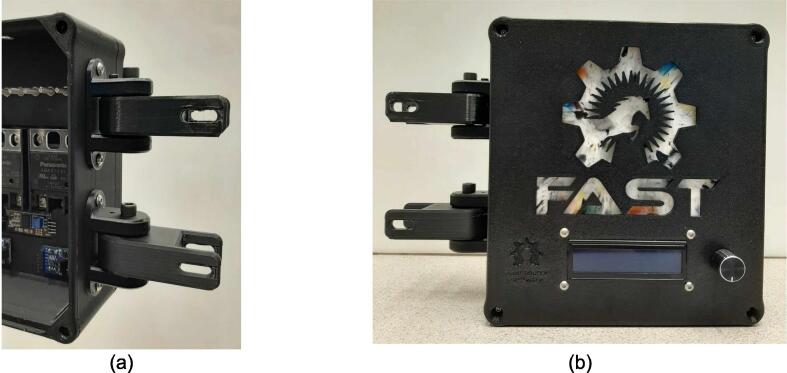
(a) Positioning and integration of the Mounting Bracket assembly from the rear of the enclosure, (b) Completed assembly from the front of the enclosure with the lid mounted.

**Fig. 53:**
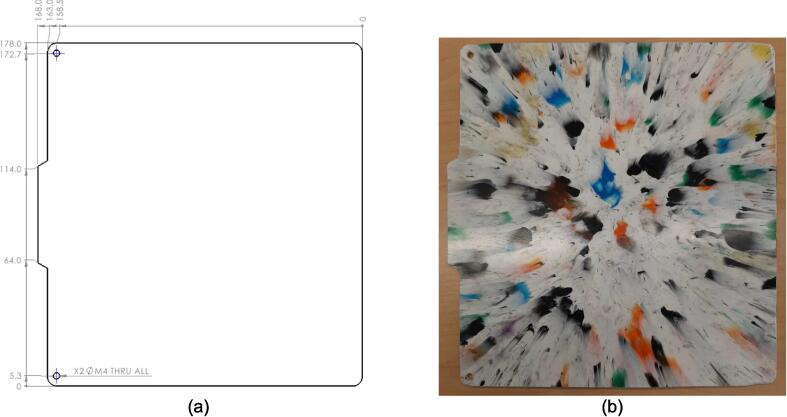
Recycled Rear Cover alternative to 3-D printed Rear cover. (a) Dimensioned drawing, all dimensions in millimeters, (b) Finalized Rear Cover produced in the sheet press.

At this point, the electrical enclosure is ready for electrical connections. The only remaining component is the electrical enclosure rear cover which can be 3-D printed or prepared later during preliminary trials with the decorative front cover. If the recycled approach is taken, and a sheet has been prepared, draw, cut, and drill the electrical enclosure rear cover from a 16-gauge thick sheet of HDPE according to the drawing in Fig. 53. The rear cover will be installed after all electrical connections have been complete.

#### 5.4.5 Low-Power Electrical Connections

**Fig. 54:**
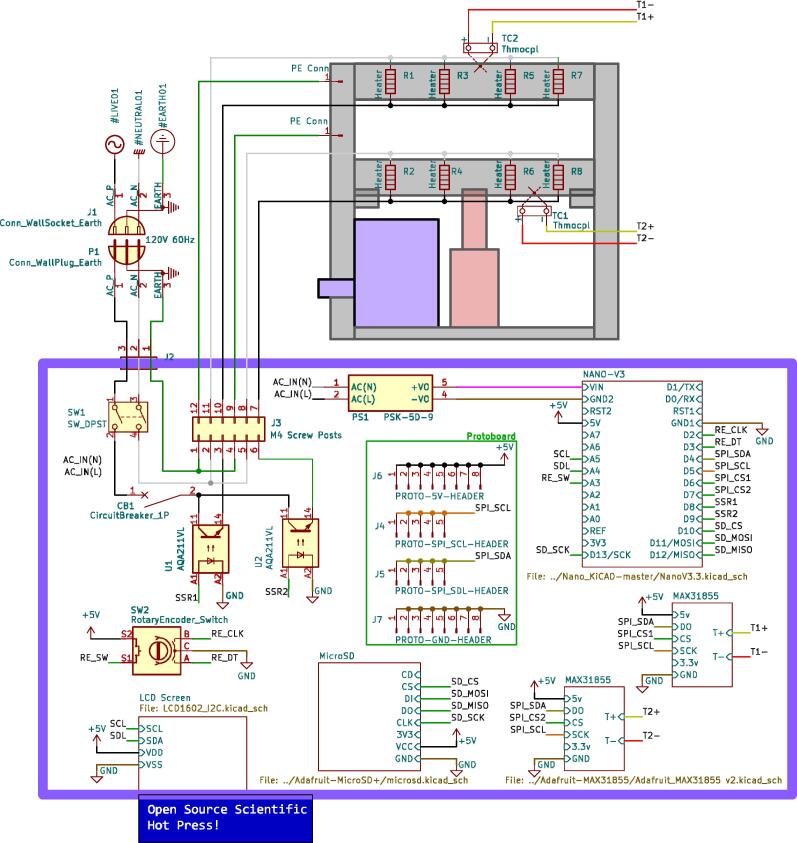
Schematic of the Complete Electrical System.

All low-power connections are completed using 10 cm or 20 cm jumper wires according to the schematic in Fig. 54. The low-power integration relies on using a combination of male and female wires as necessary to interface between the different modules and the protoboard. Where possible, use shorter 10 cm wires for a neater assembly.

1. Use the two 8 pin headers on the protoboard as power and ground busses.
2. Use the two 5 pins headers as buses for the clock (CLK) and data (DO) lines for the MAX31855 boards.
3. Use the four jumper cables with ring terminals prepared in section 5.4.2 G) for the relay inputs.

#### 5.4.6 High-Power (Mains) Electrical Connections

**Fig. 55:**
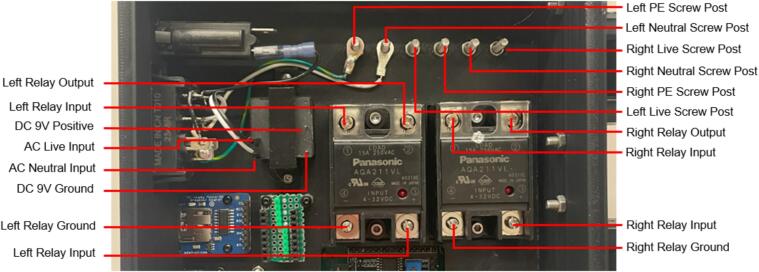
High-power component labels and relevant connections.

To complete the high-power electrical connections, refer to Table 10 for the complete list of wires and the intended connections that each must span. Cross reference the listed destinations for the terminals of each wire as they appear in Table 10 with the labels in Fig. 45 and Fig. 46 above, and Fig. 55 below. Note that for each connection involving a screw post, an M4 nut must be tensioned in place above the connection before moving onto the next section. Once each connection is complete, the assembly is prepared for integration with the heating elements and support frame.

#### 5.4.7 Heater assemblies to Electrical Enclosure Integration

Solid wire was used inside the box for its ability to hold shape and maintain connections, while stranded wire was used outside for its flexibility and resistance to wear. The wire gauges selected are rated above the outlet's maximum current capacity, providing a conservative safety margin. The six lengths of solid wire cut and prepared with ring terminals and male blade terminals in Table 10 will be used for these connections. A cluster of one of each green, white, and black wires will be assigned to the upper plate, and the remaining three of the same to the lower plate.

**Fig. 56:**
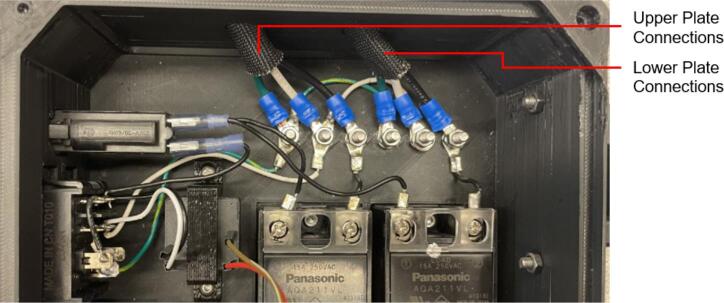
Correct wiring connections and cable management using cable sheath to cluster the wires for the upper and lower plate assembly heaters.

1. Feed the ring terminal of the three colored wires associated with the lower plate assembly through the righthand hole at the top of the electrical enclosure body (when viewed from the back and looking at the electrical components).
2. Feed the cluster of three wires associated with the upper plate assembly through the lefthand hole in the same orientation.
3. Connect the ring terminals of each wire by color according to the assigned screw post identified by name in Table 10 and labeled in Fig. 55.
4. Thread an M4 nut onto each screw terminal and tighten in place to secure the leads.
5. Cut two 13″ lengths of cable sheath and feed each over the male blade terminals of each cluster of wires exiting the electrical enclosure for the upper and lower plate assemblies. Feed the cable sheath past the hole in the sidewall of the enclosure to ensure the cables are adequately protected. The blade terminals must be exposed at the opposite end.
6. The completed wiring with appropriate connections and cable sheathing are shown in Fig. 56.

At this point in the build, the electrical enclosure is ready to be installed on the support frame and connected to the heater elements.

7. Align the slots on the electrical enclosure mounts with the holes on the electrical enclosure Support Leg.
8. Add a 3/16″ washer to two 1/4–20 x 1 3/4″ SHCS and feed one through each of the mounts. Note the washers are critical to distribute the load across the layer lines of the 3-D print and prevent cracking.
9. Add a 3/16″ washer to the other side of each SHCS and tighten the electrical enclosure in place with a 1/4″ nut.
10. With the electrical enclosure securely fixed, feed the thermocouple lead exiting the lower plate assembly through the associated hole in the top of the electrical enclosure alongside the cable sheath as previously identified in Fig. 56. Repeat with the thermocouple lead from the upper plate assembly in the adjacent hole. The result is shown in Fig. 57a
  a. These steel braided cables are longer than is necessary for this application. For organizational purposes, the cables were cut, stripped, heat shrunk, and crimped to an appropriate length to reach the MAX31855 boards inside.

**Fig. 57:**
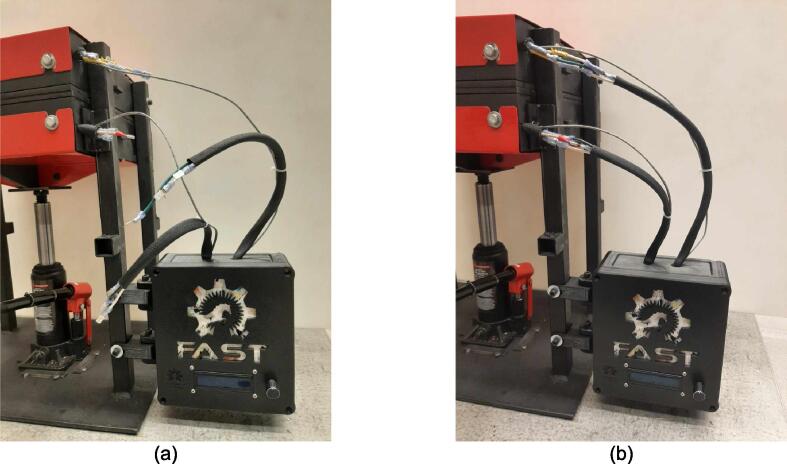
Electrical integration with the mechanical assembly. (a) thermocouple connection, (b) high-power connection to the heater circuit enclosed in each plate assembly.
11. Feed the thermocouples along the right side of the enclosure and plug them into the pole-block terminals on their respective MAX31855 boards before tensioning them down.
  a. Ensure that the thermocouple from the lower plate is connected to the left MAX31855 board (T2), and the thermocouple from the upper plate is connected to the right board (T1).
  b. To ensure proper correspondence between the thermocouple and relay, check that the left relay input connects to D8 on the Arduino and the chip select (CS) pin on the left MAX31855 board is connected to D6 on the Arduino.
  c. Likewise, the right relay input must be connected to D9 on the Arduin and the CS pin on the right MAX31855 board must be connected to D7 on the Arduino.
  d. Ensure the steel braided cases of each thermocouple are not touching any of the live terminals inside the box.
12. To complete the electrical connections and power the heaters, connect the PE (green), neutral (white), and Live (black) wires from each cable sheath cluster to the respective plate assemblies and the female blade terminals labeled accordingly. This will leave a section of exposed wire outside the cable sheath exiting the plate assemblies and entering the electrical enclosure as shown in Fig. 57b.
13. With all the wiring complete, the Electrical Enclosure Rear Cover can be slotted into the tabs on the back of the enclosure body closest to the Support Leg and screwed down using two M3 x 15 BHCS as shown in Fig. 58.

**Fig. 58:**
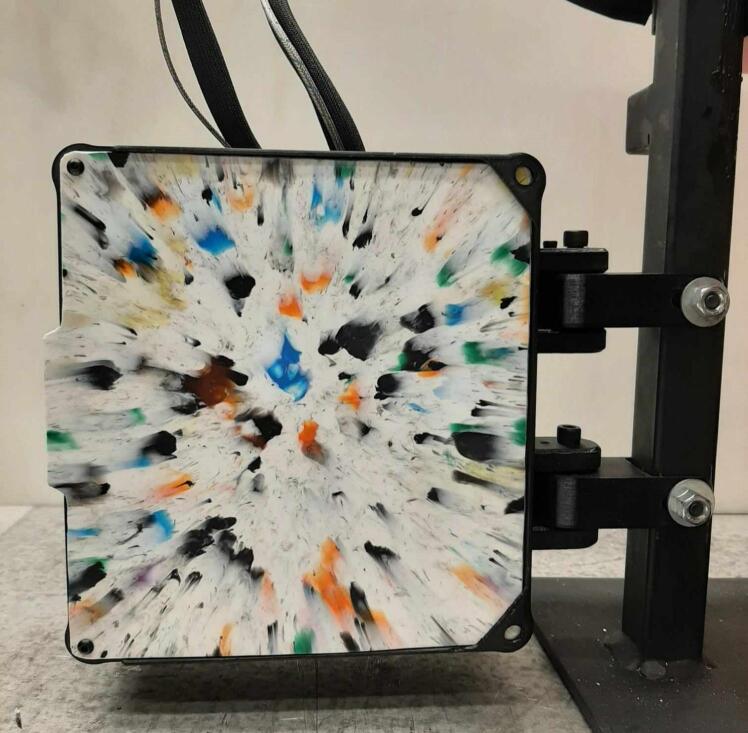
Rear Cover installation.
14. To complete the assembly, cut and add a 5″ section of split cable sheathing over the section of wire containing the blade terminals for each plate assembly. This will promote efficient troubleshooting should the need arise.
15. Add a zip tie on either side of the blade terminals to secure the split cable sheath and the thermocouple wire to the harness. Add a final zip tie halfway to the entry of the cablese into the electrical enclosure for tidiness. The completed integration is shown in Fig. 59.

**Fig. 59:**
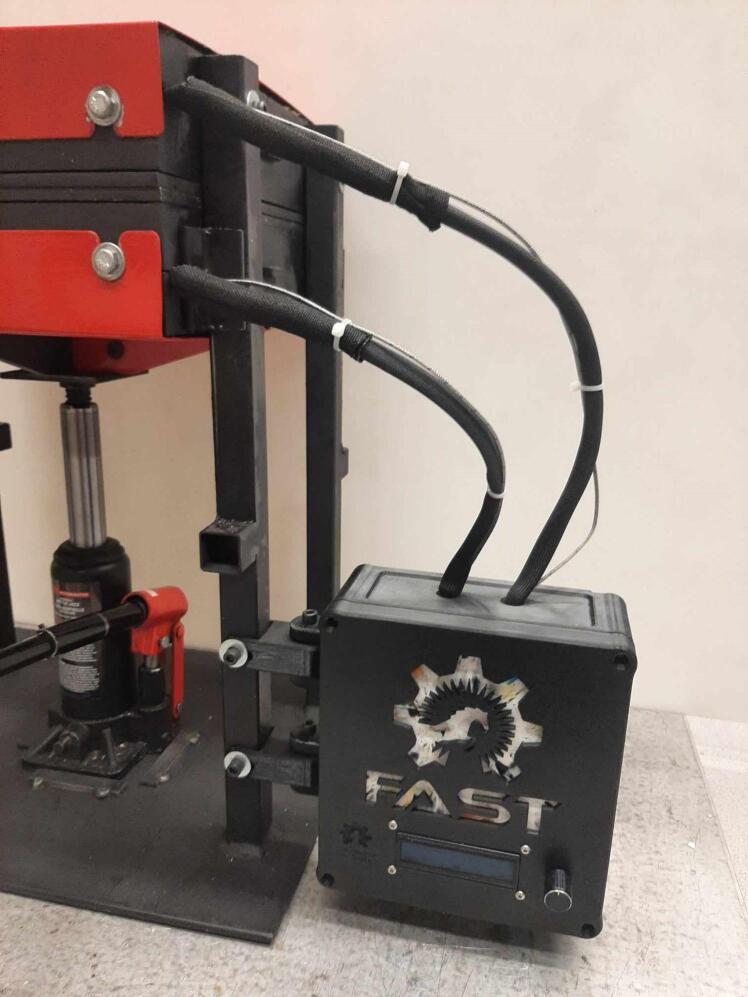
The finalized electrical enclosure and integration.

### 5.5 Software Configuration & Upload

The Arduino script initializes and configures the thermocouples, SSRs, Proportional Integral Derivative controllers (PID), display, encoder, and other components before entering a control loop. It reads plate temperatures, runs PID calculations to determine SSR duty cycles for closed-loop heating element control, handles sensor errors, updates the GUI, allows serial commands, and logs data. Key features include slow pulse width modulation (PWM) for SSR modulation, dynamic PID tuning based on temperature gaps, and redundancy using parallel thermocouples and SSRs. Together, this script enables automated temperature regulation of the upper and lower platens and effectively integrates the electrical and mechanical subsystems to create a functional, usable scientific instrument. There are two versions of the script: with the GUI handler and without the GUI handler.

To use the script:

1. Download the Arduino IDE and the required libraries listed at the top of the script code. These include the StateMachine, SD, EEPROM, PID_v1, Adafruit MAX31855, LiquidCrystal_I2C, ClickEncoder, and TimerOne libraries.
  a. All the libraries are available for download directly from the Arduino IDE, except for ClickEncoder [67].
2. In the IDE, set the board type to “Arduino Nano” and the processor to “ESP32”. Set the baud rate to 115,200 and select “New Line” under Serial Monitor settings.
3. Connect the Arduino Nano ESP32 to a computer via USB. Compile and upload the script to the board.
4. To test operation, monitor the SSR indicator lights.
  a. The script will begin heating the plates to the default 30 °C setpoint and the SSRs will light up on and off as the PID loop regulates temperature.
5. Further testing can be done by sending serial commands over USB to adjust setpoints and other parameters.
  a. The serial printouts can be monitored to view internal variables.
6. For the GUI version, the LCD and rotary encoder can be used to control and monitor the press instead of the serial monitor and command line.

When adjusting the control period it must be noted that an SSR is a non-moving contact relay utilizing semiconductor elements like thyristors, TRIACs, diodes, and transistors for switching, functioning similarly to mechanical relays but with no moving parts [68]. Under the current system settings, the SSRs are set to operate below their total charge/discharge time to prevent incomplete switching and excessive heat generation. Operating an SSR close to the maximum allowable frequencies is often unnecessary and in many cases potentially harmful to the hardware, contingent on the application.

As well, the system software provides the user with the ability to regulate this control period, or driving frequency, as demonstrated in subsequent sections. Note that the thermal time constant is the time taken for a system to reach approximately 63.2 % of its total temperature change upon a step power change [69]. Therefore, for effective thermal control, the control period should ideally match the system's thermal time constant.

### 5.6 Wiring Check

Prior to powering on the system, complete a thorough wiring check to ensure no errors have been made during assembly or loose connections have occurred from jostling. A simple procedure to follow includes the following.

1. Trace the Wiring:
  a. Confirm the live (hot) wire is first connected to the circuit breaker. This is crucial for overcurrent protection.
  b. Relay Configuration: the terminal exiting the breaker should connect to the two SSRs.
    i. These relays, controlled by the microcontroller, control power to the upper and lower plate loads.
  c. Ensure the load (heating elements) is correctly connected to the relays.
  d. Verify the neutral wire runs directly from the receptacle to the neutral side of the loads.
2. Continuity Testing and System Validation:
  a. Ensure the system is powered off and unplugged. Use a multimeter set to continuity mode.
  b. Test continuity from the receptacle’s live terminal to the input side of the circuit breaker. There should be a clear path, indicating no breaks or faults in this segment.
  c. Check continuity from the output side of the breaker to the input of the SSRs. Verify that there are no interruptions in the wiring.
  d. For each SSR, check continuity to its respective load terminals for the upper and lower plate loads.
    i. This ensures each of the heating elements can be properly connected and ready to receive power.
  e. Validate the continuity of the neutral wire from the receptacle directly to the output terminals for the load.
    i. This wire should be uninterrupted and bypass the breaker.

### 5.7 Mold Subassemblies and Build

To validate the system a series of basic flash type molds were designed and fabricated with each intended to test a different capability of the machine. The three molds selected include an ASTM D695 Mold for compression, ASTM D638 mold for tension, and a 1/4″ sheet mold as summarized in Table 11. Note that while the following section outlines these sample molds, the machine can be used for a wide variety of different forms.

**Table 11:** Visual BOM Compression and Sheet Mold.

Item #	Component Designator	Rendering
1	ASTM D695 Mold Lid	
2	Plug	
3	ASTM D695 Mold	
4	Long Framing Wall	
5	Short Framing Wall	
6	Surface Sheet	
7	ASTM D638 Mold	

#### 5.7.1 ASTM D695 Mold for Compression

**Fig. 60:**
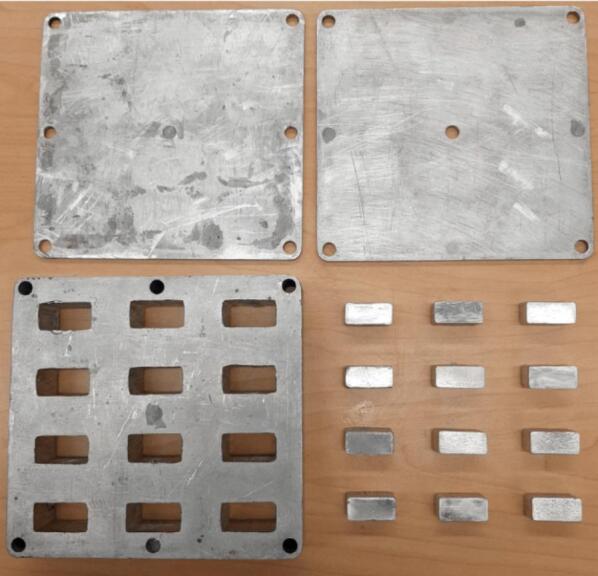
Laser cut aluminum ASTM D695 Mold Assembly for testing plastics in compression [70].

To produce material samples adhering to ASTM standards, the ASTM D695-compliant mold provided here consists of 12 individual pockets for pressing plastic samples. The manufacturing of the mold was constrained entirely to laser cutting to avoid the need for additional tooling and procedures. As a result, only the DXF files for the ASTM D695 lid, plugs, and Mold must be submitted to a local manufacturer for external services, or be laser cut in-house. Once completed, the edges of the plugs and interior faces of the mold can be gently filed down to eliminate any surface defects. The fabricated mold is shown in Fig. 60.

Note that for this build the material used for these molds was aluminum. This was selected to monopolize on the superior thermal conductivity of aluminum; however, it is recommended that mild steel be used for all molds as these began to show signs of wear at an accelerated rate due to the low ductility and hardness of the material.

#### 5.7.2 ASTM D638 Mold for Tension

**Fig. 61:**
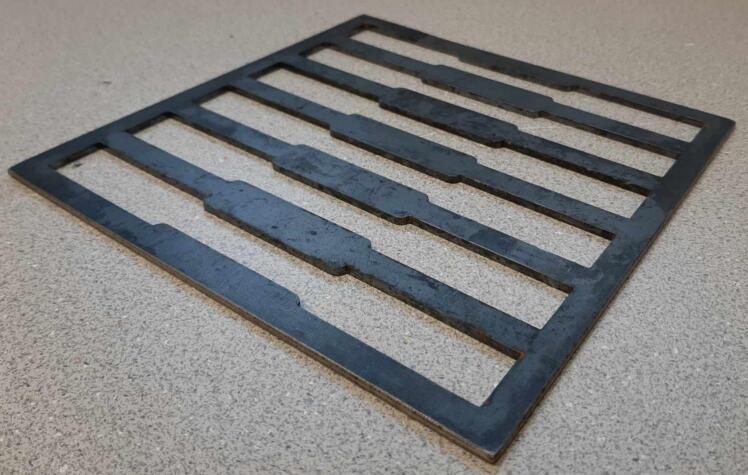
Laser cut steel ASTM D638 Mold for testing plastics in tension.

To produce material samples adhering to the ASTM D638 standard for testing plastics in tension, a Type 1 dog-bone style mold was fabricated. This mold accommodated 6 individual samples allowing a researcher to either a) guarantee uniform batch properties across a sample population, or b) vary the properties of the plastic being pressed while ensuring a controlled temperature profile shared across the samples. Like the ASTM D695 Mold for compression, this mold relied exclusively on laser cutting to reduce the tooling requirements. The fabricated mold is shown in Fig. 61.

#### 5.7.3 Sheet Mold

The basic elements for any flash mold consist of a frame to constrain the flowing plastic to a specific size and thickness, a similarly constraining upper and lower lid to keep the plastic in plane, and a means of allowing flash overflow to escape. To scale up plastic molding from the previously described small material testing samples of ASTM D695 and ASTM D638 to much larger useable sheets, an 11 1/4″ x 11 1/4″ x 1/4″ steel mold was cut and welded.

The short and long framing walls of the sheet mold assembly can be cut to size using any accessible tooling including a laser cutter with the provided DXF files, or an angle grinder, band saw, plasma cutter, etc. Should an angle grinder or plasma cutter be used, all four walls will be cut from the same purchased stock plate of 12 1/4″ x 4″ x 1/4″ to reduce cost.

1. Measure out two lengths of 12 1/4″ x 1/2″ and two of 11 1/4″ x 1/2″ and cut the short and long framing walls to size using the method of choice.
  a. For this build, a handheld plasma cutter was used. A straight edge was clamped to the table parallel the cutting direction to stabilize the tool.
  b. The same safety measures previously used for welding were used while cutting the framing walls.
2. Secure the cut framing walls and use an angle grinder outfitted with a flap disk to flatten the edges of the cut. A finer grit belt sander can be used to further smooth the surface and improve how well the mold will release when pressed.
3. Bevel the top and bottom ends of the short framing walls to approximately 30°. Bevel the inside edge of the long framing walls to approximately 30° and 1/2″ in length. See Fig. 62 below for reference position.

**Fig. 62:**
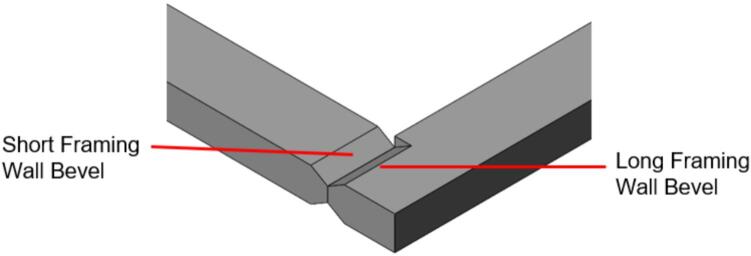
Short and Long Framing Wall mate and bevel.
4. Align the short framing walls and long framing walls to create the square form of the mold as shown below in Fig. 63 below. Ensure the surface used is flat, and the beveled corners of the short and long walls meet. Clamp each wall down and tack weld the corners together to secure the form.
  a. The same weld settings used for the hot press frame were used.

**Fig. 63:**
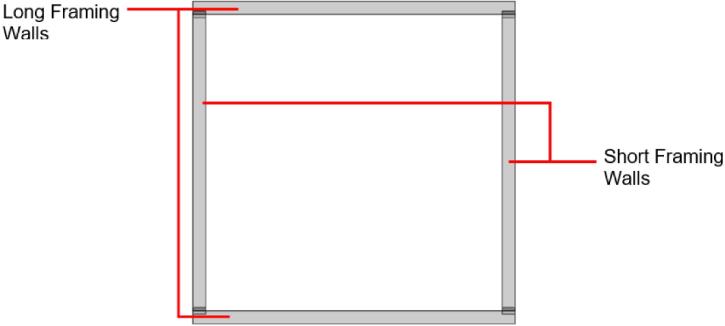
Corner alignment of the sheet mold assembly.
5. Follow up each tack weld with a complete weld along each bevel and a solid bead along the inside of each corner.
6. Grind the welds on the top and bottom face of the sheet mold frame down and round out the weld on the interior of each corner. Once smooth, the frame is complete.

The two surface sheets can be purchased from an external supplier at 11 7/8″ x 13″. For this build, scrap metal was marked with a metal scribe and sized in the brake from a single sheet. Once the surface sheets have been purchased or cut, and the sheet mold frame is welded, the sheet mold is complete and ready to be used.

## 6 Operation Instructions

While the applications of this device are only limited by the mold used, the primary purpose of this investigation is to a) demonstrate the benefits of using this machine to produce samples for characterizing the material properties of recycled plastics, and b) produce solid plastic sheets for use as stock material in any project. To achieve this, the following operation instructions detail the use of the press with both the ASTM D695 Mold for producing plastic samples complying with the ASTM D695 − Standard Test Method for Compressive Properties of Rigid Plastics and the sheet mold for solid sheets.

### 6.1 Safety Precautions

#### 6.1.1 Temperature

Note that this device operates at temperatures up to 300 °C. All handling must be done with gloves rated to above 300 °C for extended durations of time. Any operator is encouraged to test any gloves used with heat prior to directly handling the mold. Double layered gloves are equally encouraged. Long sleeves, pants, and a leather/welding apron can also be worn to protect against accidental contact and the radiative heat. Ensure that only thermally insulated materials contact the hot press or any metal surfaces that may be conducting heat. For plastics that produce fumes when heated (harmful or otherwise), a face mask must be worn in tandem with appropriate ventilation and filtration specific to the plastic being melted.

#### 6.1.2 Mechanical – Compression

Pressures up to 0.38 MPa are reached in the standalone press. Operators must be fully clear of the press prior to movement of the work head. As well, safety glasses must be worn to protect against any unexpected fracture of the plastic or work.

#### 6.1.3 Electrical

Do not handle any of the electronics within the electrical enclosure when the system is plugged into the wall regardless of whether the heating elements are active. High power electronics are used to power and control the heating elements and can be dangerous if contacted.

1. Remarks on Compliance and Safety Standards:
  a. General Compliance: Ensure that the entire electrical installation adheres to the latest versions of local and federal regulations for compliance and safety, particularly focusing on sections relevant to the specific application.
    i. For this build, the Canadian Electrical Code (CEC) [66] was used as a guideline.
    ii. Note that this is not a claim, certification, or guarantee that the described device adheres to all or any institutional, local, provincial, or federal regulations.
    iii. It should further be noted that this is in no way an exhaustive or comprehensive list of the required items to certify the device under any authority.
  b. Grounding and Bonding (CEC Section 10):
    i. Ensure effective grounding of all metal parts of the press to prevent electric shock. This includes the frame and any exposed conductive parts.
    ii. Use appropriate size and type of grounding conductor and ensure it is correctly connected to the system ground.
    iii. Verify bonding of all non-current carrying metal parts to ensure a low-impedance fault path.
  c. [Overcurrent] Protection and control (CEC Section 14):
    i. Confirm that the circuit breaker (12A or as appropriate) is correctly rated for the maximum continuous load, ensuring it does not exceed 80 % of the breaker's rating.
    ii. Ensure the breaker is suitably rated for the type of load and the characteristics of the heating elements.
  d. Wiring Methods (CEC Section 12):
    i. CEC Standard Compliance: Use wiring materials meeting CEC standards, with appropriate insulation, temperature ratings, and environmental suitability.
    ii. Wire Sizing: Select wire sizes for handling total load and transient conditions, in compliance with CEC Section 4, ensuring compatibility with overcurrent protection devices.
    iii. Conduit Installation: Follow CEC guidelines for correct conduit type, size, support, and fill, ensuring safe wire protection and operation.
  e. Control Equipment & Class 1 and Class 2 circuits (CEC Section 14 and 16):
    i. Ensure that the control circuitry, including the microcontroller and SSRs, comply with CEC standards for control equipment.
    ii. Consider the requirements for isolation and protection of control circuits.
  f. Thermocouple and Temperature Control, Class 1, and Class 2 circuits (CEC Section 16 and Applicable Standards):
    i. Ensure ungrounded Type K thermocouples are installed and used according to manufacturer specifications and CEC guidelines.
    ii. Implement appropriate safety measures for temperature control, including overtemperature protection and failsafe mechanisms.
  g. Electrical Equipment Approval (CEC Section 2):
    i. Verify that all electrical components used in the system are approved and labeled according to CEC standards and local regulations.
  h. Maintenance and Accessibility (CEC Section 2 and 26):
    i. Ensure that the electrical setup is accessible for maintenance and inspection, adhering to CEC guidelines for workspace and clearance.
    ii. Implement a routine maintenance schedule to ensure ongoing compliance and safety.
  i. Documentation and Labeling:
    i. Maintain accurate documentation of the electrical installation, including wiring diagrams and specifications.
    ii. Properly label all components and circuits for easy identification and compliance.

### 6.2 Machine Setup

#### 6.2.1 System Usage

The user interacts with the system by modifying a limited series of command variables outlined in Table 12. Each variable offers the user a degree of freedom over the systems heating profile and overall process duration.

**Table 12:** Command scheme for controlling the system via serial command line.

**Command**	**Description**	**Example**
st=	Set the target set temperature (in Celsius).	st = 200
dt=	Set the process duration time (in minutes).	dt = 60
t=	Set the current process time (in minutes).	t = 30
kp=	Set the PID proportional coefficient (Kp).	kp = 1.0
ki=	Set the PID integral coefficient (Ki).	ki = 0.05
kd=	Set the PID derivative coefficient (Kd).	kd = 0.01
start	Start a process (same as preheat)	start
stop	Stop the current process (same as standby)	stop
preheat	Manually enter the preheat state	preheat
heat	Manually enter the heating state	heat
cool	Manually enter the cooling state	cool
standby	Manually enter the standby state	standby
settings	Manually enter the settings state	settings

**Fig. 64:**
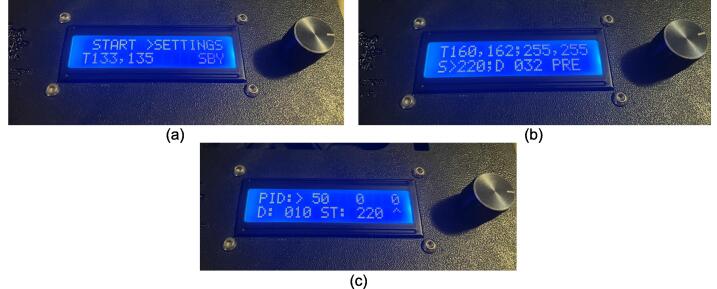
The Graphical User Interface in Various States: (a) The stanby state screen with the lower paten at 133C and the upper platen at 135C. (b) The (pre)heat state screen with set temperature at 220C, the lower plate at 160C, the upper plate at 162C, both outputs max (2 5 5), and 32 min remaining until the system begins cooling. (c) The Settings state screen with options for changing PID constants, duration, and set temperature.

For systems built with the LCD screen and rotary encoder the user can use the rotary encoder to navigate the menu system by rotating the dial to move the cursor and pressing the button to invoke an action (e.g. start a process, modify a variable, change states) as shown in Fig. 64.

**Fig. 65:**
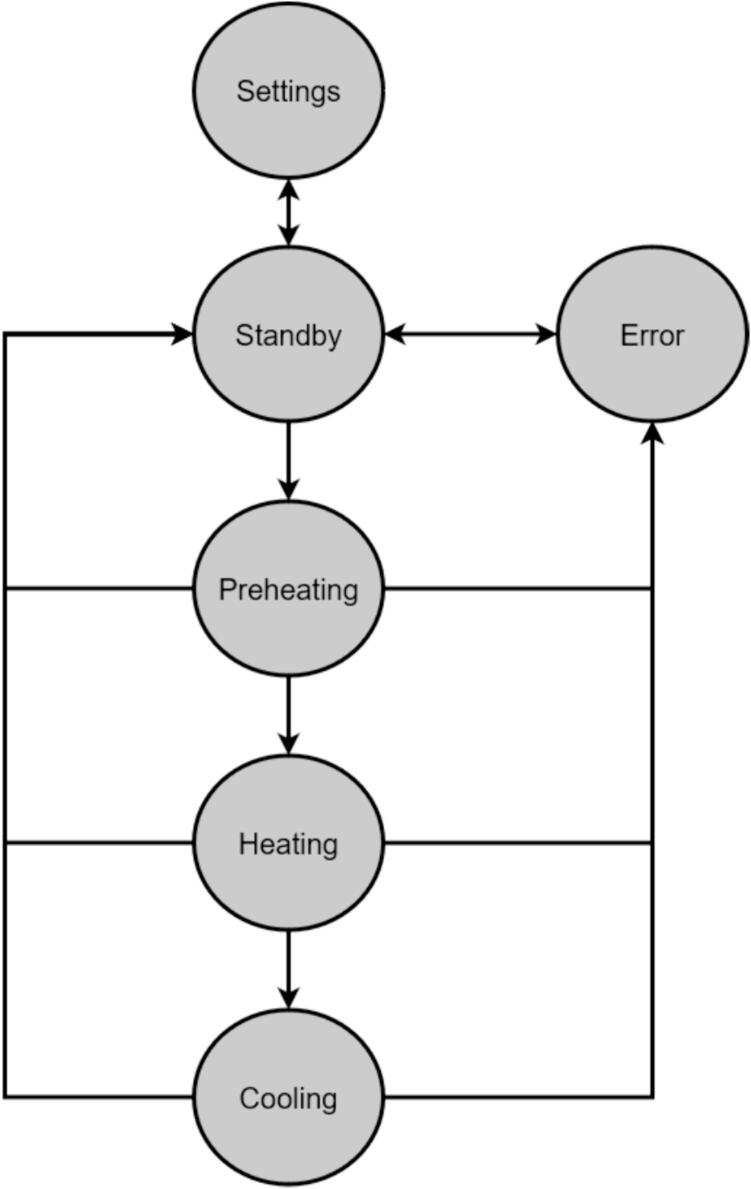
State Machine Diagram.

The software utilizes a state machine to guide the user between modes in a safe and easy manner. An overview of this logic is provided in Fig. 65. The user can press and hold the encoder button to return to standby from any state, effectively cancelling any active process. All state transitions are triggered by a serial command or the analogous GUI command. The error state is entered when faulty sensor readings or thermal run away is detected and must be cleared by the user before resuming.

#### 6.2.2 Mechanical Configuration

The following operation instructions align with the standalone assembly. Before beginning, ensure the jack is centered below the Jack Supports and the base is flush with the jack Linear Guides. Turn the release valve on the bottom of the jack all the way to the right to close it. Use the lever to actuate the jack and close the assembly until both Steel Platens are touching. This position will ensure the system reaches temperature in the shortest time.

If using only the upper and lower plates in conjunction with a hydraulic press, adhere to the following instructions with the only modification being to manually lift the upper plate assembly whenever the guide instructs releasing the jack to separate the Steel Platens and load a mold. Further, the upper plate assembly will be positioned on the work bed with the lower plate positioned on top so that the Jack Supports are in contact with the moveable work head of the hydraulic press.

### 6.3 ASTM D695 and D638 Mold Sample Production

Plug the machine into a 120 V outlet and flip the receptacle on the side of the electrical enclosure to ON. Adjust the set temperature to the melting temperature of the plastic being used and allow the machine to heat. Prepare the molds while the machine heats.

#### 6.3.1 Mold Preparation

Note that this section outlines the detailed method for producing samples using the ASTM D695 Mold, however, the same procedure can be applied to the ASTM D638 Mold. Due to the small footprint of the testing samples and the relatively large depth, the proportionally large grains of plastic regrind introduce the risk of voids in the final product. To combat this, a system of preheating the material in the press to soften it, compressing it, and then backfilling the mold before repeating was adopted to minimize the occurrence of voids. As a result, the mold is first loaded without the plugs and under no pressure to soften the material. In general, the amount of plastic by weight necessary to fill the mold should be calculated as a baseline. Using the approximate density of the plastic of interest and the mold volume of 025in^3^, the appropriate amount can be weighed and set aside for loading. Note that because recycled plastic has unknown exact properties, density may differ as well. Excess plastic must be added to account for this.

1. On a level surface, place the ASTM D695 Mold on top of the aluminum lid. Spray the walls of each rectangular pocket of the mold with silicon lubricant spray to assist in demolding.
2. Completely fill each pocket with waste plastic regrind.
3. Place the remaining Aluminum Lid onto the mold to cover the plastic.

#### 6.3.2 Loading the Mold

Once the hot press has reached the desired temperature as confirmed by the display, prepare to load the mold.

1. Slowly release the valve on the jack and drop the lower platen. Once the jack has reached the bottom of its stroke and the plate is resting on the lower plate supports, tighten the valve again.
2. Place the ASTM D695 Mold in the center of the Lower Plate.
3. Actuate the jack until the lid of the mold is flush with the upper platen and allow the plastic to soften for approximately 5 min.
4. Once the plastic has softened, remove the mold, and set it on a nearby heat resistant surface. If necessary, use a putty knife to pry off the lid and expose the plastic inside.
5. Moving quickly while the plastic remains soft, use pliers, a screwdriver, or a similar tool to manually press down the plastic in each pocket until there is vacant space above the compressed plastic layer.
  a. Backfill the mold with more plastic regrind, replace the ASTM D695 Mold Lid and return the mold to the hot press.

Repeat this process once more until the mold has been filled with the appropriate weight of plastic with some excess to account for incongruencies in recycled batch differences and flash (E.g., Initial loading, X2 refills).

#### 6.3.3 Compression Molding

1. Following the final mold refill, carefully place a Plug on each of the filled pockets, ensuring the plug does not contact the walls of the mold at any location. Place the second ASTM D695 Mold Lid on top and return the complete loaded mold to the hot press. Fig. 66 below shows an exploded view of the appropriate mold set up.

**Fig. 66:**
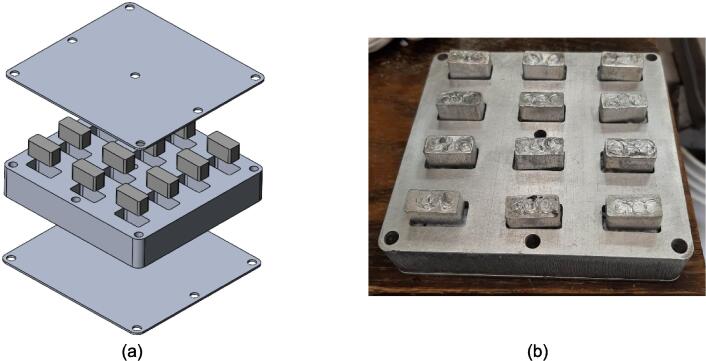
(a) Mold alignment and order of component placing. (b) Fully loaded mold and placed plugs prepared for final compression step [70].
2. Actuate the jack until the mold lid is flush with the upper plate assembly.
3. Allow the plastic to soften again for 5 min before slowly actuating the jack until the mold lid is fully compressed against the ASTM D695 Mold and the plugs are no longer visible. This position indicates that the plugs are fully depressed, and the achievable compressive load has been fully transferred to the plastic.
  a. Note: it is expected to observe some “overflow” of plastic oozing from the seam between the Mold lid and ASTM D695 Mold. This is a necessary feature of flash type molds that ensures plastic is flowing and effectively filling all cavities before excess plastic is ejected with any trapped air. This excess plastic is referred to as flash and is identified in Fig. 67a. Note that any flash observed should be minimal, and an excessive amount is an indicator of a procedural error.

**Fig. 67:**
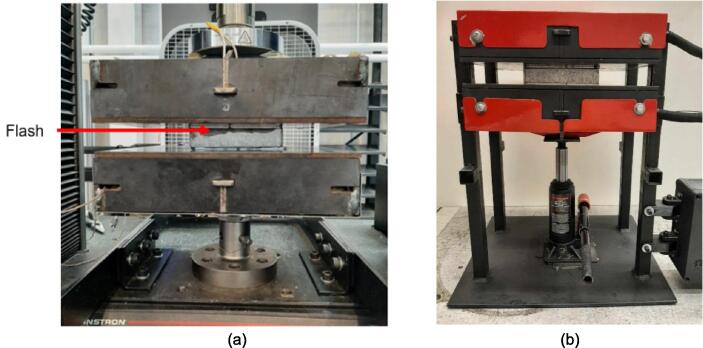
Fully compressed mold with appropriate flash overflow as shown in a (a) hydraulic press, (b) Standalone assembly.
4. Actuate the jack until it is under the maximum loading. Double check the pressure is appropriately maintained by attempting to actuate the jack periodically. Allow the plastic to remain under pressure at temperature for an additional 10–15 min.
  a. If using a hydraulic press, hold the machine at the compressive force that was required to close the mold in the previous step. This value will be dependent on how long the plastic was allowed to soften, the melt flow index (MFI) of the plastic, and the temperature the system was set at. For example, polycarbonate required 6000 N and was held in place for an additional 10–15 min. These values were approximated through trial and error.
  b. Note that the aluminum mold will expand substantially as it heats and result in an increase in the compressive force read by the hydraulic press.

#### 6.3.4 Cooling the Mold

The cooling procedure must be completed under pressure to prevent form shrinkage. Once the desired heating duration has been reached, set the machine to OFF and flip the switch on the receptacle. Place a box fan adjacent to the mold to promote airflow and convective heat transfer across the hot plates and mold. The cooling time to handling will depend on the material selected and melting temperature used but can be expected to be between 45 min to 1 h. Once the temperature reads well below the melt temperature of the plastic being used, the mold can be removed with the use of gloves.

#### 6.3.5 Demolding

**Fig. 68:**
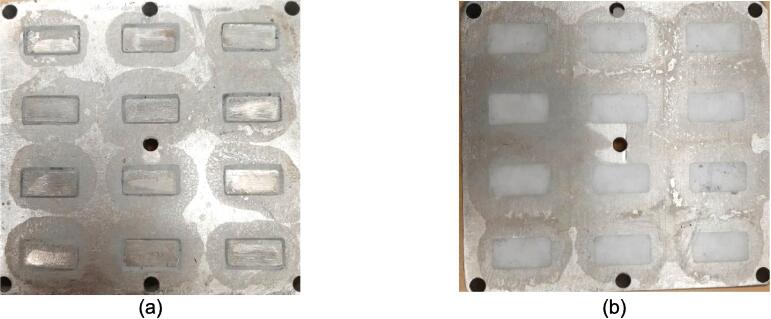
Released ASTM D695 Mold. (a) Top half of mold with plugs visible, (b) bottom side with solid PC.

The overflow flash will have securely adhered the top and bottom lid to the ASTM D695 Mold center. To release the plastic, secure the mold in a vice near the bottom edge, and carefully wedge a thin putty knife between the lids and the mold center. Use a hammer to push the blade farther into the seam and pry apart the lids. Incrementally rotate the mold until the entire lid has been released. The result can be seen in Fig. 68 below.

To remove the exposed samples from the mold, place the mold back in the vice. Position a bolt with a shank diameter less than 1/2″ against a plug and the grip of the vice to act as a plunger. On the opposite side, brace a piece of wood between the grip and the mold near the sample of interest. Slowly tighten the vice to force the bolt against the plug and eject the sample. Repeat for each sample.

**Fig. 69:**
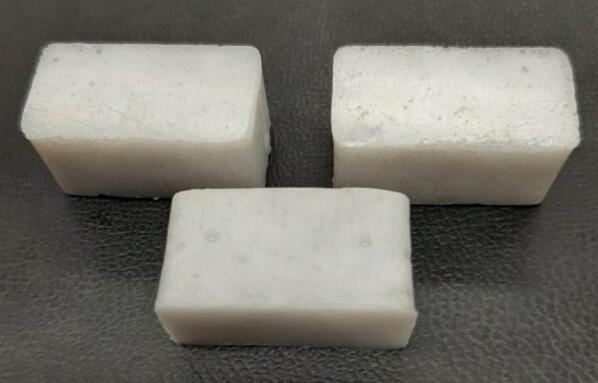
Resulting material samples prepared for testing.

Once each sample has been removed from the mold, use snips to remove the excess flash and sandpaper to clean up the edges of each sample. The solid samples are now prepared for material testing and shown below in Fig. 69.

### 6.4 Sheet Mold for Solid Plastic Sheets

#### 6.4.1 Mold Preparation

The mold design for the flat sheets is parametric and can be adjusted depending on the desired size. To test the dimensional extremes of the press an 11 1/4″ x 11 1/4″ x 1/4″ mold was used. These dimensions yield a plastic sheet with a volume of 31.64 in^3^ (518.48 cm^3^). Using this volume and the density of the plastic of interest, the total mass of the regrind required can be easily determined. Once the amount of plastic is determined, measure the required amount out and set it aside.

1. While the machine is heating, spray silicon lubricant onto both the top and bottom surface sheets. Use a paper towel to spread the lubricant evenly across the sheet surface and interior edge of the sheet mold frame before cantering the frame on one of the surface sheets. The sheet mold assembly orientation is shown in Fig. 70a.

**Fig. 70:**
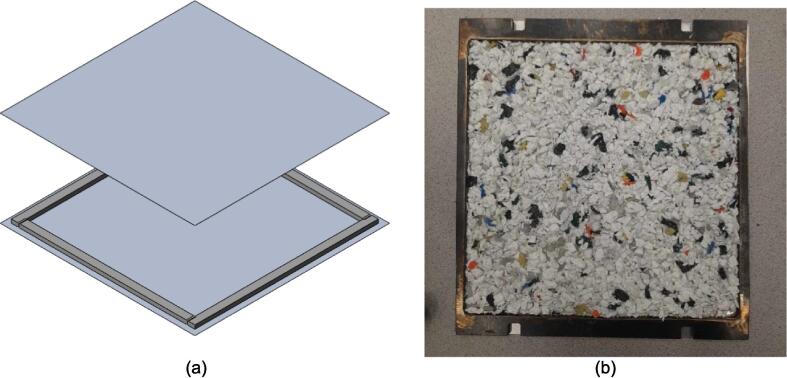
Sheet Mold (a) exploded view assembly and (b) physical loading using HDPE.
2. Evenly distribute the weighed plastic across the surface sheet within the confines of the sheet mold frame as shown in Fig. 70b. Note that the plastic will appear to overfill the mold due to the air gaps between flakes.
3. Release the jack valve to lower the bottom pressing plate to the lower pressing plate supports. Close the valve on the jack once the platens are fully separated.
4. Slide the loaded sheet mold frame and surface sheet onto the lower platen and center it as shown in Fig. 70b. Add the remaining surface sheet on top.
5. Actuate the jack until the upper platen contacts the lid of the mold.

#### 6.4.2 Compression Molding

1. Depending on the plastic used, allow it to soften for 3–5 min before applying pressure.
2. Increment the applied force until resistance is felt by the jack. As the plastic flows and melts, the force will drop. Continue to adjust the jack to maintain constant pressure until the mold is nearly, or entirely closed. Once closed, increase the force until the jack can no longer be actuated as shown in Fig. 71c.
  a. When using a hydraulic press, the load was increased to 10,000 N after 5 min, and increased to 20,000 N to close the mold for PC, as an example.

**Fig. 71:**
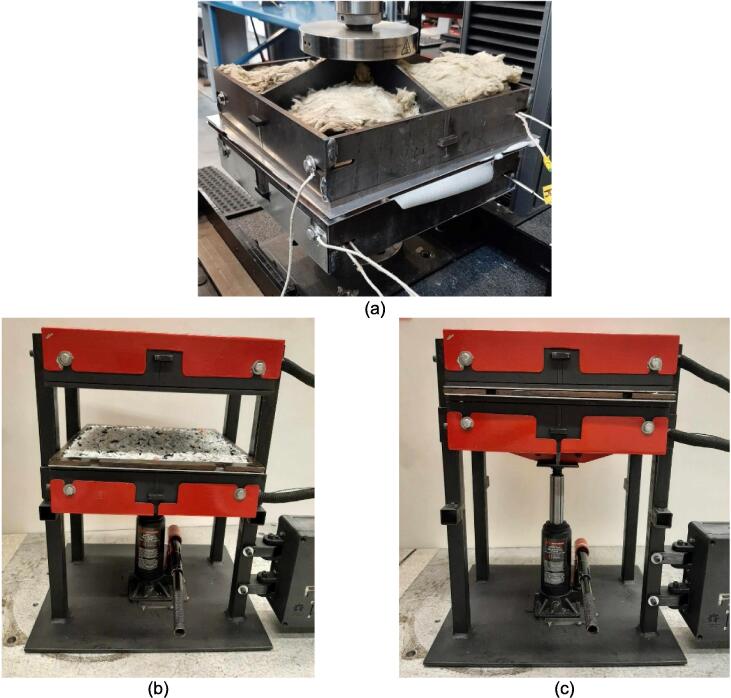
Loading of the sheet press and fully compressed sheet mold procedure. (a) Preliminary testing in a hydraulic press using polycarbonate and known loads, (b) loading of the sheet mold in the standalone support frame using HDPE, (c) fully compressed sheet.
3. Allow the mold to sit for 30 min under pressure. Continue to monitor the applied force and increase it if it drops as the plastic flows and settles.

#### 6.4.3 Cooling the Mold

Similar to the procedure for the ASTM D695 Mold, the machine can be turned off and the mold allowed to cool under pressure. Due to the increase in surface area and size of the sheet produced, the impact of shrinkage and resulting warping is much more prevalent. As a result, the mold would ideally reach room temperature before releasing pressure. Or, the mold can be removed once the temperature is below 100 °C, but allowed to fully cool outside the sheet press while remaining in its mold.

#### 6.4.4 Demolding

**Fig. 72:**
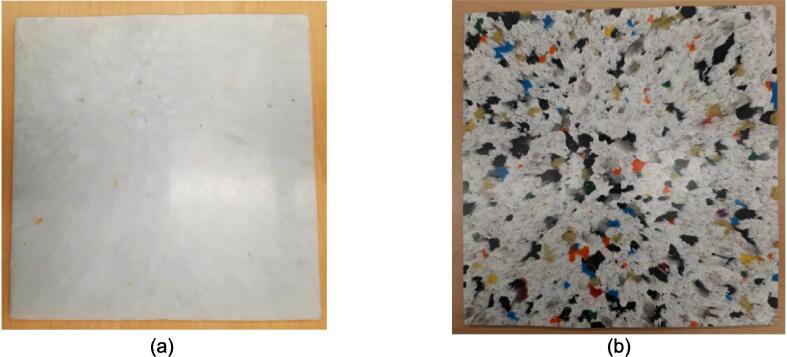
Resulting solid plastic sheets. (a) polycarbonate, (b) HDPE.

Once at room temperature, the surface sheets should easily separate from the plastic and mold frame revealing a solid sheet beneath. For HDPE, even shrinkage around the perimeter of the frame reached up to 3 mm, allowing the sheet to easily be pushed through the mold frame. Use snips to remove the large pieces of flash first and follow up with a deburring tool along each edge. The result will be a smooth, finished product as shown below in Fig. 72.

## 7 Validation and Characterization

### 7.1 Design Validation and Theoretical Optimization

The design and layout of the upper plate assembly, lower plate assembly, and support frame were governed by the loading conditions needed to compression mold thermoplastics at the desired platen size of 12″ x 12″ and ideally up to 1/2″ to 1″ in thickness. The loading required to achieve this is dictated by the MFI, or rather how much force is required to cause the melted plastic to flow through the mold. While typical industrial compression molding machines use standards to overestimate the force required, this design relied on the example set by Precious Plastics and Bachrach at Delft University of Technology (TU Delft). Based on the long ram jack adopted by Precious Plastics, their press saw pressures up to 11.4 PSI, while Bachrach adopted a higher pressure of 45.5 PSI [71]. As a result, the design stage was entered with the assumption that the desired loading would exist within this window verified by other machines, and would likely not exceed the TU Delft cap of 45.5 PSI. However, in the interest of guaranteeing the system could achieve the necessary loading cases, the system was designed to be more robust than even projected by the DFTI models. the closest commercial bottle jack loading increment that could reach and exceed 45.5 PSI was selected to provide an additional Factor of Safety (FOS). Further, this would allow the platens to be cable of withstanding higher loads in an external hydraulic press prior to building the support frame to dial in the loading necessary and confirm the loading claims made by Precious Plastics and Bachrach.

To support 45.5 PSI from Bachrach, the 12″ platen needed to be able to withstand approximately 29,000 N distributed across its surface. Maintaining the interest in having extra loading capability over Bachrach, and being constrained to the closest commercial bottle jack equivalent, a 4 ton (∼4000 kg) bottle jack was selected to provide approximately 55.5 PSI across the 12″ platen square footprint. To further constrain the finite element analysis (FEA) and design to guarantee the machine was capable and the “worst case” study conducted, the simulations were performed with the complete load case demanded for the sheet mold acting on the localized loading pattern created by the ASTM D695 mold. Finally, it is important to recall that the Center Supports were designed to be removable and offer 3 separate configurations for the machine. Maintaining this, the configuration with both Center Supports had to withstand the 4 ton loading case while the remaining two configurations with one Center Support and finally no Center Supports had to at a minimum achieve the Bachrach and Precious Plastics expectations, respectively. In this way, these simplified configurations could remain useful for a user with lower-stress molding applications and still had their effectiveness supported by the literature.

The following section discusses the FEA results and simulation setup for the three upper plate assembly configurations, the lower plate assembly, and the support frame. The results are summarized in Table 13. Each simulation was completed as a static study due to the small deformations and assumed unchanged stiffness. The material selected was AISI 1020 steel with a yield strength of 352 MPa to approximate the typical welded mild steel that may be purchased.

**Table 13:** FEA Results Summary for Upper and Lower Plate Assemblies.

Simulation	Loading	Max Displacement (mm)	Max Stress (MPa)	Factor of Safety
Fully Reinforced	39,226 N (4 ton)	0.014	337	1.04
1 Center Support	29,420 N (3 ton)	0.19	345	1.02
No Center Support	14,000 N	0.23	337	1.04
Lower Plate	39,226 N (4 ton)	0.016	76	4.63
Support Frame	15,580 N	1.72	346	1.01

To simplify the calculations within each simulation the parts were first all imported into a single part file, mated, and combined to force a “bonded” interaction between all components. To eliminate any potential stress singularities and likewise unrealistic results, all square corners were filleted.

#### 7.1.1 Configuration 1: Fully reinforced upper plate assembly with two Center Supports

The fixtures and loading setup were consistent across each upper plate assembly configuration aside from the changes to the load value as summarized in Table 12. The footprint of the ASTM D695 mold was sketched onto the pressing surface of the Steel Platen and incorporated as a separate selectable surface using a projected split line. This region was “fixed”. The 1” width of the Support Legs was similarly added using the split line feature around each mounting hole. These four regions were selected, and a “roller/slider” fixture added. The load was applied normal to the platen and along only the lower half of the mounting holes to better simulate the reaction force of a bolt in each hole. The case-specific load was distributed across all four mounting holes.

**Fig. 73:**
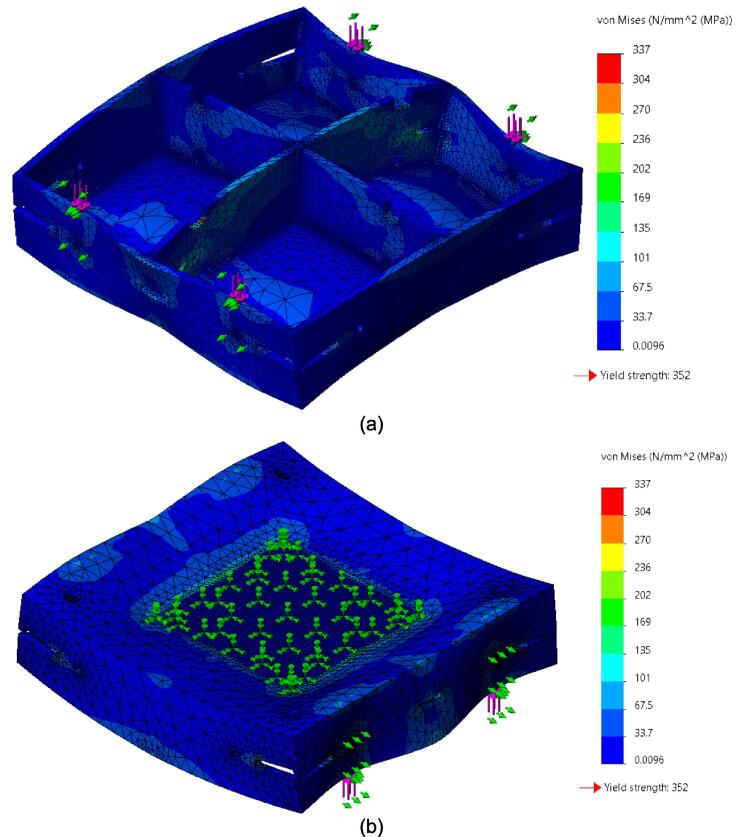
Fully reinforced upper plate assembly with two Center Supports stress response plot. (a) Rear side of platen and Center Supports, (b) pressing surface of the platen.

The fully reinforced configuration with two Center Supports demonstrated an overall load capacity of 39,226 N (4 tons) for the ASTM D695 mold. The molding capabilities of this configuration exceed both the precious plastic and Bachrach demands to support investigation into using thicker molds and forming larger quantities of plastic at once. The highest stress was observed as tension along the rear face of the Center Supports and the inside of the mounting holes as shown in Fig. 73.

#### 7.1.2 Configuration 2: Partially reinforced upper plate assembly with one Center Support

**Fig. 74:**
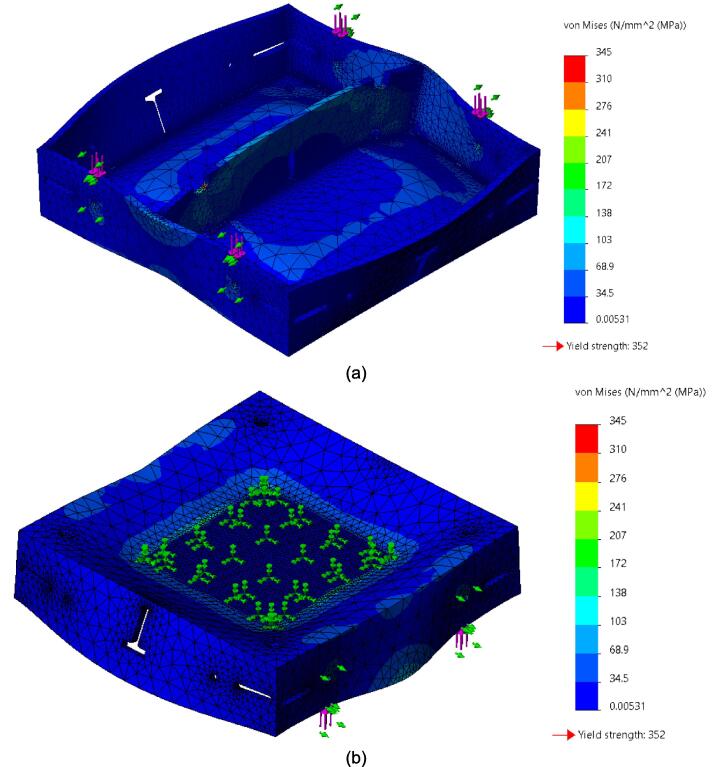
Partially reinforced upper plate assembly with one Center Support stress response plot. (a) Rear side of platen and Center Supports, (b) pressing surface of the platen.

With one reinforcing Center Support the overall load capacity of the upper plate assembly is reduced to 29,420 N (3 tons) for the ASTM D695 mold relative to the 4 tons observed with the fully reinforced assembly. This configuration offers molding capabilities based on the Bachrach testing demands of 45.5 PSI across the sheet mold with an additional FOS offered from using the smaller ASTM D695 mold footprint as opposed to the complete platen despite the same load. The highest stress was once again observed as tension along the back face of the Center Support as shown in Fig. 74.

#### 7.1.3 Configuration 3: Upper plate assembly with no Center Support and only Outer Framing Walls

**Fig. 75:**
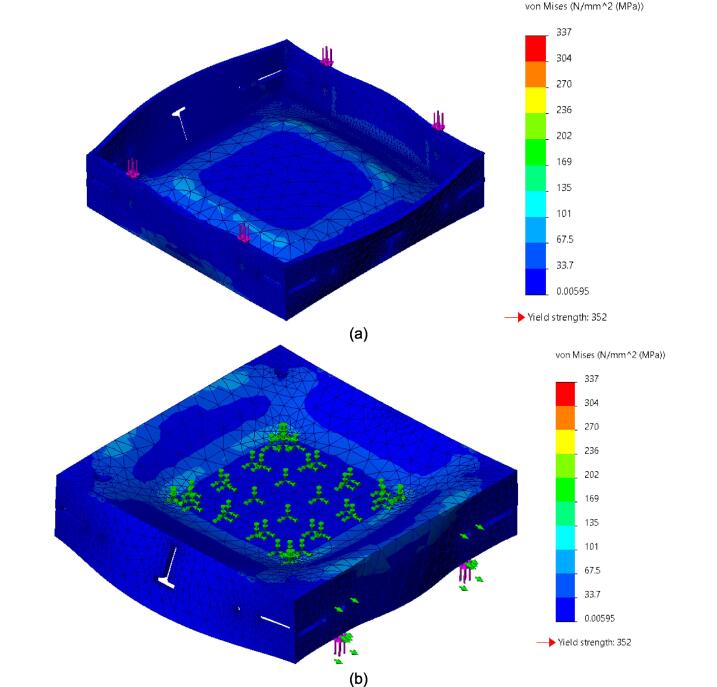
Upper plate assembly with no Center Supports stress response plot. (a) Rear side of platen and Center Supports, (b) pressing surface of the platen.

Once all Center Supports are removed the overall load capacity of the upper plate assembly is further reduced to approximately 14,000 N for the ASTM D695 mold. This configuration therefore offers molding capabilities more in line with the Precious Plastics testing demands of 11.2 PSI. The highest stress was observed at the edge of the mold on the Steel Platen as shown in Fig. 75. This confirms that the assembly would be able to support a larger mold more adequately such as the sheet mold where the edge of the mold does not apply as severe a localized stress near the unsupported center.

#### 7.1.4 Lower Plate Assembly

**Fig. 76:**
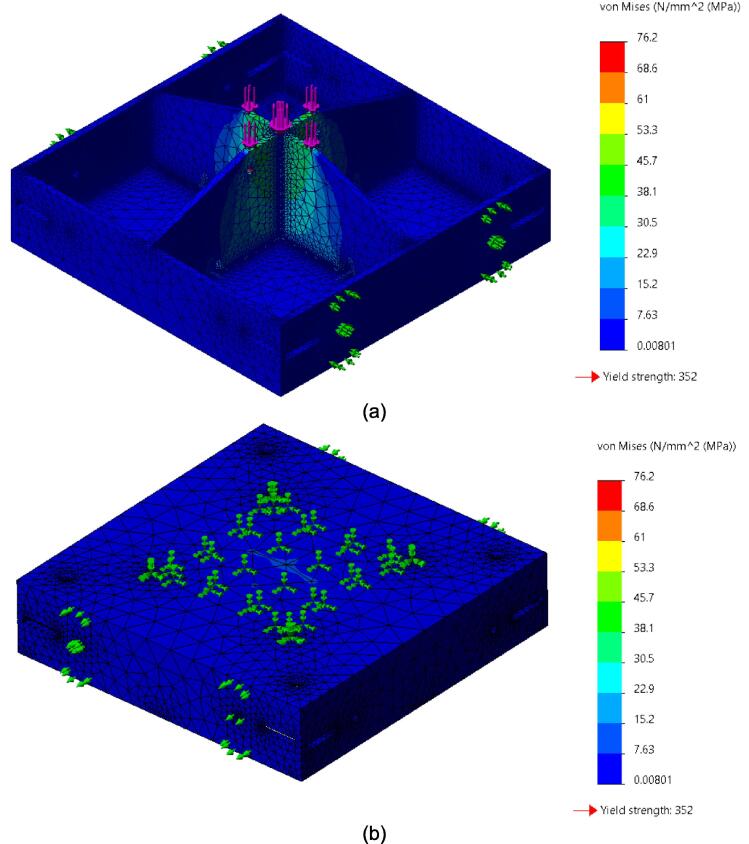
Lower plate assembly stress response plot. (a) Rear side of platen and Center Supports, (b) pressing surface of the platen.

The simulation setup of the lower plate assembly deviated from the previous upper plate assembly configurations. While the ASTM D695 mold surface remained fixed, the force was instead applied to the flat bottom of the “pyramid” created by the Jack Supports U1 and U2. The roller/slider fixtures were still included as the Support Legs constrain the outward deflection of the lower plate assembly. As this is the only configuration, the maximum force of 39,226 N (4 tons) was applied and a FOS of 4.2 observed as shown in Fig. 76.

#### 7.1.5 Support Frame Assembly

The support frame assembly was prepared separately from the upper and lower plate assemblies. Using a split line to select the footprint of the jack, the base of the jack was fixed. The mounting holes in each Support Leg were divided using a split line to apply the force normal to the base of the assembly and only on the upper half of each mounting hole. Notice the direction of this force is opposite to that used for the upper plate assembly as these are related and opposite reactionary forces. Finally, the Support Legs were constrained using roller/slider fixtures on the two inside faces of each leg where the upper and lower plate assemblies rest when in use. This ensures the distance between mounting holes and parallelism is maintained in the Support Legs despite not having the plate assemblies present in this simulation directly.

**Fig. 77:**
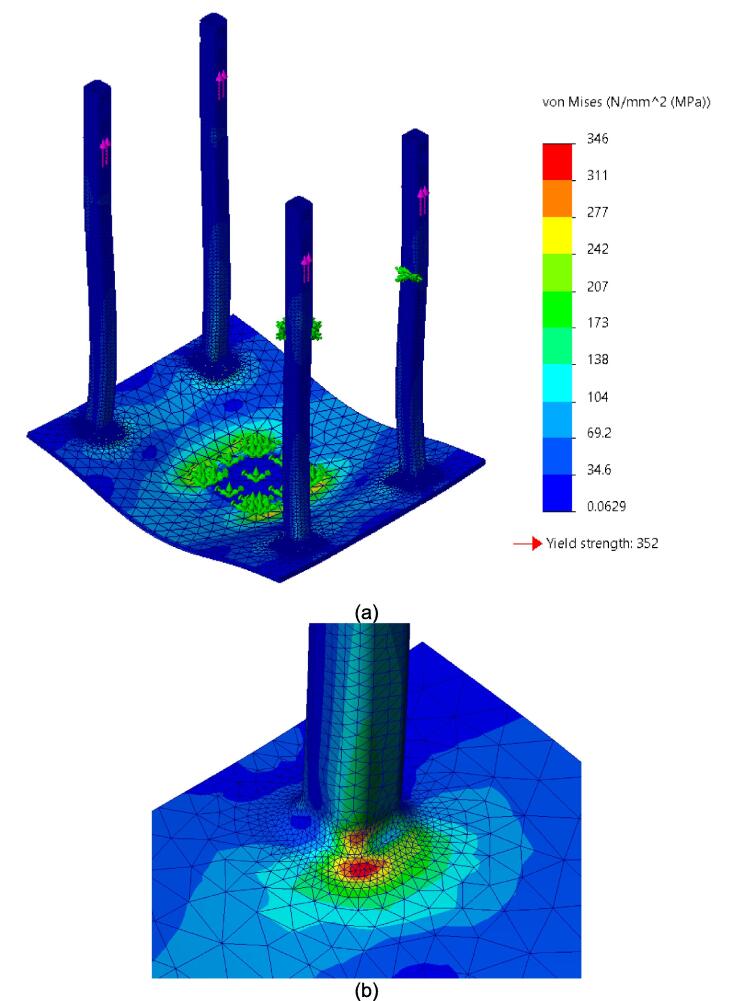
Support frame stress response plot. (a) complete bonded assembly, (b) emphasized maximum stress point at the base of each Support Leg.

Based on the loading cases established with the upper and lower plate assemblies when tested first in a separate hydraulic press, a loading case between Precious Plastics and Bachrach was shown to be successful and adopted. As a result, this support frame design with a maximum load capacity of 15,580 N was suitable. The failure point was located at the welded base of each Support Leg as shown in Fig. 77.

### 7.2 Mechanical Testing and Operation Performance

Two primary tests were conducted to validate the ability of the hot press to produce ASTM standard samples for material characterization and useful material stock for prosumer project builds. To accomplish this, the ASTM D695 mold was used to produce samples of polycarbonate regrind with unknown material properties which were then tested to quantify the compressive strength of the recycled batch of PC. This investigation was then completed by using the sheet mold to produce solid sheets of PC with known compressive strength to demonstrate how the press is intended to contribute to each stage of material recycling. It is important to note that while both the ASTM D695 and ASTM D638 molds were manufactured and can be used to characterize different material properties of a rigid plastic, only the ASTM D695 mold was used in machine validation. The method for producing samples with either mold is identical, and conducting material testing on the samples produced from either mold would appropriately demonstrate the ability of the press to produce samples for material characterization. As the objective is to validate the machine, formal testing of the ASTM D638 mold is not included here.

#### 7.2.1 ASTM D695 Sample Production and Validation

To validate the ability of the ASTM D695 mold and hot press to produce quality material samples, a recycled polycarbonate regrind was purchased and twelve samples prepared using the methodology outlined in section 6.3. The compressive strength of these samples was determined in a material testing machine and compared against the average material standard for extruded and molded polycarbonate.

To prepare the samples an appropriate set temperature for the press had to be selected. To accomplish this, a starting point of 300 °C was selected based on the reported melting temperature of 295–315 °C for PC [34]. The following parameters summarize the material specific details of the procedure used to prepare these samples to complement the methodology of section 6.3. Press Set Temperature: 300 °C.

• PC grain size: approximately 3–5 mm
• Operators*:* 1
• Press Heating Time: 40 min.
• Mold preparation Time:
  o Each pocket was loaded with material while the press was heating. Once the machine had reached the desired temperature, the mold was loaded without the plugs and allowed to soften for 5 min.
  o The mold was removed, and additional plastic added and stamped down an additional two times. In between reloading, the plastic was allowed to soften for 5 min in the press. (Softening at temperature X2 refills, 5 min each)
  o Each reload (X2) constituted an additional 5 min outside of the press for handling time. The final reloading involved adding the plugs on top of the stamped plastic.
  o Total time: 25 min
• Compression Time:
• 5 min softening with material fully loaded and plugs in place.
  o Incrementally actuated the bottle jack over 3 min until the mold was fully sealed.
  o This pressure was maintained for an additional 15 min and monitored the mold. If flash was visible or the mold and/or jack appeared to have settled, the force was increased.
  o Total time: 23 min
• Cooling Time:
  o The heaters were turned off, the fan turned on, and the system allowed to gradually cool under pressure.
  o The mold was removed at 50 °C after 45 min.
• Total Procedural Time: **1.5 h** for molding.

In terms of cycle time, the primary limitations of this procedure are associated with the time involved for heating and cooling the system, demolding each sample, sample cleanup, and mold cleanup. The 1.5 h outlined above does *not* include the time to demold each sample which is a typically involved process. In future iterations, the ASTM D695 mold could be further optimized for part ejection.

**Fig. 78:**
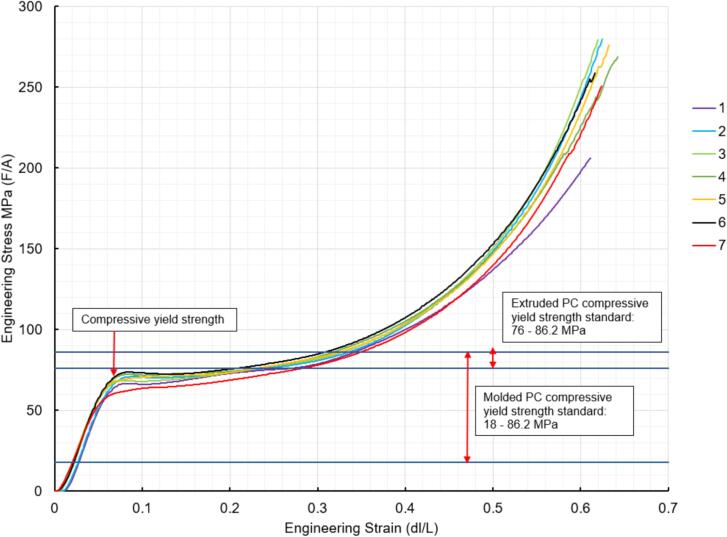
Engineering stress vs. strain for polycarbonate regrind as compared against the standard compressive strength ranges for molded and extruded PC.

The resulting 1/2″ x 1/2″ x 1″ polycarbonate samples were subjected to compressive testing at a rate of 1.3 mm/min in accordance with ASTM standard D695 [72]. Each resulting sample was weighed and inspected for defects that would produce outlier results such as surface voids or porosity. Of the twelve samples molded, seven samples were selected and compared against the average expected values for solid polycarbonate to quantify the compressive strength of this batch of recycled polycarbonate. The results are summarized in Table 14 and Fig. 78.

**Table 14:** Polycarbonate Material Testing Property Summary.

Sample	Mass (g)	Density (g/cm^3^)	Ultimate Compressive Strength (MPa)
Extruded Standard [73]	4.92	1.20	18–––86.2
Molded Standard [74]	4.92	1.20	76–––86.2
1	4.79	1.17	66.72
2	4.81	1.17	70.97
3	4.75	1.16	68.58
4	4.84	1.18	72.30
5	4.87	1.19	71.15
6	4.87	1.19	73.67
7	4.60	1.12	64.14
Average (1–7)	4.79	1.17	69.65
Maximum	4.87	1.19	73.67
1 σ Standard Deviation	0.087	0.021	3.10

The compressive yield strength of the samples was compared against the average compressive yield strength for molded PC between 18–86.2 MPa and extruded PC between 76–86.2 MPa [73], [74]. These ranges are identified as horizontal datum lines in Fig. 78. The compressive yield strength of each sample can be identified as the maximum value of the curve preceding the observable plateau. The recycled samples produced in this study offered an average compressive yield strength of approximately 70 MPa which falls well within the top 25 % of the molded PC standard range, and just short of the extruded PC standard range. This variation between samples of the same thermoplastic and different forming methods further emphasizes the necessity for having a machine that offers the ability to produce material samples for quantifying these characteristics. Further, the low, but non-normal standard deviation across mass/density and compressive strength, reflects the degree of variation that can be expected from recycled plastic. While some degree of porosity is to be expected due to the manufacturing method, the location and volume of these voids plays a substantial role in the associated compressive strength of the sample. Therefore this production method demonstrates repeatable consistency between samples, while also reflecting the reality of property variation between plastic batches. In this case, having tested this batch of recycled material can provide confidence that the PC has competitive compressive properties to other molded PC products and is close to extruded values.

**Fig. 79:**
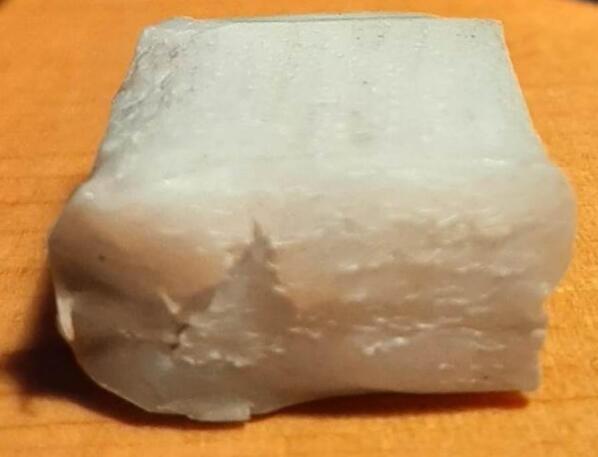
Compressed polycarbonate sample at failure.

In addition to compressive yield strength, the density for each sample was approximated and compared against the average density or PC of 1.20 g/cm^3^ [73], [74]. This was a critical feature of the tests as the primary molding failure observed was porosity and surface voids. The presence of voids presents the largest detriment to overall sample strength and is the largest concern when compression molding. The yield strength of a sample is directly proportional to the diameter of an internal or edge void and acts as the point of origin for crack propagation which will ultimately end in part failure. The site of these defects demonstrates the final failure point once the crack reaches the surface of the sample as shown in Fig. 79. The highest compressive strength achieved correlated to the densest sample at 73.67 MPa and 1.19 g/cm^3^. Therefore, this test demonstrated the efficacy of both the hot press and ASTM D695 mold for preparing samples for material testing and the necessity for gauging the quality/grade of plastics as the material properties of waste plastic can vary dramatically between batches.

The mold used for producing these samples was based on the cost-effective method of flash-type molds for short-run, small parts. These molds rely on overcharging the cavity with material to encourage flash ejection. While this is beneficial in terms of mold simplicity, part removal, and cost, these molds typically result in inconsistent densities and nonideal mechanical and physical properties. This is due to the applied compression being used to force flash flow as opposed to increasing the cavity pressure [75]. The mold was originally designed with the intention of the flash flowing up and around the undersized plugs, and to allow for easy manufacturing and part removal by having a separate upper and lower lid relative to the center cavity. This separation between the ASTM D695 Mold and the upper and lower lids, however, prevented the flash from flowing along the desired path. Instead, flash often escaped out of the bottom of the mold before it had a chance to flow upward and around the plugs. To combat this, the mold could be reimagined as a semipositive or positive mold type to reduce or eliminate the amount of flash and increase part pressure [75]. While these methods would certainly improve the density and material properties, the small size of these samples relative to the large surface area in contact with the mold poses a substantial challenge to part ejection. To avoid the necessity of adding ejector pins or complex mold features, the next iteration can be limited to only an upper and lower mold half with very little flash akin to a semipositive closure type.

Finally, to improve the pressing procedure, a smaller grain size of plastic regrind can be used to avoid introducing any voids during initial softening and mixing. A typical mold loading case in industry involves the placement of a solid, pre-softened charge on the lower mold half [75]. Due to the constraints of this mold size and the use of plastic regrind, this is not possible. As a result, the softening of the regrind and simultaneous manual stamping within the mold before loading the plugs is necessary to simulate this idyllic charge case. To supplement this part of the method, the material could be cycled through a smaller grinder to achieve a reduction in grain size and a related reduction in the size of void between grains and more the sample-wall interface.

#### 7.2.2 Sheet Production and Process Optimization

Once the recycled PC had been characterized using the ASTM D695 Mold, the procedure outlined in Section 6.4 was used to produce a series of PC sheets. The purpose of these sheet trials was to dial in the exact amount of plastic, pressing temperature, and duration time necessary for producing an optimized stock sheet of PC with known material properties. This trial period would be necessary for any new plastic used to ensure the procedure is tailored for the properties of each plastic to ensure the highest quality product.

**Fig. 80:**
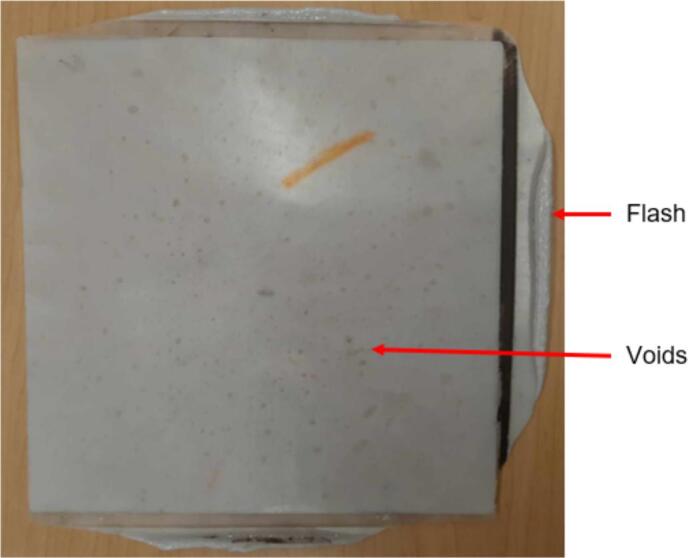
Common mold failure indicative of excessive temperatures being used during pressing. Key features to note are the distinct internal voids scattered across and the substantial flash along each edge.

The methodology established relied on using the average density of 1.17 g/cm^3^ derived from the previously prepared samples in conjunction with the known volume of the 11 1/4″ x 11 1/4″ x 1/4″ mold (204.13 cm^3^) to determine the appropriate mass of plastic required. As a result, 607 g of PC was weighed out in advance of pressing and used for each sheet. While the loading, molding, and cooling procedure was the same between each sheet trial, the temperature was incrementally decreased by 10–20 °C increments until the product quality was optimized. Early trials demonstrated the dramatic effect of temperature on surface quality, porosity, and flash production. At excessive temperatures, the plastic became aerated, flowed too freely, and overproduced flash leaving the product highly porous as shown in Fig. 80. For PC, the temperature was initially set to 300 °C as was used for the ASTM D695 Mold, before being reduced between sheet trials until the ideal temperature of 220 °C was identified. This involved observing the surface quality of each sheet at varying temperatures and identifying the transition point between a surface with large, blended flow lines diverging from the center under high heat, to a surface with distinct grains and surface voids in a cold product. The optimized temperature ensured a much denser product with a smooth, semi-homogenous surface finish, little flash, and therefore limited necessary post processing.

**Fig. 81:**
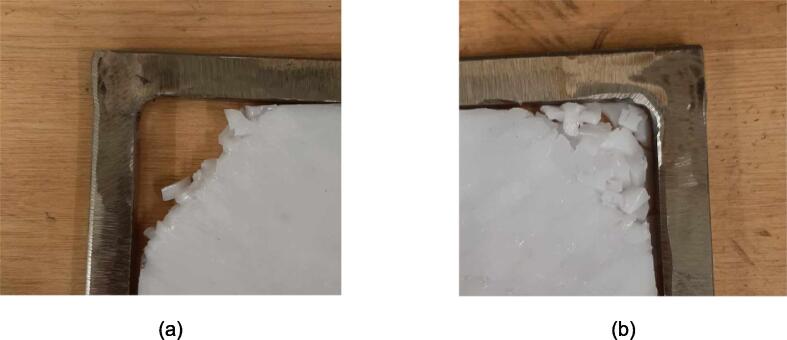
Common mold failures observed when optimizing the methodology of using the sheet mold. (a) an underfilled mold and (b) combination of an insufficient pressing time, too large a mold, and too low a surface temperature.

Another common molding defect observed was the stagnation of plastic at the corners of the mold. The plastic either did not reach the corners of the mold as shown in Fig. 81a, or did not melt and homogenize as shown in Fig. 81b. In the first case this was a result of an underfilled mold and indicated the need for extra material to be loaded to account for the exiting flash. In the second case, the temperature was too low at the corners. While this could be corrected by increasing the overall temperature of the press, this often resulted in superior corner penetration at the expense of a porous center. As a result, it can instead be concluded the press would benefit from a sheet mold redesign for PC that occupies a smaller fraction of the Steel Platen as the surface will always be coolest at the corners.

Ultimately, the sheet trials demonstrated the necessity for dialing in the settings of the press for each plastic and mold used, common molding defects and how to remedy them, and finally the ability of the hot press to produce consistent quality stock material sheets.

## 8 Applications

The primary goal of the open-source cold and hot scientific sheet press was to offer researchers a method of forming samples suitable for characterizing the material properties of unknown quality waste plastic regrind on a laboratory scale. The small-scale press and ASTM D695 compression mold and ASTM D638 tensile mold can be used to efficiently produce ASTM standard samples for material testing without heating and cooling an unnecessarily large work surface. Offered second to this is the broad ability to also produce small consumer products and/or sheets for general use. Both principal applications have been validated using reground waste PC though the machine, molds, and methods can be applied to any variety of thermoplastic and thermoplastic blend. At this stage, the press has been used to produce ASTM D695 PC-sand composite samples to investigate its potential as a concrete substitute [70] and to produce PC and high density polyethylene (HDPE) sheets for use as a building material in any 2-D or 2.5-D projects. Of these investigations, the production of PC-sand samples clearly demonstrated how the sheet press in conjunction with the ASTM D695 mold can be used to prepare multiple different sample compositions simultaneously and impact research applications. Use of this press ultimately allowed researchers to prepare 50 % sand and 50 % PC composite samples with which they could proceed to prove the competitive compressive strength of these blends against high performance concrete [70]. The applications of this study could extend into the production of sand-plastic bricks capable of outcompeting traditional bricks in terms of water absorption, lightweighting, and compressive strength [76], [77], [78], [79], [80]. With a variety of molds, the hot press could produce viable sand bricks, cinder blocks, paving stones, sidewalks, roof tiles, solar photovoltaic array footings, and other concrete substitutions. With the functionality of the system fully realized and the products fabricated of appropriate quality, the ASTM D695 and ASTM D638 molds can be applied to various waste plastics, mixtures and composites.

Alternatively, when considering direct applications of the plastic products from the sheet mold, the opportunities are only restricted by the post processing tools available and the designer’s creativity. These waste plastic sheets are ideal for use in any project where they can be thermally formed (bending), cut, interlocked, and assembled to form 2.5-D shapes. These applications can be as simple as shelving and storage or tailored more specifically to personal projects. In the build of this machine alone, the usefulness of sheet fabrication was demonstrated in the electrical enclosure for both artistic purposes in the lid and functionality on the rear cover. Commercially, 12″ x 12″ x 1/4″ HDPE sheets can be purchased new for $30 CAD on Amazon [81] or from waste plastic for $25 through the Concordia Precious Plastic Project in Montreal [82]. Alternatively, HDPE regrind can be purchased for $0.70 CAD/lb form Post Plastics and an equivalent sheet produced with the sheet mold for $0.87 CAD (with the cost being even less if the material is collected, sorted, and reground personally using an open-source granulator [83]). While this does not include labor and energy cost, a prosumer can produce a commercial material for less than 1/30th the market value. Similarly, the sheets can be used almost directly from the press with minimal post processing in structural applications including floor tiles, counters, or backsplash. Using commercial flooring as a material cost comparison, and vinyl tiles as the most inexpensive flooring advertised, flooring can be purchased for $1.39CAD/sq. ft. [84]. This price would be reduced by 30 % to $0.99 CAD/sq. ft if waste PC was purchased at $0.72 CAD/lb and pressed to produce a 1/4″ thick tile [85].

When looking at the characteristics of PC sheets specifically, significant advantages over conventional plastics make them desirable for more niche applications including solar cell encapsulation [86]. Researchers have suggested using thin PC sheets as a replacement film to traditional back-sheet films due to their high toughness, flexibility, and temperature resistance. The process for encapsulating these cells also demands the use of laminating equipment to seal each layer together. Extrapolating the demands of this product and manufacturing process, the hot press could at a minimum be applied to produce the PC thin film back-sheet, but also has the potential to be applied during encapsulation/lamination. Preliminary tests with the machine have demonstrated the ability to plastic weld components together to form a watertight seal by intentionally limiting the contact points between the plastic component and the platens to only where a weld is desired. In doing so, only the regions of the components being welded together that are in contact with the metal will heat and fuse. Not only does this expand the applications of the hot press into component fabrication for solar cells, but also solar encapsulation itself. Simultaneously, this identifies an additional capability of the machine for use in plastic welding.

Finally, not only can the press be applied to the compression molding of thermoplastic regrind, but the basic functionality as both a hydraulic press and heat source increases the variety of applications. Independent of the thermal capabilities, the pressing surface and 4-ton bottle jack allow the machine to be used for basic cold forming and crushing. This can be used for preparing larger plastic items for shredding by crushing them into a more manageable size for a traditional shredder or applied to basic sheet metal forming. Plastic with high strength and durability such as PC can be used to 3-D print profiled molds for forming the sheet metal. The sheet metal can be inserted between the profiled 3-D printed mold base and the mirrored plug, and the press closed to force the sheet metal to adopt the negative of the mold. This methodology can be used to quickly and repeatedly make customized brackets or enclosures for custom projects. Extrapolating this, once the molds have served their purpose, they can be crushed, shredded, and reused to form new sheets in the hot press. Therefore, the press provides valuable capabilities as a tool throughout the manufacturing process across a variety of applications.

## 9 Future Work and Directions

Extended operation has revealed opportunities to improve both the capabilities and ease of use of the machine. To improve the general ease of mechanical assembly, the primary obstacle encountered was the need for more clearance in the Outer Framing Walls for wiring the heating elements and thermocouples. The end slots would have benefited from additional length and width to provide ample clearance for the live, neutral, and ground wires, thermocouple, and cable management sheath. With more clearance, the interior nuts can also more easily be accessed for bracing with a screwdriver and the bolts tightened. As well as clearance adjustments, all interior features should have included dog bone fillets to better accommodate the manufacturing method and improve the general fit of interlocking components. To promote ease of use by the operator, the design would benefit most dramatically from the transition to a pneumatically or electrically powered jack to reduce user strain and actuation time.

To allow the design to be more easily tailored for different users’ size requirements, the design could be transitioned to parametric based on the desired platen size. This could be as detailed as requiring the user to input their specific heating element size and overall platen footprint, and increasing the lattice count depending on the scale desired. While this machine is intended for small applications and largely as test equipment for investigating material behavior prior to larger application, the machine would also benefit from a moderate increase in size such that the items produced could be at least 12″ x 12″ without forcing the mold to reach the very extreme edge of the platen. Further, this would allow the machine to accommodate more commercially available 6″ oven coils in place of the current strip heaters to dramatically reduce the cost and improve accessibility. This would also ensure more even heating across each platen and therefore yield fewer molding defects involving grain separation at the corners of each sheet mold.

Finally, the system heat loss was not monitored or controlled along the exposed mold edge between platens. External shielding with a reflective internal coating could have been added around the outside to assist in retaining heat and more consistently maintaining the system’s temperature for a more energy efficient process. This removable shielding would also act as a protective barrier for both general use and against any projectile parts ejected when using the machine as a cold press for crushing.

While these minor changes would significantly improve the operator experience during manufacturing, assembly, and general use, the machine itself can continue to be modified to increase the functionality. At this scale and with a new sheet mold of less than16-gauge thickness, the hot press can be used to produce thin sheets for use in vacuum forming. Taking this a step further, the existing structure of the hot press can be further modified to include an integrated vacuum former and increase the application of the machine to an additional method of thermoforming and therefore an additional product realm. Due to the nature of the press already including a fixed heated upper plate, the machine would only require a part changeover routine for lower plate in place of a vacuum table to achieve this dual functionality. In this case, a configuration with both pressing plates can be used for the preliminary production of thin stock material, followed by the replacement of the lower plate with the vacuum table. The upper plate can be used to heat and soften the thin sheets before sliding the sheet along the Support Legs and down over the vacuum table. To further increase function and reduce cost, the molds used on the vacuum table can be 3-D printed and later broken down to form new sheets once they have reached the end of their useful life. In this case, the press would have the ability to tackle more applications and supply the stock material necessary for a shorter and more direct order of manufacturing. It would also be contributing to a tight circular economy [87].

The primary next step for this machine and project is to graduate from a small-scale hot and cold press targeting sample production and low volume/size items to a scaled-up production press with a 4 ft x 4 ft (1.2 m x 1.2 m) platen capable of processing large quantities of waste plastic. This natural evolution will aim to be a comparable alternative to the Precious Plastics model that can be manufactured with more appropriately sourced imperial metal stock. As well, the size increase would allow the applications to extend into larger structures and, more notably, specifically suit a standard solar panel to promote waste plastic use in solar racking solutions [88], as well as parametric open-source cold-frame agrivoltaics systems (POSCAS) [89], and surgical fracture tables [90]. Outside of these existing open-source applications demanding large stock sheets, applications could include more commercial products such as Muskoka chairs and picnic tables. At this pressing size, not only is the recycling of plastic high, but the scale of application potential increases dramatically. Moving in this direction, the lessons learned from this smaller scale and the material data collected can more appropriately be put toward more impactful projects that reduce polymer waste and promote recycling using a distributed methodology.

## Funding

This work was supported by The Natural Sciences and Engineering Research Council of Canada and the Thompson Endowment.

## CRediT authorship contribution statement

**Morgan C. Woods:** Writing – review & editing. **Cameron K. Brooks:** Writing – review & editing. **Joshua M. Pearce:** Writing – review & editing.

## Declaration of competing interest

The authors declare that they have no known competing financial interests or personal relationships that could have appeared to influence the work reported in this paper.
